# Supplementary material for: Sugar-sweetened beverage intakes among adults between 1990 and 2018 in 185 countries
Source: Nat Commun. 2023 Oct 3;14:5957. doi: 10.1038/s41467-023-41269-8 (PMC10614169; doi:10.1038/s41467-023-41269-8)
Supplement: Supplementary file 1 — Supplementary Information [file 41467_2023_41269_MOESM1_ESM.pdf]

Sugar-sweetened beverage intakes among adults between 1990 and 2018 in 185 countries

Supplementary Information

Supplementary Information

Table of Contents

Supplementary Methods 1. Description of data assessment, standardization, and analysis. 4

Supplementary Table 1. Definition and units of sugar-sweetened beverages§. 8

Supplementary Table 2. Group and individual mean plausibility cutoffs for sugar-sweetened beverages 8

Supplementary Methods 2. Covariates 8

Supplementary Methods 3. GDD Estimation Model 11

Supplementary Figure 1. Flowchart of number of countries with dietary surveys identified, standardized, and included in the Bayesian hierarchical model. 18

Supplementary Table 3. Countries by world region (superregion) in GDD 2018. 19

Supplementary Table 4. Characteristics of global data sources of sugar-sweetened beverage intakes............ 20

Supplementary Table 5. Number of dietary surveys for SSB intake globally, regionally, and in the 25 most populous countries by time period. 21

Supplementary Table 6. National mean (95% UI) sugar-sweetened beverage intakes (8 oz servings/week) in adults (20+ years) by sex, age, education, and area of residence in the 25 most populous countries in 2018. .. 22

Supplementary Table 7. Absolute Difference in sugar-sweetened beverage intakes (8 oz serving/week) by sex, education level, and area of residence globally and regionally in adults (20+years) across 185 countries in 2018. 23

Supplementary Table 8. Absolute Difference in sugar-sweetened beverage intakes (8 oz serving/week) in males vs. females in the 25 most populous countries in adults (20+years) in 2018. 24

Supplementary Figure 2. Global and regional sugar-sweetened beverage intakes (8 oz servings/week) by age among females (a) and males (b) adults (+20 years) in 2018. 25

Supplementary Table 9. Absolute Difference in sugar-sweetened beverage intakes (8 oz serving/week) in high vs. low educated adults in the 25 most populous countries in adults (20+years) in 2018. 26

Supplementary Table 10. Absolute Difference in sugar-sweetened beverage intakes (8 oz serving/week) in urban vs. rural areas in the 25 most populous countries in adults (20+years) in 2018. 27

Supplementary Table 11. Global and regional mean (95% UI) sugar-sweetened beverage intakes (8 oz serving/week) in adults (20+ years) by age, sex, education, and area of residence across 185 countries in 1990. 28

Supplementary Table 12. Global and regional mean (95% UI) sugar-sweetened beverage intakes (8 oz serving/week) in adults (20+ years) by age, sex, education, and area of residence across 185 countries in 2005. 29

Supplementary Table 13. National mean (95% UI) sugar-sweetened beverage intakes (8 oz servings/week) in adults (20+ years) by sex, age, education, and area of residence in the 25 most populous countries in 1990. .. 30

Supplementary Table 14. National mean (95% UI) sugar-sweetened beverage intakes (8 oz servings/week) in adults (20+ years) by sex, age, education, and area of residence in the 25 most populous countries in 2005. .. 31

Supplementary Figure 3. National mean sugar-sweetened beverage intakes (8 oz servings/week) in adults (20+ years) across 185 countries in 1990 (a) and 2005 (b). 32

Supplementary Figure 4. Global and regional sugar-sweetened beverage intakes (8 oz servings/week) by age in adults (+20 years) in 1990 (a) and 2005 (b). 33

Supplementary Figure 5. Global and regional sugar-sweetened beverage intakes (8 oz servings/week) in adults (+20 years) by area of residence in 1990 (a) and 2005 (b). 34

Supplementary Figure 6. Global and regional sugar-sweetened beverage intakes (8 oz servings/week) in adults (+20 years) by education and area of residence in 1990 (a) and 2005 (b)....... 35

Supplementary Figure 7. Absolute change in mean sugar-sweetened beverage intakes (8 oz servings/week) in adults (20+ years) in the 25 most populous countries from 1990 to 2018. SSBs were 36

Supplementary Table 15. Absolute change in mean sugar-sweetened beverage intakes (8 oz servings/week) from 1990-2005, 2005-2018, and 1990-2018 in adults (20+ years) by sex, age education, and area of residence and by world region across 185 countries....... 37

Supplementary Discussion 1. Trends over time by age, sex, education, and urbanicity within the 25 most populous countries 38

Supplementary Table 16. Difference in mean sugar-sweetened beverage intakes (8 oz servings/week) in adults (20+ years) from 1990-2005, 2005-2018, and 1990-2018 by sex in the 25 most populous countries....... 39

Supplementary Table 17. Difference in mean sugar-sweetened beverage intakes (8 oz servings/week) in adults (20+ years) from 1990-2005, 2005-2018, and 1990-2018 by age in the 25 most populous countries....... 40

Supplementary Table 18. Difference in mean sugar-sweetened beverage intakes (8 oz servings/week) in adults (20+ years) from 1990-2005, 2005-2018, and 1990-2018 by education level in the 25 most populous countries. 41

Supplementary Table 19. Difference in mean sugar-sweetened beverage intakes (8 oz servings/week) in adults (20+ years) by area of residence from 1990-2005, 2005-2018, and 1990-2018 by area of residence in the 25 most populous countries. 42

Supplementary Methods 1. Description of data assessment, standardization, and analysis.

Data assessment

Data received from corresponding members or from publicly available surveys were checked to confirm survey-level characteristics; dietary intake variables were categorized into GDD dietary factors; necessary unit and format conversions were noted. Data-owners or survey directors were contacted extensively to resolve questions about data quality, categorization, or assessment methods to ensure accuracy and completeness of data prior to analysis.

Preliminary data checks

Biostatisticians generated survey description files for each survey including survey characteristics, variable lists, and summary statistics for categorical and continuous variables. Research assistants used these description files to assess inclusion of survey level information and demographic variables. Discrepancies between author-reported characteristics and those ultimately included in the dataset were noted for further discussion with the data-owner.

Categorization of variables into GDD dietary factors

Research assistants matched reported dietary data to GDD dietary factors. This involved categorizing foods, nutrients, mixed dishes, and regional items into the matched GDD dietary factor, noting cases where variables represented less than the optimal GDD definition. Unit conversions were included as necessary to transform variables into the optimal GDD units.

Categorization of variables into GDD dietary factors – Food Frequency Questionnaires

To transform food and beverage data reported from Food Frequency Questionnaires into optimal GDD units, most often grams per day, all categorical variables were standardized into single daily serving units. When ranges of frequencies were provided, the mean of each range was utilized to represent each frequency category. Variables reported in times per week were divided by 7 to calculate the average daily servings. Variables reported in times per month were divided by 30.42 (the average number of days in a month) to calculate the average daily servings. In cases where the upper range was open-ended (e.g., "5 or more times per week"), the ranges of the other frequency categories were used to calculate an upper limit. Servings per day were then converted into grams per day by multiplying the number of servings by the author-reported serving sizes or by the GDD standard serving sizes.

Communication with data owners and creation of data key

Any questions regarding the data, including those about region-specific diets (e.g., disaggregation of mixed dishes, classification of regional items), survey-level characteristics, and serving sizes for foods and beverages, were communicated to the data owner. After all questions regarding the data were answered, research assistants generated a data key outlining all available variables of interest, including demographic and dietary variables. Categorizations of dietary variables were turned into STATA code for clear identification and research assistants flagged the quality assurance checks.

Converting household data to individual data

Household-level data were transformed into individual-level data using the Adult Male Equivalent (AME) method. The AME method estimates individual-level intakes by assigning each household member a reference AME based on their age and sex. Household members' reference AMEs are summed to find total household AME. Each individual's reference AME is then divided by the total household AME to find individual-level AME. This individual-level AME represents the proportion each individual contributes to the overall household AME. This

individual level proportion is multiplied by the household consumption of each food item to estimate individual-level intake.

Data aggregation

Using preliminary checking documents provided by research assistants, biostatisticians converted individual-level data into aggregated outputs for each dietary factor stratified by the available demographic variables. Stata version 12 was used to convert all demographic and dietary variables from raw data files to a single data file containing only relevant variables. Missing observations were excluded from the dataset and all variables were recoded to match the GDD demographic and dietary variable coding scheme. Data were then aggregated into demographic strata by age, sex, residence, education, and pregnancy/lactating status. We used education as a measure of socioeconomic status, as based on expert consultation this was felt to be a more stable and comparable measure than other variables which can be reported with significant error and underreporting in different countries (e.g., household income). In addition to the single, final data file, supporting files were generated including a summary report, minimum/maximum values for each dietary factor, and group level means, standard deviations, and percentiles of intake. All output files were stored in each survey's specific folder on the Tufts GDD Box, accessible to all members of the research team.

Energy adjustment

We extracted both raw and energy-adjusted data when available. If energy adjustment of individual-level data had not been completed by the data owner, biostatisticians completed energy adjustment at the aggregation stage to age-specific levels using the residual method. This approach was considered the "gold standard." We adjusted for total energy intake to mitigate the effects of measurement error in data collection, account for differences in energy requirements related to body size, metabolic efficiency, and physical activity, and facilitate comparisons between surveys, age groups, and sexes.

Total daily energy values by age:

- 0-0.9 years = 700 kcal
- 1-1.9 years = 1,000 kcal
- 2-5 years = 1,300 kcal
- 6-10 years = 1,700 kcal
- 11-74 years = 2,000 kcal
- 75+ years = 1,700 kcal

Child and older adult-specific daily energy values were selected using dietary recommendations and mean population ranges from the USA, United Kingdom, and India.

Energy adjustment corrections

We initially asked that all data be shared both unadjusted and energy-adjusted to 2,000 kcal, regardless of age category, but retrospectively changed this decision to reflect the age-specific levels. When possible, energy adjustment using the residual method was repeated to reflect these changes. In some cases, this approach was not possible, and thus alternative approaches for energy adjustment correction were taken.

Energy adjustment correction of aggregate ("stratum-level") data

In some cases, data were provided or accessed at the stratum level (i.e., age group, sex, education level, etc.). In these cases, energy adjustment correction depended on whether 2,000 kcal/day-adjusted values had previously been provided by the data owner. If energy-adjusted data had been provided, a simple ratio of the age-specific level to 2,000 kcal was applied post-hoc to convert the value to the correct energy level. If stratum-level data were only provided in an unadjusted format but with corresponding total energy intake, intake was adjusted to the age-specific energy level using the energy density method, in which a simple ratio of reported calorie intake to age-specific level was applied to the unadjusted value. If stratum-level data were provided in age groups which traversed more than one level of age-specific energy intake, a weighted mean daily energy intake was calculated. This weighted mean

daily energy level was then used to adjust intake using the ratio readjustment method. If only unadjusted intake was available, the energy density method was used.

Energy adjustment of data without adjusted values or total energy intake
In limited cases, individual-level data were not initially energy-adjusted or provided with mean caloric intake data, precluding the use of the gold standard and ratio readjustment methods. In these instances, daily per capita energy availability data from Food and Agriculture Organization (FAO) Food Balance Sheets (FBS) were used to inform stratum-level caloric intake. In short, country-year-specific FBS energy data were adjusted using coefficients derived from a multivariate linear regression of GDD input data, FBS data, and both regional and survey-level covariates. Adjusted FBS energy was then corrected to the prescribed energy level by applying a factor of the energy level's proportion of 2,000 kcal. Unadjusted food and nutrient intake values were then adjusted with this corrected energy intake via the energy density method.

Quality control

Data integrity and quality were assessed at each step during survey collection, processing, harmonization, and analyses. Duplicate reviews were performed of recorded survey characteristics, demographic variables, dietary definition classifications, and unit conversions. To assess for outliers and validity (errors) in reported intakes, plausibility thresholds were defined for each dietary factor, both at the individual level and stratum (e.g., group mean) level, based on dietary reference intakes, tolerable upper limits, toxicity ranges, and existing regional data on mean intakes in populations. Any value identified as potentially implausible was reviewed for extraction errors, followed by direct correspondence with the corresponding member or public survey data owners, to detect and correct potential errors. Data remaining implausible after such steps were excluded from final datasets. Results for each dietary factor were further graphed and visually inspected by country, age, sex, dietary assessment method, representativeness, and time, reviewing survey result plausibility and consistency within and across countries.

Data finalization

After data has been finalized for inclusion, it was stored within the Access database, which houses information on all surveys, corresponding authors, and survey checking statuses. Aggregated data was collated by dietary factor and prepared for input into the GDD prediction model.

Protocol for converting FFQ frequency data into GDD servings

1. Step 1- Standardize the categorical frequency variables to a single daily serving unit
    a. If a range of frequencies is given, take the mean ("Avg") of the range
    b. If the frequency is presented in times/week, divide by 7 (for days in a week)
    c. If the frequency is presented in times/month, divide by 30·42 (average days in a month)
        i. *Note:* If the category is presented as days/week instead of times/week, assume one serving per day and treat as times/week
        ii. Example A) 5-7 days/week = (6 days/week) / (7days/week) = 0·857 servings/day
        iii. Example B) 1-3 times/month = (2 times/month) / (30.42 days/month) = 0·066 servings/day
    d. If the upper range is open ended, use the range of the other frequency categories in the survey to create an upper limit and then take the average of that range.
        i. Example: "5 or more times per week" where next lowest level is 2-4 times per week. Assume a range of 5-7 times per week, take the average (6 times per week)/(7 days/week) = 0·857 servings/day
2. Step 2- Convert servings to grams
    a. If available, survey-specific serving sizes were used for conversions.
    b. If survey-specific serving sizes are not available, ask the data owner for usual, country-specific serving sizes.

c. If data owner does not provide country-specific serving sizes, utilize country-specific serving sizes identified from national agencies (e.g., USDA).
d. If no country-specific serving sizes are identified, use the GDD standard serving size conversions.

Common categories of intake and their servings per day conversions

- Never
  - Calculation: 0; Daily Serving: 0
- Occasional-Few times/year
  - Calculation: Should capture the range of values between never and the next highest choice based on the data set.
  - Serving: Depends on next level categorization.
- Less than once a month (1-11 times per year)
  - Calculation: 1+11=12/2=6 Avg servings/year; 6/12 months=0.5 servings/month; 0.5/30.42 days
  - Daily serving: 0.0164
- 1-3 times/month
  - Calculation: 1+3=4/2=2 Avg servings/month; 2/30.42 days
  - Daily serving: 0.066
- 1/ week
  - Calculation: 1 servings/7days
  - Daily serving: 0.143
- 2-4 days/week
  - Calculation: 2+4=6/2=3 Avg servings/week; 3servings/7days
  - Daily serving: 0.429
- 5-6 days/week
  - Calculation: 5+6=11/2= 5.5 Avg servings/week; 5.5/7days
  - Daily serving: 0.786
- 5-7 days/week
  - Calculation: 5+7=12/2=6 Avg servings/week; 6/7days
  - Daily serving: 0.857
- 1/day
  - Calculation: NA; Daily serving: 1
- 2-3/day
  - Calculation: 2+3=5/2= Avg 2.5 servings
  - Daily serving: 2.5
- 4-5/day
  - Calculation: 4+5=9/2= Avg 4.5 servings
  - Daily serving: 4.5

Common weight conversions

1 Kilogram = 100 grams
1 Ounce *Cannot use for fluid ounces = 28 grams
1 Pound = 454

Standard serving sizes for sugar-sweetened beverages

- Variable code: V15
- Variable name: Sugar-sweetened beverages
- Reference serving sizes: 2003-06 US NHANES (median) = 368 g per serving
- "Usual" average serving sizes (g/serving): Adults and children/adolescents older than 2 years = 248; 12-24 months 130; 6 to 11 months 84

Supplementary Table 1. Definition and units of sugar-sweetened beverages[§].

Dietary factor	Unit	Preferred definition	Alternative definition
Sugar-sweetened beverages	g/day	Total sugar-sweetened beverage intake, defined as any beverage with added sugars having ≥50 kcal per 8 oz (236.5 g) serving, including commercial or homemade beverages, soft drinks, energy drinks, fruit drinks, punch, lemonade, and frescas. This definition excludes 100% fruit and vegetable juices and non-caloric artificially-sweetened drinks.	Optimal (our condition), including fruits and vegetable juices.

[§] These definitions were used for extracting/requesting SSB intake data.

Supplementary Table 2. Group and individual mean plausibility cutoffs for sugar-sweetened beverages

Group or individual	Age group	Serving size	Mean minimum serving/day	Mean maximum serving/day	Mean min g/day	Mean max g/day
Group	Children/ Adolescents	248 grams	≤ 0	6	≤ 0	1488
	Adults	248 grams	≤ 0	4	≤ 0	992
Individual	Children/ Adolescents	248 grams	≤ 0	15	≤ 0	3720
	Adults	248 grams	≤ 0	15	≤ 0	3720

Supplementary Methods 2. Covariates

Covariate identification

We identified country- and time-specific covariate data from various sources to further inform our model estimates. These data supplement our individual-level dietary intake data, particularly in countries for which these inputs are limited. We consulted experts and conducted comprehensive searches of publicly available databases to identify >800 covariates. We prioritized approximately 400 covariates for testing:

Data source*:	Year(s)
• UN FAO food balance sheets	1980 - 2018
• Harvard Global Expanded Nutrient Supply (GENuS)	1980 - 2011

• Principal component analysis of FAO and GENuS data	2013
• Euromonitor fat and oils sales data	1998 - 2018
• World Bank Gross Domestic Product (GDP)	1980 - 2018
• World Bank unemployment rate	1980 - 2015
• World Bank Gini coefficient	1980 - 2015
• World Bank poverty rate	1980 - 2015
• Barro Lee education	1980 - 2010
• World Bank precipitation	1982 - 2014
• CIA Factbook latitude	N/A
• CIA Factbook land area	N/A
• CIA Factbook coastline ratio	N/A

*Percentage of country-years imputed for each covariate source: GDP=3%; unemployment rate=7%; food balance sheets=11%; fat and oil sales data=11%; GENuS=26%; Gini coefficient and poverty rate=28%; education=87%.

We conducted principal component analysis (PCA) using the 'princomp' function in R separately for: 1) 23 grouped FAO food balance sheet (FBS) foods, beverages, and energy, 2) 142 GeNUS foods, and beverages, and 3) 19 GeNUS nutrients and energy. The first four components from each PCA were considered for covariate testing.

Covariate imputation and truncation

If covariate data were missing for some (but not all) years of a given country, we used linear interpolation to fill in those years. Covariate data sources that ended before 2018 were imputed using a moving average of the three most recent values to obtain values for all covariates through the year 2018. Region-level means were assigned to countries for which entire covariates were missing. To assess validity of the imputations, we imputed non-missing values with the same model and visually compared observed vs. imputed values via scatter plots.

The GDD prediction model operates on the natural log scale (except for dietary factors measured as proportions), including the covariate data. To minimize a strong effect of very small values for covariates with a broad range of values on the log scale having an outsized influence on modeled estimates, we truncated covariate data on the non-transformed scale using the following rules:

For covariates with a 95th percentile value

1. > 3.5: Truncate values < 0.5 to 0.5
2. ≥ 1 and ≤ 3.5: Truncate values < than 0.1 to 0.1
3. < 1: No truncation

Covariate testing

For each dietary factor, we calculated the correlations between covariates and original survey-level stratified mean dietary intakes, and we selected up to 10 covariates for model inclusion, favoring those with the highest correlations, a mix of food/nutrients and other covariates, and sensible links to the dietary factor.

Each of the covariates identified in the correlation stage (maximum 10 covariates) and the four PCA components were then included in a stepwise regression to test for inclusion in GDD models, with an entry point p-value of <0.299 and an exit point of >0.30 during the stepwise process. These stepwise regressions resulted in three nested versions of the GDD model per diet factor:

1. Base model: Closest diet factor proxy from FAO or GENuS (1-2 covariates per model)
2. Restricted model: All covariates with $p<0.1$ from the results of the stepwise regression plus base model covariate(s).
3. Inclusive model: All covariates from the results of the stepwise regression plus base model covariate(s).

For each dietary factor, five-fold cross-validation was used to compare model fit for the three versions of the GDD model. Data were split into five partitions at the survey level: four partitions making up the training dataset, and the remaining segment as the testing data. The models were fit to the training set, and resulting outputs were compared to training set to assess model fit via calculating expected log predictive density (ELPD).[1] This was repeated five times so that each partition was used once as the training set.

Final model selection and included covariates for sugar-sweetened beverages

Sugar-sweetened beverages:
- Model: Base model (model 1)
- Covariates: FAO sugar and sweeteners

Supplementary Methods 3. GDD Estimation Model

1 Data Overview

Survey data was collected and collated from across the globe. Mean intakes were estimated from each survey by subgroup (age group, sex, urbanicity, education level), which we use as our primary inputs for the model. These data have an inherently nested hierarchical structure which makes a multilevel approach to modeling data appealing. The hierarchical structure of the data we assumed was as follows: countries were nested in world regions (superregions), which are nested in the globe.
Our model uses the following seven world regions:

- CEECA (Central/Eastern Europe and Central Asia)*
- HIC (High-Income Countries)
- LAC (Latin America and Caribbean)
- MENA (Middle East and North Africa)
- SA (South Asia)
- SEEA (Southeast and East Asia)*
- SSA (Sub-Saharan Africa)

*In prior GDD reports, the region Central/ Eastern Europe and Central Asia was called the Former Soviet Union, and Southeast and East Asia was called Asia.

Additionally, a host of country-year level data was collected for potential use as predictor variables. A list of these variables, and their sources, can be found below. Note that surveys are rarely fully stratified by all four subgroups of interest. The model uses the most granular stratifications available as input. The availability and level of stratification has been described elsewhere.

2 Model description

The GDD estimation model aimed to estimate mean intake of 53 dietary factors in 185 countries by, country/year/age/sex/urbanicity/education, by synthesizing survey mean intake data from sources of varying quality. For the estimation of SSB intakes, our model used 451 surveys, mostly nationally or subnationally representative, collected at the individual-level, and representing 87.1% of the world's population. The Bayesian multilevel framework has some advantageous properties that are appealing for our purposes. Namely,

- "Shrinkage" of parameter estimates towards an overall mean. For example, mean estimates for data sparse countries are pulled towards the region mean, allowing for more reasonable estimates for countries with potentially unreliable data.
- Intuitive framework for predicting means (with uncertainty bounds) for countries with no available data.
- Ability to include prior knowledge about intake through priors.
- Allows for model flexibility and complexity often not granted in similar frequentist approaches due to difficulty in optimization.

Fundamentally, our model is a Bayesian model on the log-means of intake with a nested hierarchical structure (it clusters countries within world regions and world regions within the globe), assuming exchangeability between countries and between world regions. Using this structure allowed us to borrow strength across units, a concept commonly known as "partial pooling". In partial pooling, each country's mean estimate borrows from the other countries' data within the region, resulting in shrinkage of the country mean estimate towards the region mean. The less informative the data were for a particular country, the more pooling there is. To this structure, we added sex, urban/rural area of residence, education, and non-linear age effects (also within a nested hierarchical structure), survey and country-level covariates through 2018, and overdispersion on study-level variance to account for non-sampling variation. It borrows heavily from models presented by Finucane et al.[3] and Flaxman et al.[4] For dietary factors that were measured as proportions of energy intake, we use $-log(log(y_i))$ as the link function instead of $log(y_i)$.

Below we provide a full mathematical description of the model, with detailed descriptions for each component, but first, we present some notation:

Subscript notation:

h: age/sex/educ/urbanicity group
i: study
j: country
k: world regions

Superscript notation:

c: country
s: world regions
g: globe

2.1 The model

$$f(y_{h,i}) \sim N(a_j + b_{1,j} sex_{h,i} + b_{2,j} u_{h,i} + b_{3,j} educ_{h,i} + \gamma_j(z_{h,i}) + X_i\beta, SE_{h,i}^2 + \tau_i^2)$$

Where,

$f(y)$	$\Leftarrow -log(-log(y))$ for dietary factors measured as proportions, $log(y)$ otherwise
$y_{h,i}$	$\Leftarrow$ mean intake level for stratum h in study i
a_j	$\Leftarrow$ country-specific intercept, for country j
$b_{1,j}$	$\Leftarrow$ country-specific difference between females and males, for country j
$sex_{h,i}$	$\Leftarrow$ variable indicating whether the $y_{h,i}$ corresponds to an all-male group (0), all female group (1), or mixed (0.5), for stratum h in study i
$b_{2,k}$	$\Leftarrow$ region-specific difference between urban and rural, for region k
$u_{h,i}$	$\Leftarrow$ variable indicating whether the $y_{h,i}$ corresponds to an all-rural group (0), all urban group (1),or mixed (% urban), for stratum h in study i
$b_{3,k}$	$\Leftarrow$ region-specific education effect, for region k
$educ_{h,i}$	$\Leftarrow$ two variables indicating whether $y_{h,i}$ corresponds to low education (defined to be 6 years or less of schooling if mixed), proportion of low education, and high education for stratum h in study i
γ_k	$\Leftarrow$ non-linear age-trend, for region k
$z_{h,i}$	$\Leftarrow$ midpoint age, for stratum h in study i
$X_i\beta$	$\Leftarrow$ study + country level covariate effects
$SE_{h,i}^2$	$\Leftarrow$ standard error of $f(y_{h,i})$ (estimated via delta method), for stratum h in study i
τ_i^2	$\Leftarrow$ overdispersion parameter, for study i

2.2 Intercept, sex differences, education differences, and urban/rural differences

We fit a multi-level model with 3 levels (countries nested in world regions nested in the globe) for intercepts and sex differences, and 2 levels (world regions nested in the globe) for age pattern, education differences, and urban/rural differences. Mathematically, this can be described as follows:

$$a_j = a_j^c + a_{k[j]}^s + a^g$$
$$b_{1j} = b_{1j}^c + b_{1k[j]}^s + b_1^g$$
$$b_{2j} = b_{2k[j]}^s + b_2^g$$
$$b_{3j} = b_{3k[j]}^s + b_3^g$$

$$a_j^c \sim N(0, \kappa_a^c), b_{1j}^c \sim N(0, \kappa_{1b}^c),$$
$$a_k^s \sim N(0, \kappa_a^s), b_{1k}^s \sim N(0, \kappa_{1b}^s), b_{2k}^s \sim N(0, \kappa_{2b}^s), b_{3k}^s \sim N(0, \kappa_{3b}^s)$$

where,

a_j ⇐ country-specific intercept, for country j
a_j^c ⇐ country-specific random effects, for country j
$a_{k[j]}^s$ ⇐ world region-specific random effects, for country j in world region k
a^g ⇐ global intercept
$b_{1,j}$ ⇐ country-specific sex effect, for country j
$b_{1,j}^c$ ⇐ country-specific sex random effects, for country j
$b_{1,k[j]}^s$ ⇐ world region-specific sex random effects, for country j in world region k
b_1^g ⇐ global sex effect
$b_{2,j}$ ⇐ country-specific urban effect, for country j
$b_{2,j}^c$ ⇐ country-specific urban random effects, for country j
$b_{2,k[j]}^s$ ⇐ world region-specific urban random effects, for country j in world region k
b_2^g ⇐ global urban effect
$b_{3,j}$ ⇐ country-specific education effect, for country j
$b_{3,j}^c$ ⇐ country-specific education random effects, for country j
$b_{3,k[j]}^s$ ⇐ world region-specific education random effects, for country j in world region k
b_3^g ⇐ global education effect
κ_a^c ⇐ between-country variance, at the country level
κ_{1b}^c ⇐ between-country sex variance, at the country level
κ_a^s ⇐ between- world region variance, at the country level
κ_{1b}^s ⇐ between- world region sex variance, at the country level
κ_{2b}^s ⇐ between- world region urban variance, at the country level
κ_{3b}^s ⇐ between- world region education variance, at the country level

Note that $b_{3,j}$ is a vector of two parameters, one for the difference between low and middle education and one for the difference between high and middle education. Also note that the model assumes between-country variance was the same across all world regions. Likewise, education, urban/rural differences and age patters are assumed to be the same for countries within a world region.
Weakly informative priors are used for the hyper-parameters: half-Normal (0, 0.5) for the κ parameters, for a^g, b_1^g, b_2^g, and b_3^g, a prior of N(0, 0.35) is used. Input data are standardized to the standard normal scale to ensure priors are sensible for all dietary factors and to increase computational stability.

2.3. Covariate effects

There were two survey-level covariate effects included in the model to explain potential bias from a survey: survey type and food definition. There were four main types of diet surveys included as covariates in the model: short-term recalls (single or multiple); food frequency questionnaires (FFQs); household budget/intake surveys; and DHS (Demographic Health Survey) questionnaires. Only the recall is considered the "gold standard" with regards to estimating the mean unbiasedly. Likewise, not all surveys use the optimal definition for a dietary factor. For example, in the case of fruits, most surveys define fruits as "all fruits". However, some surveys will only measure a sub-optimal metric, such as fruits and fruit juices combined. Currently, we combine all sub-optimal metrics into one category. Of note, this was not the case for sugar-sweetened beverages (SSBs), as all surveys report using the optimal definition (i.e., "total sugar-sweetened beverage intake, defined as any beverage with added sugars having ≥50 kcal per 8 oz (236.5g) serving, including commercial or homemade beverages, soft drinks, energy drinks, fruit drinks, punch, lemonade, and frescas; excluding 100% fruit and vegetable juices and non-caloric artificially-sweetened drinks").We also included country-year specific predictors in the model (e.g., food availability FAO food balance sheets or Global Expanded Nutrient Supply (GENuS) model). The model assumed the relationship of these covariates to $f(y_i)$ was linear, and that the relationships were independent of location (not super-region dependent, or country dependent) and year. Mathematically, this portion of the model can be described as follows:

$$X_i\beta = X_i^{survey\ characteristics}\beta_s + X_{j[i]}^{country-year\ predictions}\beta_c$$

where,

$X_i\beta$ ⇐ study + country level covariate effects
$X_i^{survey\ characteristics}\beta_s$ ⇐ study characteristics for study i + world region level covariate effects
$X_{j[i]}^{country-year\ predictions}\beta_c$ ⇐ country-year predictors for study i in country j + country level covariate effects

For survey level-covariates, we used a prior of Normal(0, 0.35). The prior for βc parameters depended on the dietary factor. For many dietary factors, we only used 1 or 2 country-level covariates, all from FAO. For these variables, we had a very strong prior belief that they should be strongly correlated with our outcome variable of interest (e.g., log(sugar and sweeteners availability from FAO) should be strongly positively correlated with log(SSBs intake)). In these cases, we used a highly informative prior of N(1, 0.1). For other dietary factors, either no such variable existed, or other country-year level predictors were also included and do not warrant such a high degree of certainty in a strong relationship. In these cases, we used a much weaker prior of N(0, 0.5).

2.4 Age trend

For many surveys, intake was not linearly associated with age. We modelled age using restricted cubic splines with 4 knots at $k1$, $k2$, $k3$, $k4$, corresponding to ages 5, 20, 50 and 65, respectively, after standardization:

$$\gamma_{j[i]}(z_h) = \gamma_{1j[i]}z_h + \gamma_{2j[i]}S_1 + \gamma_{3j[i]}S_2$$

where,

$$S_1 = (z_h - k_1)^3 - \frac{k_4 - k_1}{k_4 - k_3}(z_h - k_3)^3 + \frac{k_3 - k_1}{k_4 - k_3}(z_h - k_4)^3$$

$$S_2 = (z_h - k_2)^3 - \frac{k_4 - k_2}{k_4 - k_3}(z_h - k_3)^3 + \frac{k_3 - k_2}{k_4 - k_3}(z_h - k_4)^3$$

As with the urban and education effect parameters, we used 2 levels of hierarchy for the age-trend:

$$\gamma_{1j[i]} = \gamma_{1k[j]}^s + \gamma_1^g$$
$$\gamma_{2j[i]} = \gamma_{2k[j]}^s + \gamma_2^g$$
$$\gamma_{3j[i]} = \gamma_{3k[j]}^s + \gamma_3^g$$

$$\gamma_{1k}^s \sim N(0, \kappa_{1\gamma}^s), \gamma_{2k}^s \sim N(0, \kappa_{2\gamma}^s), \gamma_{3k}^s \sim N(0, \kappa_{3\gamma}^s)$$

Weakly informative priors are used for the hyper-parameters: half-Normal (0, 0.5) for the κ parameters and Normal (0, 0.35) for γ^g parameters.

2.5 Overdispersion

An additional variance component was added to each study to allow the model to account for non-sampling variation due to survey-level error (from imperfect study design and quality). This additional variance component was modeled in such a way to reflect our expectation that surveys that are less likely to represent the true mean (but not necessarily biased) were more variable. Sources of this non-sampling variation accounted for included surveys not being nationally representative, surveys not being stratified by sex, urban/rural or education, and surveys that used large age groupings (greater than10 years). We also added an additional constraint to ensure local surveys were considered more variable than regional surveys.

Thus,

$$\tau_i^2 = exp(\phi_{intercept} + \phi_{regional}I(X_i^{rep} = regional) + \phi_{local}I(X_i^{rep} = local)$$
$$+ \phi_{AgeRange}I(X_i^{AgeRange} > 10)$$
$$+ \phi_{sex}I(X_i^{sex} = both) + \phi_{urban/rural}I((X_i^{urban/rural} = both)\ or\ (X_i^{educ} = all))$$

with the constraints $\phi^2_{regional} < \phi^2_{local}$, and all $\phi > 0$ except $\phi_{intercept}$. We use a prior of Normal (-2.5, 1) for $\phi_{intercept}$ to reflect our a priori belief than an "ideal" survey that is both fully stratified and nationally representative should have minimal overdispersion. For all other ϕ parameters, we used a prior of Normal (0, 0.5).

3 Computation

We fit each model using STAN[5,6] through Rstan,[7] using the No-U-turn sampler (NUTS),[8] a variant of Hamiltonian Monte Carlo.[9] We use 4 chains of 2000 iterations each, treating the first 1000 iterations of each chain as warm up, for a total of 4000 Monte Carlo iterations to define our posterior distributions.

4 Estimates

The model described above was ultimately used to provide posterior distributions of mean intake for each dietary factor by country-year and subgroup. Note that the model specified $g(y_{h,i[j]})$ of subgroup h in survey i from country j as a linear combination of model parameters and survey-year-subgroup specific information:

$$a_j + b_{1j} sex_{h,i} + b_{2s} u_{h,i} + b_{3s} educ_{h,i} + \gamma_s(z_{h,i}) + X_i \beta$$

where we have posterior distributions for model parameters a_j, b_j, b_{2s}, b_{3s}, γ_s, and β. To obtain a posterior distribution for subgroup h in country j, we calculate for each draw of our posterior distribution:

$$\mu_{hj} = g^{-1}(a_j + b_{1j} sex_{h,j} + b_{2s} u_{h,j} + b_{3s} educ_{h,j} + \gamma_s(z_{h,j}) + X_j^{country-year\ predictors} \beta_c)$$

Because we are interested in country-specific means, we did not use survey specific parameters in our estimates. For countries with no survey data, we did not have posterior distribution for a_j. To get the posterior distributions for these countries in such a way that accounts for the variation of mean intake between countries within a region, we report the distribution of $a_j^*$and b_{1j}^*, where $a_j^* \sim N(a_{k[j]}, \kappa_a^c)$ and $b_{1j}^* \sim N(b_{1k[j]}, \kappa_{1b}^c)$. Here, $a_{k[j]}$ and $b_{1k[j]}$ are world region-level intercepts and sex effects corresponding to country j, and κ_a^c and κ_{1b}^c are the between-country variances for intercept and sex effects, respectively. In other words, each posterior draw for the world region-level parameter and it's corresponding between-country variance parameter generates a unique normal distribution for that draw, and we take a one sample draw from each of these distributions to generate the posterior distribution of that parameter for an unknown country in that region. Note that the uncertainty around the world region level parameter and between country variance propagate into the posterior distribution for the mean.
For some dietary factors, there were entire world regions with no data. For those world regions, posterior distributions for b_{2s}, b_{3s}, and γ_s were obtained in a similar way, generating a normal distribution for each draw from the global level parameter and between region variance parameter and sampling from that. For a_j and b_{1j}, we needed to account for between-world region variance and the between-country variance. Therefore, taking the intercept as an example, for each posterior draw, we sampled from $N(a^g, \kappa_a^c + \kappa_a^s)$. Note that this is equivalent to drawing a sample region mean from $N(a^g, \kappa_a^s)$ then using that sample as mean and κ_a^c as variance to form a normal distribution to sample country mean from.

5 Varying slopes modeling structure

- Our extensive work to identify surveys and model intakes led to recognition and the finding that, for certain dietary factors, the available global data and model were insufficient to accurately model differences in intakes by jointly stratified by country, age, sex, education level, and urban/rural status while also modeling differences in intakes over time.
- For countries without multiple comparable dietary surveys over time (the great majority of global nations), trends over time are largely determined by the strength of the relationship between the best available covariates (often variables from FAO food balance sheets or associated GENuS variables) and the raw survey data. For certain dietary factors, this relationship was sufficiently robust to allow modeling of all joint demographic strata and time trends. By reviewing extensive time trends plots for individual dietary factors and nations, dietary factors with a model beta coefficient>=0.4 with their corresponding FAO/GENuS covariate were identified as having a reasonable statistical relationship to capture both all demographic strata differences and time trends. For others (FAO/GENuS beta coefficient<0.4), time trends were modeled using a second, separate Bayesian model.
- This second Bayesian model assessed the country-specific associations over time of the survey data for each dietary factor with its corresponding FAO/GENuS covariate. The model incorporated country-level intercepts and slopes, along with their correlation that is estimated across countries, to provide more sensitivity and specificity for estimating country-specific associations from dietary data to generate country-specific time trends. Input data were the same stratified survey data as for the GDD Core model and including dietary assessment method as a covariate. This time component model did not separately estimate differences by age, sex, education, or urban/rural status, but focused on the relationships with FAO/GENuS over time. In sensitivity analyses, age and sex were included as main effects (not varying by country or region) but were found to not qualitatively alter the parameter estimates for the relationship of a country's dietary intake data with its FAO/GENuS data. Thus, including these demographics did not largely affect the time-varying estimates. This model is commonly referred as a varying slopes model structure and leverages two-dimensional partial pooling between intercepts and slopes to regularize all parameters and minimize overfitting risk[10-12]. Estimates with the varying slopes model take into account a country-specific intercept and slope when the country has dietary factor data and use the global intercept and slope for countries where data are not available. Time effects were predicted separately for each year including 1990, 1995, 2000, 2005, 2010, 2015, and 2018.
- For each country and dietary factor, the country-specific time-trend central estimates from the varying slopes models were used to generate a country-year specific adjustment scaling factor, one for each year of 1990, 1995, 2000, 2005, 2010, 2015, and 2018, compared to the reference of one of these years as determined by the median year of that country's survey data (or 2005 if no country data). This scaling factor, determined by taking the ratio of the predicted dietary intake for that year as compared to the reference year, was multiplied by the country-year posterior estimates from the fully stratified, Core GDD model to determine a time-adjusted final estimate for each stratum.
- To be conservative, this varying slopes adjustment (scaling factor) was only used for dietary factors and countries meeting all of the following criteria: at the model level, (a) FAO/GENuS beta coefficient<0.4 in the Core GDD model; and (b) availability of a closely corresponding FAO/GENuS covariate (e.g., dietary survey vitamin A intake vs. GENuS vitamin A); and at the country-level, (c) identification of a positive relationship (coefficient or slope) between the national survey data and FAO/GENuS covariate in the varying slopes model; and (d) to minimize implausible results at the country level, no more than a 3-fold difference between the ratio of the country's range of predicted intake between 1990-2018 divided by the ratio of the country's range of FAO/GENuS values over that same time period.
- Among 53 evaluated dietary factors in the GDD, 29 were modeled and incorporated time adjustment using this Bayesian varying slopes model. The other dietary factors were not because (in order of criteria applied) 11 did not have any closely corresponding FAO/GENuS variable (e.g., dietary iodine), 8 had an FAO/GENuS beta coefficient in Core GDD Core Model of at least 0.4, and 4 were unable to complete sampling for the varying-slopes model (i.e., the MCMC chains did not finalize, independent of parameterization). One additional dietary factor, vitamin B9, with a borderline FAO/GENuS beta (0.34) was also not further scaled based on adequate qualitative characteristics of the observed time trends in the GDD Core Model.

A measurement error, varying slopes model that accounts for dietary assessment method, using standardized log-intakes for all dietary factors except those reported in percent energy:

$Y_{obs,i} \sim Normal(Y_{true,i}\ , DE_{SE,i})$ [distribution for observed intake, Y_{obs}, including measurement error associated with the stratum estimate, $DE_{SE,i}$]

$Y_{true,i} \sim Normal(\mu_i\ , \sigma)$ [distribution for true strata intake Y]

$\mu_i = \alpha_{country[i]} + \beta_{country[i]} * FAO + M_{method[i]}$ [linear equation for the average intake; Each country receives its own intercept and slope while also accounting for dietary assessment method]

$\begin{bmatrix} \alpha_{country} \\ \beta_{country} \end{bmatrix} \sim MVNormal\left(\begin{bmatrix} \alpha \\ \beta \end{bmatrix}, S\right)$ [population of varying effects]

$S = \begin{pmatrix} \sigma_\alpha & 0 \\ 0 & \sigma_\beta \end{pmatrix} R \begin{pmatrix} \sigma_\alpha & 0 \\ 0 & \sigma_\beta \end{pmatrix}$ [construct covariance matrix]

With hyperpriors that define the adaptive varying effects and effects for dietary assessment:

$\alpha \sim Normal(0\ , 1)$ [prior for average intercept]

$\beta \sim Normal(1\ , 0.11)$ [prior for average slope]

$M[method] \sim Normal(0\ , 0.2)$ [prior for method effect]

$\sigma \sim Halfnormal\ (0\ ,0.5)$ [prior for stddev within countries]

$\sigma_\alpha \sim Halfnormal\ (0\ ,0.5)$ [prior for stddev among intercepts]

$\sigma_\beta \sim Halfnormal\ (0\ ,0.5)$ [prior for stddev among slopes]

$R \sim LKJcorr\ (2)$ [prior for correlation matrix]

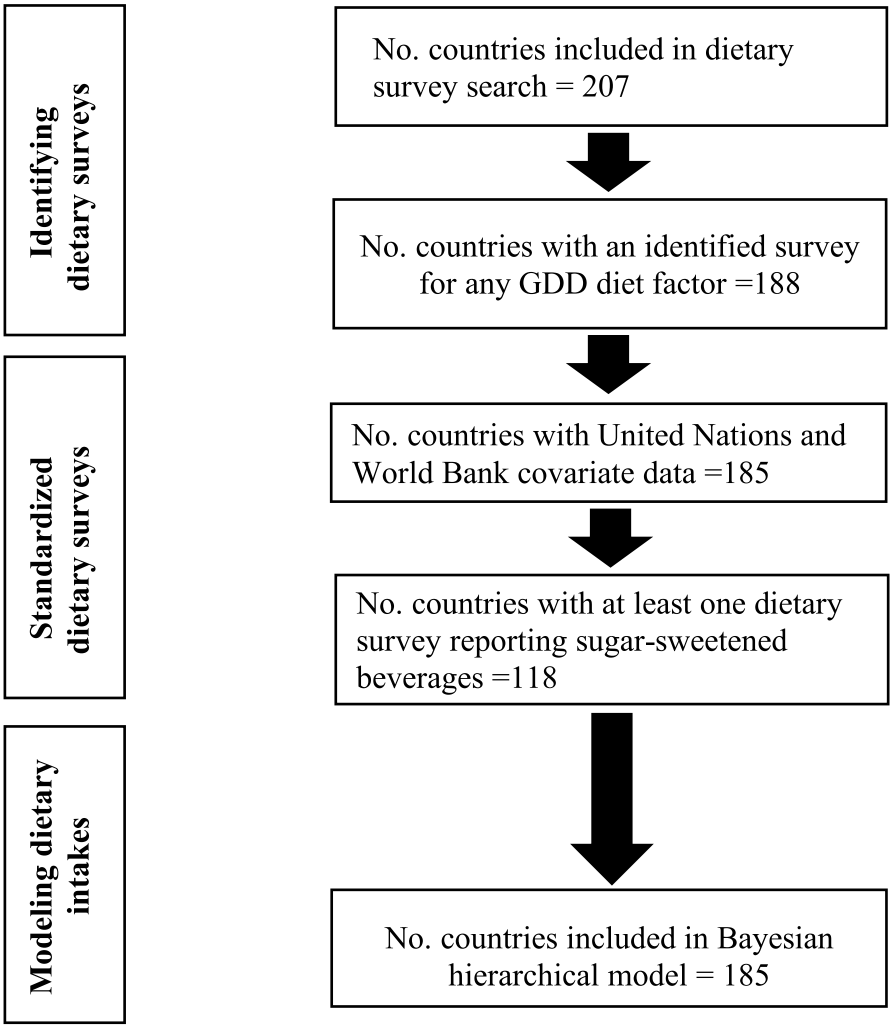

Supplementary Figure 1. **Flowchart of number of countries with dietary surveys identified, standardized, and included in the Bayesian hierarchical model.** Any GDD diet factor: fruit; non-starchy vegetables; potatoes; other starchy vegetables; beans/legumes; nuts/seeds; refined grains; whole grains; processed meats; unprocessed red meats; seafood; eggs; cheese; yogurt; sugar- sweetened beverages; fruit juices; coffee; tea; reduced fat milk; whole fat milk; total milk; energy; carbohydrate, total protein; animal protein; plant protein; saturated fat; monounsaturated fat; omega-6 fat; seafood omega-3 fat; plant omega-3 fat; dietary cholesterol; dietary fiber; added sugar; calcium; sodium; iodine; iron; magnesium; potassium; selenium; vitamin A with supplement; vitamin A without supplement; vitamin B1; vitamin B2; vitamin B3; vitamin B6; vitamin B9; vitamin C; vitamin D; vitamin E or zinc.

Supplementary Table 3. Countries by world region (superregion) in GDD 2018.

Central or Eastern Europe and Central Asia[†]		High-Income Countries	Latin America and Caribbean		Middle East and North Africa	South Asia[§]	Southeast and East Asia	Sub-Saharan Africa	
n=29		*n=24*	*n=32*		*n=20*	*n=8*	*n=24*	*n=48*	
Albania	Slovenia	Australia	Antigua and Barbuda	Panama	Algeria	Afghanistan	Brunei	Angola	Liberia
Armenia	Tajikistan	Austria	Argentina	Paraguay	Bahrain	Bangladesh	Cambodia	Benin	Madagascar
Azerbaijan	Turkmenistan	Belgium	Bahamas, The	Peru	Egypt, Arab Rep.	Bhutan	China	Botswana	Malawi
Belarus	Ukraine	Canada	Barbados	St. Lucia	Iran, Islamic Rep.	India	Fiji	Burkina Faso	Mali
Bosnia and Herzegovina	Uzbekistan	Cyprus	Belize	St. Vincent and the Grenadines	Iraq	Maldives	Indonesia	Burundi	Mauritania
Bulgaria		Denmark	Bolivia	Suriname	Israel	Nepal	Japan	Cameroon	Mauritius
Croatia		Finland	Brazil	Trinidad and Tobago	Jordan	Pakistan	Kiribati	Cape Verde	Mozambique
Czech Republic		France	Chile	Uruguay	Kuwait	Sri Lanka	Korea, Rep.	Central African Republic	Namibia
Estonia		Germany	Colombia	Venezuela	Lebanon		Lao PDR	Chad	Niger
Georgia		Greece	Costa Rica		Libya		Malaysia	Comoros	Nigeria
Hungary		Iceland	Cuba		Morocco		Marshall Islands	Congo, Dem. Rep.	Rwanda
Kazakhstan		Ireland	Dominica		Oman		Micronesia, Fed. Sts.	Congo, Rep.	Sao Tome and Principe
Kyrgyz Republic		Italy	Dominican Republic		Palestine		Myanmar	Cote d'Ivoire	Senegal
Latvia		Luxembourg	Ecuador		Qatar		Papua New Guinea	Djibouti	Seychelles
Lithuania		Malta	El Salvador		Saudi Arabia		Philippines	Equatorial Guinea	Sierra Leone
Macedonia, FYR		Netherlands	Grenada		Syrian Arab Republic		Samoa	Eritrea	South Africa
Moldova		New Zealand	Guatemala		Tunisia		Singapore	Ethiopia (excludes Eritrea)	South Sudan
Mongolia		Norway	Guyana		Turkey		Solomon Islands	Gabon	Sudan
Montenegro		Portugal	Haiti		United Arab Emirates		Taiwan	Gambia, The	Swaziland
Poland		Spain	Honduras		Yemen, Rep.		Thailand	Ghana	Tanzania
Romania		Sweden	Jamaica				Timor-Leste	Guinea	Togo
Russian Federation		Switzerland	Mexico				Tonga	Guinea-Bissau	Uganda
Serbia		United Kingdom	Nicaragua				Vanuatu	Kenya	Zambia
Slovak Republic		United States					Vietnam	Lesotho	Zimbabwe

[†]Referred to as Former Soviet Union in previous Global Dietary Database reports. [§]Referred to as Asia in previous Global Dietary Database reports.

Supplementary Table 4. Characteristics of global data sources of sugar-sweetened beverage intakes.

	World	Central or Eastern Europe and Central Asia†	High-Income Countries	Latin America and Caribbean	Middle East and North Africa	South Asia†	Southeast and East Asia	Sub-Saharan Africa
Number of surveys (% nationally or sub-nationally representative)	451 (94.2%)*	89 (98.9%)	210 (97.6%)	40 (92.5%)	39 (84.6%)	9 (77.8%)	42 (71.4%)	22 (81.8%)
Number of countries represented	118	18	24	22	16	5	18	15
Time period of surveys	1980-2018	1985-2015	1980-2018	1993-2015	1990-2015	1993-2012	1990-2017	1995-2016
Total sample size of surveyed subjects§	2,941,704	390,246	1,289,722	300,342	225,687	81,630	605,044	49,033
By sex								
Female	1,302,424 (44.3%)	154,851 (39.7%)	451,638 (35.0%)	179,558 (59.8%)	119,603 (53.0%)	41,930 (51.4%)	329,007 (54.4%)	25,837 (52.7%)
Male	1,639,280 (55.7%)	235,395 (60.3%)	838,084 (65.0%)	120,784 (40.2%)	106,084 (47.0%)	39,700 (48.6%)	276,037 (45.6%)	23,196(47.3%)
By area of residence								
Urban	2,058,260 (70.0%)	246,967 (63.3%)	974,386 (75.6%)	220,178 (73.3%)	152,482 (67.6%)	23,804 (29.2%)	426,267 (70.5%)	14,176 (28.9%)
Rural	883,444 (30.0%)	143,279 (36.7%)	315,336 (24.4%)	80,164 (26.7)	73,205 (32.4%)	57,826 (70.8%)	178,777 (29.5%)	34,857 (71.1%)
By education level								
Low education	470,899 (16.0%)	20,509 (5.3%)	130,957 (10.2%)	112,565 (37.5%)	71,817 (31.8%)	35,838 (43.9%)	70,807 (11.7%)	28,405 (57.9%)
Medium education	1,106,243 (37.6%)	131,621 (33.7%)	530,353 (41.1%)	115,886 (38.6%)	68,382 (30.3%)	26,462 (32.4%)	219,211 (36.2%)	14,329 (29.2%)
High education	1,364,562 (46.4%)	238,116 (61.0%)	628,412 (48.7%)	71,891 (23.9%)	85,488 (37.9%)	19,330 (23.7%)	315,026 (52.1%)	6,299 (12.8%)
By adult/youth								
Adults (18+ years)	1,564,936 (53.2%)	119,355 (30.6%)	652,460 (50.6%)	106,863 (35.6%)	110,400 (48.9%)	40,819 (50.0%)	530,735 (87.7%)	4,304 (8.8%)
Children/adolescent (<18 years)	1,376,768 (46.8%)	270,891 (69.4%)	637,262 (49.4%)	193,479 (64.4%)	115,287 (51.1%)	40,811 (50.0%)	74,309 (12.3%)	44,729 (91.2%)
World population represented in 2018ƒ	6,596,636 (87.1%)	310,208 (74.6%)	817,099 (100.0%)	551,485 (86.5%)	476,147 (89.9%)	1,747,992 (96.3%)	2,231,431 (97.2%)	462,274 (43.4%)
Number of surveys by characteristic¥								
By adult/youth								
Adults	192 (42.6%)	29 (32.6%)	93 (44.3%)	13 (32.5%)	12 (30.8%)	4 (44.4%)	31 (73.8%)	10 (54.5%)
Children/adolescents	323 (71.6%)	65 (73.0%)	143 (68.1%)	35 (87.5%)	30 (76.9%)	7 (77.8%)	27 (64.3%)	16 (72.7%)
By area of residence								
Both urban and rural	278 (61.6%)	62 (69.7%)	115 (54.8%)	28 (70.0%)	28 (71.8%)	4 (44.4%)	27 (64.3%)	14 (63.3%)
Only urban	13 (2.9%)	0 (0%)	1 (0.5%)	3 (7.5%)	6 (15.4%)	1 (11.1%)	0 (0%)	2 (9.0%)
Only rural	7 (1.6%)	0 (0%)	0 (0%)	0 (0%)	0 (0%)	3 (33.3%)	1 (2.4%)	3 (13.6%)
Not reported	153 (33.9%)	27 (30.3%)	94 (44.8%)	9 (22.5%)	5 (12.8%)	1 (11.1%)	14 (33.3%)	3 (13.6%)
By dietary assessment method								
FFQ	273 (60.5%)	58 (65.2%)	115 (54.8%)	27 (67.5%)	27 (69.2%)	5 (55.6%)	29 (69.0%)	12 (54.5%)
24hr recall	109 (24.2%)	14 (15.7%)	58 (27.7%)	8 (20.0%)	7 (17.9%)	2 (22.2%)	12 (28.5%)	8 (36.3%)
Household budget survey	54 (12.0%)	17 (19.1%)	37 (17.6%)	0 (0%)	0 (0%)	0 (0%)	0 (0%)	0 (0%)
DHS	15 (3.3%)	0 (0%)	0 (0%)	5 (12.5%)	5 (12.8%)	2 (22.2%)	1 (2.4%)	2 (9.1%)

*Of the 451 surveys reporting data on sugar-sweetened beverages, 46.5% of surveys were in High-Income Countries, followed by Central/Eastern Europe and Central Asia (19.7%), Southeast and East Asia (9.3%), Latin American and the Caribbean (8.9%), Middle East and Northern Africa (8.6%), Sub-Saharan Africa (4.9%), and South Asia (2.2%).

§ The total of subjects sampled can correspond to multiple years, thus in some cases this number is larger than the world population represented in 2018 shown in a few rows below.

ƒ The values correspond to the total population of the countries with sugar-sweetened beverage intake data and the percentage in relation to the total population of all countries within that region.

¥ The values represent the absolute number of surveys and the percentage in relation to the total number of surveys within that region.

† In prior GDD reports, the region Central or Eastern Europe and Central Asia was referred as Former Soviet Union, and Southeast and East Asia was referred as Asia.

Supplementary Table 5. Number of dietary surveys for SSB intake globally, regionally, and in the 25 most populous countries by time period.

	1980-1984	1985-1989	1990-1994	1995-1999	2000-2004	2005-2009	2010-2014	2015-2018
World	3	17	52	75	82	88	116	18
World Region								
Central Eastern Europe and Central Asia†	0	2	10	11	29	18	18	1
High-Income Countries	3	15	39	49	36	32	29	7
Latin America and Caribbean	0	0	0	4	2	16	17	1
Mid. East and North Africa	0	0	2	3	6	8	18	2
South Asia§	0	0	1	1	1	2	4	0
Southeast and East Asia	0	0	0	6	8	9	15	4
Sub-Saharan Africa	0	0	0	1	0	3	15	3
Country								
China	0	0	0	0	1	0	0	0
India	0	0	0	0	0	0	3	0
United States	0	1	3	3	4	3	4	2
Indonesia	0	0	0	0	0	1	0	0
Brazil	0	0	0	0	0	2	1	0
Pakistan	0	0	0	0	0	1	0	0
Russia	0	0	1	1	1	1	1	0
Japan	0	0	0	5	3	0	0	0
Bangladesh	0	0	1	1	0	0	1	0
Mexico	0	0	0	0	0	2	1	0
Germany	0	1	2	4	1	3	1	0
Vietnam	0	0	0	0	0	1	1	0
Philippines	0	0	0	0	1	1	1	0
Egypt	0	0	0	1	1	1	1	0
Iran	0	0	0	1	0	2	2	2
Turkey	0	0	0	0	1	2	1	0
Thailand	0	0	0	0	1	0	0	0
Ethiopia	0	0	0	0	0	2	1	0
United Kingdom	0	2	5	6	2	1	1	0
Italy	1	0	2	2	1	2	2	1
France	0	0	1	2	1	2	1	0
Korea	0	0	0	0	1	4	5	1
Spain	1	0	2	1	1	1	1	0
DR Congo	0	0	0	0	0	0	0	1

*Countries are ordered top to bottom from most to least populous in 2018 based on 2018 adult (20+ years) population data . †Referred to as Former Soviet Union in previous Global Dietary Database reports. §Referred to as Asia in previous Global Dietary Database reports.

Supplementary Table 6. National mean (95% UI) sugar-sweetened beverage intakes (8 oz servings/week) in adults (20+ years) by sex, age, education, and area of residence in the 25 most populous countries in 2018.

	Sex		Age category			Education			Area of residence	
	Female	Male	20-39 years	40-59 years	60+ years	≤6 years	>6-12 years	>12 years	Rural	Urban
Country	*mean (95% UI)*	*mean (95% UI)*	*mean (95% UI)*	*mean (95% UI)*	*mean (95% UI)*	*mean (95% UI)*	*mean (95% UI)*	*mean (95% UI)*	*mean (95% UI)*	*mean (95% UI)*
China	0.2 (0.2-0.3)	0.2 (0.2-0.3)	0.4 (0.3-0.4)	0.2 (0.1-0.2)	0.1 (0.1-0.1)	0.2 (0.2-0.3)	0.2 (0.2-0.3)	0.2 (0.2-0.3)	0.2 (0.2-0.3)	0.2 (0.2-0.3)
India	0.2 (0.1-0.3)	0.2 (0.1-0.4)	0.2 (0.1-0.4)	0.2 (0.1-0.3)	0.1 (0.1-0.2)	0.1 (0.1-0.2)	0.2 (0.1-0.4)	0.3 (0.2-0.5)	0.1 (0.1-0.1)	0.4 (0.2-0.7)
United States	4.3 (4.1-4.6)	5.4 (5.1-5.7)	7.3 (6.9-7.7)	4.3 (4.1-4.6)	2.5 (2.3-2.6)	6.2 (5.8-6.8)	6.1 (5.7-6.6)	4.5 (4.2-4.8)	5.0 (4.8-5.3)	4.8 (4.5-5.1)
Indonesia	0.9 (0.6-1.2)	1.0 (0.7-1.3)	1.3 (1.0-1.7)	0.6 (0.5-0.8)	0.4 (0.3-0.5)	0.8 (0.6-1.1)	1.0 (0.8-1.3)	1.0 (0.8-1.3)	0.9 (0.7-1.2)	0.9 (0.7-1.2)
Brazil	4.1 (3.6-4.8)	4.6 (4.0-5.4)	5.5 (4.8-6.2)	3.8 (3.3-4.3)	2.8 (2.3-3.2)	4.2 (3.6-4.9)	4.5 (3.9-5.2)	4.6 (3.9-5.4)	3.9 (3.4-4.6)	4.5 (3.9-5.0)
Pakistan	4.2 (2.5-7.2)	4.2 (2.6-7.1)	4.8 (3.0-7.8)	3.6 (2.1-6.4)	2.8 (1.5-5.1)	3.1 (1.8-5.4)	5.7 (3.4-9.9)	7.6 (4.4-13.6)	1.8 (1.2-2.9)	8.4 (4.9-14.8)
Russia	2.1 (1.7-2.6)	2.6 (2.1-3.2)	3.5 (2.9-4.3)	1.8 (1.5-2.3)	1.3 (1.0-1.6)	2.2 (1.7-3.0)	2.5 (2.0-3.1)	2.3 (1.8-2.7)	2.5 (2.0-3.0)	2.3 (1.8-2.7)
Japan	1.7 (1.4-2.0)	2.1 (1.8-2.5)	3.6 (3.1-4.1)	1.7 (1.5-2.0)	1.0 (0.8-1.1)	1.6 (1.3-1.9)	1.9 (1.7-2.2)	1.9 (1.7-2.2)	1.8 (1.6-2.1)	1.9 (1.6-2.2)
Bangladesh	0.2 (0.1-0.3)	0.2 (0.2-0.4)	0.3 (0.2-0.4)	0.2 (0.1-0.3)	0.2 (0.1-0.3)	0.2 (0.1-0.3)	0.3 (0.2-0.5)	0.4 (0.2-0.7)	0.1 (0.1-0.1)	0.5 (0.3-0.8)
Nigeria	4.7 (3.0-7.3)	5.0 (3.2-7.7)	5.3 (3.5-8.2)	4.4 (2.7-6.9)	3.1 (1.8-5.2)	4.1 (2.5-6.5)	6.9 (4.4-10.5)	7.6 (4.7-12.0)	3.8 (2.5-5.8)	6.0 (3.7-9.3)
Mexico	8.3 (7.4-9.4)	9.6 (8.6-10.7)	11.0 (10.0-12.2)	7.6 (6.9-8.5)	5.5 (4.8-6.3)	8.5 (7.6-9.6)	9.1 (8.2-10.3)	9.2 (8.0-10.7)	8.0 (7.2-8.9)	9.2 (8.3-10.2)
Germany	2.3 (2.0-2.6)	3.2 (2.8-3.6)	4.4 (4.0-4.9)	2.6 (2.3-2.9)	1.5 (1.3-1.6)	3.1 (2.7-3.5)	3.0 (2.7-3.4)	2.2 (2.0-2.5)	2.8 (2.5-3.2)	2.7 (2.4-3.0)
Vietnam	1.5 (1.2-2.0)	1.6 (1.2-2.1)	2.3 (1.8-2.9)	1.1 (0.8-1.4)	0.6 (0.5-0.8)	1.4 (1.1-1.8)	1.7 (1.3-2.2)	1.7 (1.3-2.2)	1.6 (1.2-2.0)	1.6 (1.3-2.1)
Philippines	3.4 (2.9-4.0)	3.0 (2.4-3.8)	4.5 (3.9-5.2)	2.0 (1.8-2.4)	1.3 (1.1-1.5)	2.8 (2.4-3.3)	3.4 (2.9-3.9)	3.4 (2.9-3.9)	3.2 (2.7-3.7)	3.3 (2.9-3.8)
Egypt	3.0 (2.5-3.7)	2.6 (2.0-3.2)	3.4 (2.8-4.2)	2.1 (1.7-2.6)	1.9 (1.5-2.5)	2.8 (2.3-3.5)	3.0 (2.4-3.8)	2.5 (2.0-3.1)	3.0 (2.4-3.7)	2.6 (2.1-3.2)
Iran	2.6 (2.3-3.0)	2.8 (2.4-3.2)	3.3 (2.9-3.7)	2.1 (1.8-2.4)	1.9 (1.6-2.3)	2.7 (2.4-3.1)	2.9 (2.6-3.4)	2.4 (2.1-2.8)	3.0 (2.6-3.5)	2.6 (2.3-2.9)
Turkey	2.7 (2.0-3.6)	2.8 (2.1-3.8)	3.5 (2.7-4.6)	2.2 (1.6-2.8)	2.0 (1.5-2.7)	2.7 (2.1-3.6)	2.9 (2.2-3.8)	2.4 (1.8-3.2)	3.0 (2.3-4.1)	2.6 (2.0-3.5)
Thailand	4.3 (2.1-9.2)	4.8 (2.4-9.7)	7.5 (3.7-15.3)	3.4 (1.7-7.1)	2.1 (1.0-4.2)	4.3 (2.1-8.7)	5.1 (2.5-10.7)	5.1 (2.5-10.6)	4.5 (2.2-9.3)	4.7 (2.3-9.6)
Ethiopia	6.9 (5.3-8.9)	7.3 (5.6-9.5)	7.8 (6.2-9.9)	6.4 (5.0-8.2)	4.5 (3.0-6.7)	5.8 (4.5-7.5)	9.8 (7.5-12.7)	10.9 (7.9-15.2)	6.4 (5.0-8.1)	10.1 (7.8-13.1)
United Kingdom	4.3 (3.8-4.8)	4.5 (4.0-5.1)	6.7 (6.1-7.4)	4.0 (3.6-4.4)	2.3 (2.0-2.5)	4.7 (4.3-5.3)	4.6 (4.2-5.1)	3.4 (3.1-3.8)	4.6 (4.1-5.1)	4.3 (4.0-4.8)
Italy	1.4 (1.2-1.5)	1.7 (1.5-1.9)	2.5 (2.2-2.8)	1.5 (1.3-1.7)	0.8 (0.7-0.9)	1.7 (1.5-1.9)	1.6 (1.5-1.8)	1.2 (1.1-1.3)	1.6 (1.4-1.8)	1.5 (1.3-1.7)
France	2.3 (2.0-2.6)	3.3 (2.9-3.8)	4.4 (4.0-4.9)	2.7 (2.4-3.0)	1.5 (1.3-1.6)	3.1 (2.8-3.5)	3.1 (2.8-3.4)	2.3 (2.0-2.5)	2.9 (2.6-3.2)	2.8 (2.5-3.1)
Korea	0.7 (0.7-0.8)	1.1 (1.0-1.2)	1.5 (1.4-1.6)	0.7 (0.6-0.8)	0.4 (0.4-0.5)	0.8 (0.7-0.8)	0.9 (0.8-1.0)	0.9 (0.8-1.0)	0.9 (0.8-0.9)	0.9 (0.8-1.0)
Spain	2.9 (2.4-3.6)	3.3 (2.7-4.1)	4.8 (4.1-5.8)	3.0 (2.5-3.6)	1.6 (1.4-2.0)	3.3 (2.8-4.0)	3.3 (2.7-3.9)	2.4 (2.0-2.8)	3.2 (2.7-3.9)	3.1 (2.6-3.7)
DR Congo	1.8 (0.7-5.1)	2.1 (0.7-5.7)	2.2 (0.8-5.9)	1.8 (0.6-4.9)	1.2 (0.4-3.5)	1.6 (0.6-4.6)	2.8 (1.0-7.5)	3.1 (1.1-8.2)	1.6 (0.6-4.3)	2.5 (0.9-6.8)

*Data are mean intakes (95% uncertainty interval) in 8 oz servings per week. Standardized serving size used for this analysis: 8 oz serving = 248 grams. Countries are ordered top to bottom from most to least populous based on 2018 adult (20+ years) population data. Source data are provided as Source Data file 8.

Supplementary Table 7. Absolute difference in sugar-sweetened beverage intakes (8 oz serving/week) by sex, education level, and area of residence globally and regionally in adults (20+years) across 185 countries in 2018.

	World	Central or Eastern Europe and Central Asia†	High-Income Countries	Latin America and Caribbean	Middle East and North Africa	South Asia§	Southeast and East Asia	Sub-Saharan Africa
	mean (95% UI)	*mean (95% UI)*	*mean (95% UI)*	*mean (95% UI)*	*mean (95% UI)*	*mean (95% UI)*	*mean (95% UI)*	*mean (95% UI)*
Males vs. Females								
Overall	0.19 (0.05,0.33)	0.41 (0.17,0.68)	0.80 (0.63,0.96)	0.76 (0.01,1.50)	0.09 (-0.46,0.58)	0.04 (-0.12,0.21)	0.04 (-0.06,0.14)	0.35 (-0.48,1.16)
Age (years)								
20-39	0.16 (-0.04,0.37)	0.38 (-0.02,0.80)	1.02 (0.76,1.28)	0.78 (-0.17,1.71)	0.02 (-0.70,0.64)	0.03 (-0.17,0.24)	0.03 (-0.14,0.19)	0.31 (-0.68,1.29)
39-59	0.11 (-0.01,0.23)	0.20 (0.00,0.41)	0.59 (0.44,0.74)	0.51 (-0.17,1.14)	0.10 (-0.32,0.53)	0.03 (-0.10,0.18)	0.02 (-0.06,0.09)	0.29 (-0.56,1.13)
60+	0.11 (0.04,0.18)	0.20 (0.06,0.35)	0.39 (0.31,0.48)	0.47 (-0.02,0.96)	0.04 (-0.31,0.37)	0.03 (-0.06,0.14)	0.01 (-0.04,0.06)	0.17 (-0.46,0.86)
Education (years)								
0-6	0.10 (-0.08,0.27)	0.40 (0.13,0.73)	0.81 (0.59,1.03)	0.68 (-0.05,1.38)	0.05 (-0.62,0.63)	0.03 (-0.11,0.18)	0.04 (-0.10,0.16)	0.26 (-0.50,1.03)
>6-12	0.19 (0.05,0.34)	0.44 (0.20,0.73)	0.77 (0.58,0.96)	0.83 (0.00,1.68)	0.16 (-0.41,0.72)	0.05 (-0.07,0.21)	0.04 (-0.06,0.13)	0.50 (-0.73,1.70)
>12	0.38 (0.25,0.51)	0.39 (0.12,0.70)	0.81 (0.63,0.99)	0.83 (-0.02,1.65)	0.14 (-0.31,0.58)	0.02 (-0.59,0.65)	0.06 (-0.04,0.17)	0.53 (-0.85,1.89)
Area of residence								
Rural	0.07 (-0.06,0.20)	0.44 (0.15,0.76)	0.80 (0.64,0.97)	0.68 (-0.09,1.43)	-0.04 (-0.73,0.58)	0.01 (-0.06,0.09)	0.02 (-0.10,0.13)	0.27 (-0.45,0.99)
Urban	0.30 (0.13,0.46)	0.40 (0.14,0.68)	0.79 (0.62,0.96)	0.79 (0.02,1.54)	0.18 (-0.34,0.65)	0.08 (-0.28,0.47)	0.06 (-0.04,0.16)	0.43 (-0.72,1.65)
High vs. Low Educated								
Overall	0.51 (0.22,0.76)	-0.14 (-0.76,0.36)	-0.05 (-0.33,0.23)	1.31 (0.02,2.60)	-1.23 (-2.21,-0.45)	2.00 (1.23,3.41)	0.13 (-0.05,0.26)	4.68 (2.56,7.34)
Sex								
Female	0.37 (0.08,0.62)	-0.14 (-0.70,0.33)	-0.05 (-0.32,0.21)	1.23 (0.00,2.48)	-1.28 (-2.29,-0.48)	2.01 (1.23,3.44)	0.12 (-0.07,0.25)	4.54 (2.44,7.10)
Male	0.65 (0.35,0.92)	-0.14 (-0.84,0.41)	-0.05 (-0.38,0.26)	1.39 (0.02,2.75)	-1.18 (-2.21,-0.40)	1.99 (1.22,3.43)	0.15 (-0.04,0.28)	4.81 (2.59,7.65)
Age (years)								
20-39	1.12 (0.72,1.48)	-0.20 (-1.16,0.56)	-0.25 (-0.67,0.18)	1.67 (0.06,3.26)	-1.63 (-2.92,-0.58)	2.31 (1.40,3.98)	0.23 (-0.04,0.43)	5.08 (2.77,7.90)
39-59	0.48 (0.25,0.69)	-0.14 (-0.64,0.27)	-0.15 (-0.41,0.10)	1.20 (0.06,2.37)	-0.75 (-1.42,-0.18)	1.73 (1.07,2.98)	0.12 (-0.02,0.21)	4.37 (2.37,7.03)
60+	0.19 (0.03,0.33)	-0.12 (-0.46,0.17)	-0.05 (-0.19,0.09)	0.83 (0.02,1.70)	-0.68 (-1.26,-0.23)	1.45 (0.85,2.54)	0.14 (0.04,0.21)	3.23 (1.64,5.69)
Area of residence								
Rural	0.56 (0.30,0.80)	-0.16 (-0.86,0.42)	0.03 (-0.24,0.30)	1.32 (0.03,2.66)	-1.92 (-3.42,-0.85)	1.02 (0.64,1.70)	-0.07 (-0.25,0.07)	3.52 (1.70,5.83)
Urban	0.15 (-0.20,0.46)	-0.13 (-0.71,0.35)	-0.07 (-0.35,0.20)	1.30 (0.01,2.60)	-0.84 (-1.58,-0.18)	4.07 (2.38,7.24)	0.27 (0.10,0.41)	5.77 (3.15,9.04)
Urban vs. Rural Areas								
Overall	1.17 (0.98,1.36)	-0.35 (-0.72,-0.01)	0.05 (-0.17,0.27)	0.11 (-0.69,0.90)	-0.81 (-1.81,-0.03)	1.17 (0.65,2.04)	0.01 (-0.08,0.09)	2.67 (1.26,4.45)
Sex								
Female	1.06 (0.87,1.26)	-0.33 (-0.68,0.00)	0.05 (-0.14,0.25)	0.05 (-0.74,0.82)	-0.93 (-1.94,-0.12)	1.13 (0.62,2.00)	-0.01 (-0.11,0.07)	2.60 (1.17,4.41)
Male	1.28 (1.09,1.49)	-0.37 (-0.80,0.03)	0.04 (-0.21,0.29)	0.18 (-0.69,1.02)	-0.70 (-1.74,0.09)	1.20 (0.66,2.11)	0.03 (-0.07,0.12)	2.75 (1.23,4.73)
Age (years)								
20-39	1.75 (1.48,2.03)	-0.48 (-1.06,0.06)	0.01 (-0.33,0.36)	0.27 (-0.74,1.26)	-1.12 (-2.42,-0.08)	1.42 (0.79,2.45)	-0.01 (-0.15,0.12)	2.93 (1.31,4.97)
39-59	1.01 (0.86,1.17)	-0.24 (-0.52,0.04)	0.04 (-0.16,0.24)	0.12 (-0.61,0.82)	-0.40 (-1.08,0.15)	0.95 (0.50,1.74)	0.05 (-0.02,0.10)	2.49 (1.13,4.29)
60+	0.60 (0.50,0.70)	-0.16 (-0.35,0.02)	0.03 (-0.09,0.14)	0.07 (-0.46,0.56)	-0.43 (-1.01,0.02)	0.71 (0.37,1.34)	0.09 (0.04,0.13)	1.77 (0.78,3.17)
Education (years)								
0-6	1.20 (0.95,1.47)	-0.35 (-0.73,0.01)	0.07 (-0.15,0.30)	0.15 (-0.62,0.90)	-1.18 (-2.52,-0.18)	0.94 (0.49,1.76)	-0.14 (-0.26,-0.06)	1.90 (0.65,3.64)
>6-12	1.27 (1.07,1.48)	-0.39 (-0.79,-0.02)	0.15 (-0.07,0.38)	0.00 (-0.89,0.88)	-0.35 (-1.05,0.29)	1.01 (0.54,1.80)	0.04 (-0.04,0.12)	3.70 (1.69,6.09)
>12	0.78 (0.62,0.95)	-0.31 (-0.72,0.04)	-0.03 (-0.25,0.19)	0.14 (-0.81,1.01)	-0.08 (-0.61,0.42)	3.99 (2.19,7.21)	0.20 (0.10,0.29)	4.17 (2.04,6.73)

*Data are mean absolute difference in intakes (95% uncertainty interval) in 8 oz servings per week. All intakes are reported adjusted to 2,000 kcal/d for ages 11 to 74 years, and 1,700 kcal/d for ages 75+ years. Standardized serving size used for this analysis: 8 oz serving = 248 grams. †Referred to as Former Soviet Union in previous Global Dietary Database reports. §Referred to as Asia in previous Global Dietary Database reports. Source data are provided as Source Data files 3-5.

Supplementary Table 8. Absolute difference in sugar-sweetened beverage intakes (8 oz serving/week) in males vs. females in the 25 most populous countries in adults (20+years) in 2018.

		Age (years)			Education (years)			Area of residence	
	Overall	20-39	40-59	60+	≤6 years	>6-12 years	>12	Rural	Urban
Country	*mean (95% UI)*	*mean (95% UI)*	*mean (95% UI)*	*mean (95% UI)*	*mean (95% UI)*	*mean (95% UI)*	*mean (95% UI)*	*mean (95% UI)*	*mean (95% UI)*
China	0.01 (-0.05,0.07)	0.01 (-0.08,0.10)	0.00 (-0.04,0.05)	0.00 (-0.02,0.03)	0.01 (-0.04,0.06)	0.01 (-0.05,0.07)	0.01 (-0.05,0.07)	0.01 (-0.05,0.07)	0.01 (-0.05,0.07)
India	0.06 (0.01,0.14)	0.07 (0.01,0.16)	0.05 (0.01,0.12)	0.04 (0.01,0.09)	0.04 (0.01,0.11)	0.08 (0.01,0.19)	0.11 (0.02,0.26)	0.03 (0.00,0.06)	0.13 (0.02,0.32)
United States	1.05 (0.76,1.35)	1.33 (0.90,1.78)	0.79 (0.53,1.05)	0.51 (0.36,0.67)	1.35 (0.97,1.74)	1.33 (0.95,1.71)	0.98 (0.70,1.26)	1.09 (0.79,1.41)	1.04 (0.75,1.34)
Indonesia	0.10 (-0.14,0.36)	0.13 (-0.22,0.50)	0.06 (-0.11,0.23)	0.04 (-0.06,0.15)	0.10 (-0.13,0.34)	0.12 (-0.16,0.40)	0.12 (-0.16,0.40)	0.10 (-0.14,0.36)	0.11 (-0.15,0.37)
Brazil	0.49 (-0.28,1.30)	0.50 (-0.47,1.50)	0.35 (-0.32,1.04)	0.28 (-0.20,0.79)	0.47 (-0.26,1.24)	0.51 (-0.28,1.34)	0.51 (-0.29,1.35)	0.44 (-0.24,1.16)	0.50 (-0.28,1.31)
Pakistan	0.01 (-1.41,1.37)	0.01 (-1.60,1.52)	0.01 (-1.22,1.20)	0.00 (-0.94,0.92)	0.01 (-1.03,1.04)	0.02 (-1.91,1.86)	0.02 (-2.67,2.50)	0.01 (-0.60,0.59)	0.02 (-2.84,2.80)
Russia	0.48 (-0.03,1.05)	0.43 (-0.34,1.26)	0.23 (-0.18,0.67)	0.19 (-0.10,0.51)	0.47 (-0.03,1.05)	0.51 (-0.04,1.14)	0.47 (-0.03,1.03)	0.51 (-0.03,1.12)	0.47 (-0.03,1.02)
Japan	0.39 (0.03,0.77)	0.53 (-0.16,1.23)	0.25 (-0.07,0.59)	0.17 (-0.01,0.37)	0.33 (0.02,0.65)	0.40 (0.03,0.78)	0.39 (0.03,0.78)	0.38 (0.03,0.74)	0.39 (0.03,0.77)
Bangladesh	0.02 (-0.04,0.08)	0.02 (-0.04,0.09)	0.01 (-0.03,0.07)	0.01 (-0.02,0.05)	0.01 (-0.03,0.06)	0.02 (-0.05,0.10)	0.03 (-0.07,0.14)	0.01 (-0.02,0.03)	0.03 (-0.08,0.16)
Nigeria	0.25 (-1.53,2.11)	0.24 (-2.44,2.92)	0.22 (-2.14,2.63)	0.15 (-1.52,2.03)	0.20 (-1.93,2.41)	0.39 (-3.09,3.86)	0.41 (-3.56,4.59)	0.20 (-1.65,2.19)	0.32 (-2.68,3.40)
Mexico	1.26 (0.04,2.46)	1.43 (-0.08,2.90)	0.96 (-0.09,1.99)	0.72 (-0.03,1.48)	1.20 (0.03,2.38)	1.30 (0.04,2.52)	1.31 (0.04,2.59)	1.15 (0.03,2.22)	1.30 (0.04,2.53)
Germany	0.88 (0.46,1.34)	1.20 (0.52,1.93)	0.71 (0.30,1.14)	0.45 (0.22,0.70)	0.99 (0.51,1.51)	0.97 (0.50,1.49)	0.71 (0.37,1.08)	0.91 (0.47,1.39)	0.87 (0.45,1.33)
Vietnam	0.09 (-0.34,0.50)	0.03 (-0.60,0.62)	0.01 (-0.28,0.29)	0.03 (-0.15,0.19)	0.08 (-0.31,0.46)	0.10 (-0.37,0.54)	0.10 (-0.37,0.54)	0.09 (-0.34,0.49)	0.09 (-0.35,0.52)
Philippines	-0.39 (-1.12,0.37)	-0.67 (-1.68,0.38)	-0.30 (-0.76,0.17)	-0.16 (-0.45,0.13)	-0.34 (-0.99,0.32)	-0.41 (-1.17,0.38)	-0.41 (-1.18,0.38)	-0.38 (-1.10,0.36)	-0.40 (-1.13,0.38)
Egypt	-0.48 (-0.95,-0.03)	-0.63 (-1.19,-0.08)	-0.38 (-0.73,-0.05)	-0.35 (-0.68,-0.04)	-0.48 (-0.95,-0.03)	-0.52 (-1.02,-0.03)	-0.43 (-0.86,-0.03)	-0.51 (-1.00,-0.03)	-0.44 (-0.87,-0.03)
Iran	0.12 (-0.34,0.56)	0.15 (-0.40,0.69)	0.10 (-0.25,0.44)	0.09 (-0.23,0.40)	0.12 (-0.34,0.57)	0.13 (-0.36,0.62)	0.10 (-0.30,0.50)	0.13 (-0.37,0.63)	0.11 (-0.32,0.54)
Turkey	0.12 (-0.61,0.87)	0.09 (-0.85,1.03)	0.05 (-0.53,0.63)	0.05 (-0.49,0.58)	0.12 (-0.61,0.87)	0.13 (-0.65,0.91)	0.11 (-0.54,0.75)	0.14 (-0.68,0.97)	0.12 (-0.59,0.83)
Thailand	0.49 (-1.00,2.19)	0.55 (-1.97,3.21)	0.24 (-0.92,1.44)	0.17 (-0.53,0.92)	0.46 (-0.93,2.03)	0.55 (-1.11,2.47)	0.55 (-1.11,2.46)	0.48 (-0.97,2.15)	0.50 (-1.02,2.24)
Ethiopia	0.43 (-1.34,2.21)	0.41 (-1.53,2.38)	0.37 (-1.25,1.96)	0.24 (-0.93,1.40)	0.35 (-1.11,1.78)	0.60 (-1.85,3.16)	0.67 (-2.08,3.57)	0.39 (-1.21,1.97)	0.61 (-1.91,3.19)
United Kingdom	0.23 (-0.42,0.85)	0.18 (-0.82,1.12)	0.11 (-0.50,0.66)	0.11 (-0.23,0.43)	0.25 (-0.46,0.93)	0.25 (-0.45,0.90)	0.18 (-0.33,0.66)	0.24 (-0.44,0.89)	0.23 (-0.42,0.84)
Italy	0.35 (0.11,0.61)	0.45 (0.06,0.86)	0.27 (0.04,0.52)	0.18 (0.05,0.32)	0.38 (0.12,0.66)	0.38 (0.12,0.65)	0.28 (0.09,0.48)	0.36 (0.12,0.63)	0.35 (0.11,0.60)
France	1.01 (0.56,1.52)	1.41 (0.71,2.19)	0.85 (0.42,1.32)	0.53 (0.29,0.79)	1.13 (0.62,1.68)	1.11 (0.62,1.66)	0.81 (0.45,1.22)	1.05 (0.59,1.57)	1.00 (0.56,1.50)
Korea	0.37 (0.30,0.45)	0.54 (0.42,0.67)	0.25 (0.19,0.31)	0.16 (0.13,0.20)	0.31 (0.25,0.39)	0.38 (0.30,0.46)	0.38 (0.30,0.46)	0.36 (0.29,0.44)	0.37 (0.30,0.46)
Spain	0.39 (-0.32,1.08)	0.41 (-0.69,1.47)	0.26 (-0.42,0.92)	0.19 (-0.19,0.56)	0.41 (-0.34,1.15)	0.41 (-0.33,1.14)	0.30 (-0.24,0.84)	0.40 (-0.33,1.12)	0.39 (-0.32,1.07)
DR Congo	0.21 (-0.46,1.32)	0.21 (-0.54,1.41)	0.18 (-0.43,1.17)	0.13 (-0.32,0.82)	0.17 (-0.38,1.11)	0.29 (-0.69,1.85)	0.32 (-0.73,2.06)	0.17 (-0.38,1.04)	0.27 (-0.59,1.68)

*Data are mean absolute difference in intakes (95% uncertainty interval) in 8 oz servings per week. Standardized serving size used for this analysis: 8 oz serving = 248 grams. Countries are ordered top to bottom from most to least populous based on 2018 adult (20+ years) population data. Source data are provided as Source Data file 9.

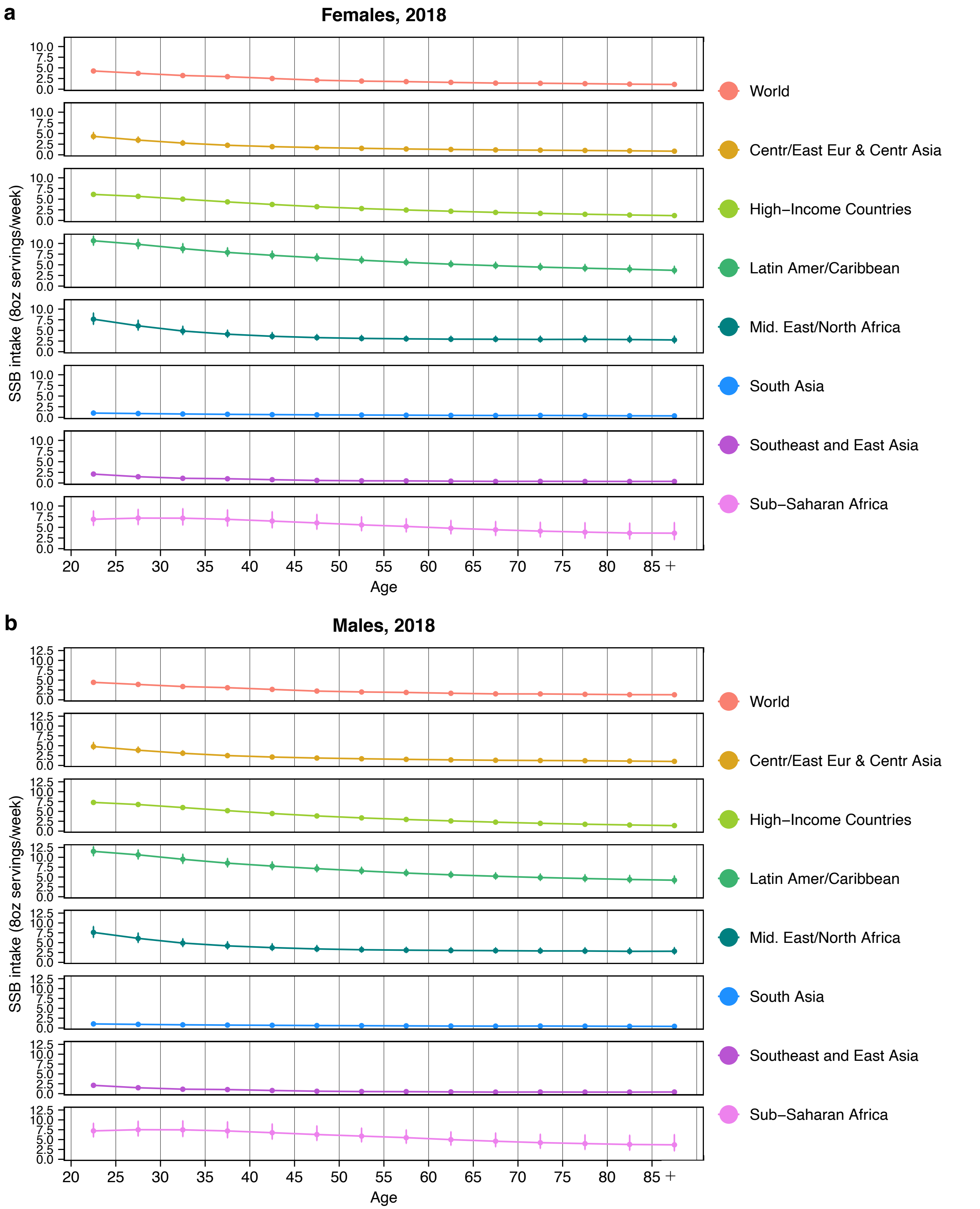

Supplementary Figure 2. **Global and regional sugar-sweetened beverage intakes (8 oz servings/week) by age among females (a) and males (b) adults (+20 years) in 2018**. SSBs were defined as any beverage with added sugars and ≥50 kcal per 8 oz serving, including commercial or homemade beverages, soft drinks, energy drinks, fruit drinks, punch, lemonade, and aguas frescas. This definition excludes 100% fruit and vegetable juices, non-caloric artificially sweetened drinks, and sweetened milks. The standardized serving size used for this analysis is 8 oz serving (248 grams). The filled circles represent the mean SSBs intake (8 oz serving/week) and the error bars the 95% UIs. Age groups are 20-24, 25-29, 30-34, 35-39, 40-44, 45-49, 50-54, 55-59, 60-64, 65-69, 70-74, 75-79, 80-84, 85+ years. In prior GDD reports, the region Central/ Eastern Europe and Central Asia was referred as Former Soviet Union, and Southeast and East Asia was referred as Asia. Source data are provided as Source Data file 2.

GDD, Global Dietary Database; oz, ounces; SSBs, sugar-sweetened beverages; UIs, uncertainty intervals.

Supplementary Table 9. Absolute difference in sugar-sweetened beverage intakes (8 oz serving/week) in high vs. low educated adults in the 25 most populous countries in adults (20+years) in 2018.

		Sex		Age (years)			Area of residence	
	Overall	Female	Male	20-39	40-59	60+	Rural	Urban
Country	*mean (95% UI)*	*mean (95% UI)*	*mean (95% UI)*	*mean (95% UI)*	*mean (95% UI)*	*mean (95% UI)*	*mean (95% UI)*	*mean (95% UI)*
China	0.04 (0.02,0.06)	0.04 (0.01,0.06)	0.04 (0.02,0.07)	0.06 (0.02,0.10)	0.03 (0.01,0.05)	0.02 (0.01,0.03)	0.04 (0.01,0.06)	0.04 (0.02,0.07)
India	0.19 (0.09,0.37)	0.16 (0.07,0.32)	0.22 (0.10,0.44)	0.22 (0.10,0.44)	0.17 (0.08,0.33)	0.13 (0.06,0.25)	0.09 (0.04,0.17)	0.40 (0.18,0.81)
United States	-1.73 (-2.16,-1.34)	-1.55 (-1.92,-1.20)	-1.92 (-2.40,-1.48)	-2.59 (-3.24,-2.00)	-1.53 (-1.91,-1.19)	-0.88 (-1.10,-0.68)	-1.80 (-2.21,-1.40)	-1.71 (-2.14,-1.33)
Indonesia	0.16 (0.06,0.28)	0.15 (0.06,0.27)	0.17 (0.07,0.30)	0.23 (0.09,0.40)	0.11 (0.04,0.19)	0.07 (0.02,0.12)	0.16 (0.06,0.28)	0.16 (0.06,0.29)
Brazil	0.36 (-0.37,1.09)	0.34 (-0.35,1.04)	0.38 (-0.39,1.15)	0.44 (-0.46,1.36)	0.31 (-0.32,0.95)	0.22 (-0.23,0.69)	0.32 (-0.33,0.99)	0.36 (-0.38,1.11)
Pakistan	4.42 (1.89,9.46)	4.42 (1.86,9.55)	4.43 (1.91,9.48)	5.05 (2.14,10.54)	3.77 (1.60,8.40)	2.93 (1.22,6.48)	1.92 (0.79,4.21)	8.82 (3.77,19.11)
Russia	0.01 (-0.64,0.51)	0.01 (-0.58,0.47)	0.01 (-0.72,0.57)	0.01 (-0.98,0.78)	0.01 (-0.51,0.41)	0.00 (-0.35,0.29)	0.01 (-0.68,0.55)	0.01 (-0.63,0.50)
Japan	0.31 (0.13,0.49)	0.28 (0.12,0.44)	0.34 (0.14,0.54)	0.58 (0.24,0.92)	0.28 (0.12,0.44)	0.16 (0.06,0.25)	0.30 (0.12,0.47)	0.31 (0.13,0.49)
Bangladesh	0.23 (0.10,0.48)	0.22 (0.09,0.48)	0.23 (0.10,0.50)	0.26 (0.11,0.55)	0.20 (0.08,0.43)	0.15 (0.06,0.33)	0.10 (0.04,0.22)	0.46 (0.20,1.00)
Nigeria	3.47 (0.97,7.11)	3.31 (0.35,7.64)	3.56 (0.34,8.19)	3.85 (0.56,8.66)	3.07 (0.22,7.37)	2.16 (0.12,5.70)	2.69 (0.36,6.50)	4.25 (0.43,9.47)
Mexico	0.72 (-0.75,2.21)	0.67 (-0.69,2.07)	0.77 (-0.79,2.38)	0.89 (-0.93,2.72)	0.61 (-0.64,1.90)	0.44 (-0.45,1.39)	0.65 (-0.66,2.02)	0.74 (-0.77,2.26)
Germany	-0.85 (-1.07,-0.65)	-0.71 (-0.92,-0.54)	-0.99 (-1.26,-0.75)	-1.36 (-1.73,-1.05)	-0.81 (-1.02,-0.62)	-0.45 (-0.57,-0.35)	-0.88 (-1.10,-0.69)	-0.84 (-1.07,-0.65)
Vietnam	0.27 (0.11,0.46)	0.27 (0.10,0.46)	0.28 (0.11,0.49)	0.40 (0.15,0.67)	0.18 (0.07,0.31)	0.11 (0.04,0.19)	0.27 (0.11,0.46)	0.28 (0.11,0.48)
Philippines	0.55 (0.22,0.87)	0.58 (0.23,0.93)	0.51 (0.21,0.84)	0.75 (0.31,1.20)	0.34 (0.14,0.55)	0.21 (0.08,0.34)	0.53 (0.22,0.86)	0.56 (0.23,0.89)
Egypt	-0.32 (-0.71,0.03)	-0.35 (-0.78,0.03)	-0.30 (-0.66,0.03)	-0.40 (-0.87,0.04)	-0.24 (-0.53,0.02)	-0.22 (-0.49,0.02)	-0.34 (-0.75,0.03)	-0.30 (-0.66,0.03)
Iran	-0.32 (-0.69,0.03)	-0.31 (-0.68,0.03)	-0.32 (-0.70,0.03)	-0.39 (-0.84,0.04)	-0.25 (-0.53,0.02)	-0.23 (-0.48,0.02)	-0.35 (-0.76,0.03)	-0.31 (-0.66,0.03)
Turkey	-0.31 (-0.73,0.03)	-0.30 (-0.72,0.03)	-0.32 (-0.75,0.03)	-0.40 (-0.93,0.04)	-0.24 (-0.57,0.02)	-0.22 (-0.52,0.02)	-0.35 (-0.81,0.03)	-0.30 (-0.70,0.03)
Thailand	0.80 (0.27,1.91)	0.76 (0.25,1.87)	0.85 (0.29,2.03)	1.31 (0.45,3.11)	0.60 (0.20,1.44)	0.37 (0.12,0.88)	0.79 (0.27,1.88)	0.82 (0.28,1.94)
Ethiopia	5.05 (2.44,8.71)	4.90 (2.34,8.58)	5.21 (2.48,9.16)	5.57 (2.69,9.67)	4.59 (2.21,8.02)	3.20 (1.42,6.20)	4.55 (2.14,7.97)	7.20 (3.58,12.51)
United Kingdom	-1.31 (-1.64,-1.01)	-1.28 (-1.61,-0.97)	-1.35 (-1.71,-1.02)	-2.00 (-2.51,-1.53)	-1.20 (-1.50,-0.92)	-0.67 (-0.84,-0.52)	-1.36 (-1.70,-1.05)	-1.30 (-1.63,-1.00)
Italy	-0.46 (-0.58,-0.36)	-0.41 (-0.52,-0.31)	-0.51 (-0.66,-0.39)	-0.75 (-0.95,-0.58)	-0.45 (-0.57,-0.35)	-0.25 (-0.31,-0.19)	-0.48 (-0.60,-0.37)	-0.45 (-0.57,-0.35)
France	-0.87 (-1.08,-0.66)	-0.71 (-0.91,-0.54)	-1.03 (-1.30,-0.78)	-1.37 (-1.71,-1.04)	-0.82 (-1.03,-0.63)	-0.45 (-0.57,-0.35)	-0.90 (-1.12,-0.69)	-0.86 (-1.08,-0.65)
Korea	0.15 (0.06,0.23)	0.12 (0.05,0.19)	0.18 (0.07,0.28)	0.25 (0.10,0.39)	0.11 (0.05,0.18)	0.07 (0.03,0.11)	0.14 (0.06,0.23)	0.15 (0.06,0.24)
Spain	-0.92 (-1.20,-0.69)	-0.86 (-1.15,-0.63)	-0.97 (-1.29,-0.72)	-1.42 (-1.86,-1.06)	-0.88 (-1.15,-0.66)	-0.48 (-0.63,-0.36)	-0.95 (-1.24,-0.71)	-0.91 (-1.19,-0.68)
DR Congo	1.40 (0.42,4.20)	1.32 (0.40,3.94)	1.48 (0.43,4.40)	1.55 (0.46,4.62)	1.26 (0.38,3.77)	0.89 (0.25,2.83)	1.12 (0.33,3.35)	1.78 (0.53,5.24)

*Data are mean absolute difference in intakes (95% uncertainty interval) in 8 oz servings per week. Standardized serving size used for this analysis: 8 oz serving = 248 grams. Countries are ordered top to bottom from most to least populous based on 2018 adult (20+ years) population data. Source data are provided as Source Data file 10.

Supplementary Table 10. Absolute difference in sugar-sweetened beverage intakes (8 oz serving/week) in urban vs. rural areas in the 25 most populous countries in adults (20+years) in 2018.

		Sex		Age (years)			Education (years)		
	Overall	Female	Male	20-39	40-59	60+	≤6 years	>6-12 years	>12
Country	*mean (95% UI)*	*mean (95% UI)*	*mean (95% UI)*	*mean (95% UI)*	*mean (95% UI)*	*mean (95% UI)*	*mean (95% UI)*	*mean (95% UI)*	*mean (95% UI)*
China	0.01 (-0.01,0.03)	0.01 (-0.01,0.03)	0.01 (-0.01,0.03)	0.01 (-0.01,0.04)	0.01 (-0.01,0.02)	0.00 (0.00,0.01)	0.01 (-0.01,0.02)	0.01 (-0.01,0.03)	0.01 (-0.01,0.03)
India	0.31 (0.16,0.57)	0.25 (0.12,0.48)	0.36 (0.18,0.68)	0.36 (0.18,0.68)	0.27 (0.13,0.52)	0.21 (0.10,0.40)	0.22 (0.11,0.43)	0.40 (0.20,0.77)	0.54 (0.28,1.03)
United States	-0.23 (-0.52,0.07)	-0.20 (-0.46,0.06)	-0.25 (-0.58,0.08)	-0.34 (-0.78,0.11)	-0.20 (-0.46,0.06)	-0.12 (-0.26,0.04)	-0.29 (-0.66,0.09)	-0.29 (-0.65,0.09)	-0.21 (-0.48,0.07)
Indonesia	0.04 (-0.03,0.11)	0.03 (-0.03,0.11)	0.04 (-0.03,0.12)	0.05 (-0.05,0.16)	0.02 (-0.02,0.08)	0.01 (-0.01,0.05)	0.03 (-0.03,0.10)	0.04 (-0.04,0.13)	0.04 (-0.04,0.12)
Brazil	0.53 (0.12,0.97)	0.51 (0.11,0.92)	0.57 (0.13,1.03)	0.67 (0.15,1.21)	0.47 (0.11,0.84)	0.34 (0.08,0.61)	0.51 (0.11,0.94)	0.55 (0.13,1.00)	0.56 (0.12,1.01)
Pakistan	6.54 (3.53,12.21)	6.50 (3.43,12.52)	6.54 (3.51,12.33)	7.47 (4.10,13.65)	5.60 (2.88,11.02)	4.33 (2.09,8.63)	4.81 (2.44,9.50)	8.84 (4.60,16.97)	11.80 (6.26,22.74)
Russia	-0.21 (-0.57,0.14)	-0.19 (-0.52,0.12)	-0.23 (-0.64,0.15)	-0.31 (-0.87,0.21)	-0.16 (-0.46,0.11)	-0.11 (-0.32,0.07)	-0.20 (-0.56,0.14)	-0.22 (-0.61,0.14)	-0.20 (-0.56,0.13)
Japan	0.07 (-0.07,0.21)	0.07 (-0.06,0.19)	0.08 (-0.08,0.24)	0.14 (-0.13,0.40)	0.07 (-0.06,0.19)	0.04 (-0.04,0.11)	0.06 (-0.06,0.18)	0.08 (-0.07,0.22)	0.08 (-0.07,0.22)
Bangladesh	0.36 (0.20,0.65)	0.35 (0.19,0.63)	0.38 (0.20,0.67)	0.42 (0.23,0.73)	0.32 (0.17,0.58)	0.24 (0.12,0.46)	0.25 (0.13,0.47)	0.47 (0.25,0.85)	0.63 (0.33,1.19)
Nigeria	2.14 (0.30,4.81)	2.06 (-0.25,5.34)	2.14 (-0.17,5.62)	2.34 (-0.21,5.84)	1.93 (-0.17,5.11)	1.34 (-0.22,3.75)	1.78 (-0.24,4.64)	2.99 (-0.48,7.41)	3.29 (-0.55,8.26)
Mexico	1.11 (0.24,2.01)	1.03 (0.22,1.87)	1.19 (0.25,2.16)	1.36 (0.29,2.49)	0.94 (0.20,1.71)	0.68 (0.15,1.25)	1.05 (0.23,1.92)	1.13 (0.24,2.08)	1.15 (0.24,2.08)
Germany	-0.13 (-0.30,0.04)	-0.11 (-0.26,0.03)	-0.15 (-0.35,0.05)	-0.21 (-0.48,0.06)	-0.12 (-0.29,0.04)	-0.07 (-0.16,0.02)	-0.14 (-0.33,0.04)	-0.14 (-0.33,0.04)	-0.10 (-0.24,0.03)
Vietnam	0.06 (-0.06,0.20)	0.06 (-0.06,0.19)	0.07 (-0.06,0.20)	0.09 (-0.09,0.28)	0.04 (-0.04,0.13)	0.03 (-0.02,0.08)	0.06 (-0.05,0.18)	0.07 (-0.06,0.21)	0.07 (-0.06,0.21)
Philippines	0.13 (-0.12,0.38)	0.14 (-0.13,0.40)	0.12 (-0.11,0.36)	0.18 (-0.17,0.53)	0.08 (-0.08,0.24)	0.05 (-0.05,0.15)	0.12 (-0.11,0.33)	0.14 (-0.13,0.40)	0.14 (-0.13,0.40)
Egypt	-0.39 (-0.77,-0.05)	-0.42 (-0.84,-0.05)	-0.35 (-0.71,-0.04)	-0.48 (-0.94,-0.06)	-0.29 (-0.58,-0.04)	-0.27 (-0.55,-0.03)	-0.39 (-0.77,-0.05)	-0.42 (-0.84,-0.05)	-0.34 (-0.69,-0.04)
Iran	-0.39 (-0.78,-0.05)	-0.38 (-0.77,-0.05)	-0.40 (-0.81,-0.05)	-0.48 (-0.94,-0.06)	-0.30 (-0.61,-0.04)	-0.28 (-0.57,-0.03)	-0.40 (-0.79,-0.05)	-0.43 (-0.85,-0.05)	-0.35 (-0.70,-0.04)
Turkey	-0.40 (-0.84,-0.05)	-0.39 (-0.82,-0.05)	-0.40 (-0.86,-0.05)	-0.51 (-1.07,-0.06)	-0.31 (-0.66,-0.04)	-0.28 (-0.61,-0.04)	-0.39 (-0.83,-0.05)	-0.42 (-0.89,-0.05)	-0.34 (-0.74,-0.04)
Thailand	0.18 (-0.18,0.71)	0.17 (-0.17,0.68)	0.19 (-0.18,0.73)	0.29 (-0.29,1.15)	0.13 (-0.13,0.54)	0.08 (-0.08,0.32)	0.17 (-0.16,0.66)	0.20 (-0.20,0.80)	0.20 (-0.20,0.79)
Ethiopia	3.70 (1.86,5.96)	3.58 (1.77,5.86)	3.80 (1.92,6.25)	4.09 (2.03,6.62)	3.34 (1.69,5.44)	2.32 (1.13,4.17)	3.03 (1.48,5.04)	5.09 (2.65,8.29)	5.67 (2.90,9.45)
United Kingdom	-0.20 (-0.48,0.06)	-0.20 (-0.47,0.06)	-0.21 (-0.49,0.06)	-0.31 (-0.74,0.09)	-0.19 (-0.44,0.06)	-0.11 (-0.25,0.03)	-0.22 (-0.52,0.07)	-0.22 (-0.51,0.07)	-0.16 (-0.38,0.05)
Italy	-0.07 (-0.16,0.02)	-0.06 (-0.15,0.02)	-0.08 (-0.19,0.02)	-0.12 (-0.27,0.03)	-0.07 (-0.16,0.02)	-0.04 (-0.09,0.01)	-0.08 (-0.18,0.02)	-0.08 (-0.18,0.02)	-0.06 (-0.13,0.02)
France	-0.13 (-0.31,0.04)	-0.11 (-0.26,0.03)	-0.16 (-0.37,0.05)	-0.21 (-0.49,0.06)	-0.12 (-0.29,0.04)	-0.07 (-0.16,0.02)	-0.15 (-0.34,0.05)	-0.14 (-0.34,0.04)	-0.11 (-0.25,0.03)
Korea	0.04 (-0.03,0.11)	0.03 (-0.03,0.08)	0.04 (-0.04,0.13)	0.06 (-0.06,0.18)	0.03 (-0.03,0.08)	0.02 (-0.02,0.05)	0.03 (-0.03,0.09)	0.04 (-0.03,0.11)	0.04 (-0.03,0.11)
Spain	-0.15 (-0.35,0.04)	-0.14 (-0.33,0.04)	-0.15 (-0.37,0.05)	-0.23 (-0.54,0.07)	-0.14 (-0.34,0.04)	-0.08 (-0.18,0.02)	-0.16 (-0.37,0.05)	-0.15 (-0.36,0.05)	-0.11 (-0.27,0.03)
DR Congo	0.90 (0.28,2.77)	0.84 (0.26,2.63)	0.94 (0.29,2.99)	0.99 (0.31,3.08)	0.81 (0.25,2.52)	0.57 (0.17,1.79)	0.75 (0.23,2.41)	1.25 (0.39,3.86)	1.40 (0.44,4.31)

*Data are mean absolute change in intakes (95% uncertainty interval) in 8 oz servings per week. Standardized serving size used for this analysis: 8 oz serving = 248 grams. Countries are ordered top to bottom from most to least populous based on 2018 adult (20+ years) population data. Source data are provided as Source Data file 11.

Supplementary Table 11. Global and regional mean (95% UI) sugar-sweetened beverage intakes (8 oz serving/week) in adults (20+ years) by age, sex, education, and area of residence across 185 countries in 1990.

	World	Central or Eastern Europe and Central Asia†	High-Income Countries	Latin America and Caribbean	Middle East and North Africa	South Asia†	Southeast and East Asia	Sub-Saharan Africa
	mean (95% UI)	*mean (95% UI)*	*mean (95% UI)*	*mean (95% UI)*	*mean (95% UI)*	*mean (95% UI)*	*mean (95% UI)*	*mean (95% UI)*
Overall	2.3 (2.2-2.5)	1.8 (1.6-2.1)	4.0 (3.8-4.2)	8.7 (8.0-9.5)	4.1 (3.6-4.8)	0.6 (0.4-0.8)	0.8 (0.7-0.9)	3.6 (2.9-4.3)
Sex								
Female	2.2 (2.1-2.4)	1.6 (1.4-1.9)	3.6 (3.4-3.7)	8.3 (7.5-9.1)	4.2 (3.6-4.9)	0.5 (0.4-0.8)	0.8 (0.7-0.9)	3.4 (2.8-4.2)
Male	2.4 (2.3-2.6)	2.0 (1.7-2.3)	4.5 (4.3-4.7)	9.1 (8.2-10.0)	4.1 (3.5-4.9)	0.6 (0.4-0.9)	0.8 (0.7-0.9)	3.7 (3.0-4.5)
Age								
20-24	3.3 (3.1-3.4)	3.7 (3.2-4.3)	6.4 (6.2-6.7)	11.5 (10.6-12.6)	6.4 (5.6-7.4)	0.7 (0.5-1.0)	1.3 (1.2-1.4)	3.9 (3.2-4.7)
25-29	3.0 (2.9-3.2)	2.8 (2.4-3.3)	6.0 (5.8-6.3)	10.7 (9.7-11.7)	5.1 (4.4-6.0)	0.7 (0.5-1.0)	1.1 (1.0-1.2)	4.0 (3.3-4.9)
30-34	2.7 (2.6-2.9)	2.2 (1.9-2.6)	5.5 (5.2-5.8)	9.6 (8.7-10.7)	4.2 (3.5-5.0)	0.6 (0.4-0.9)	0.9 (0.8-1.0)	4.0 (3.2-5.0)
35-39	2.3 (2.2-2.5)	1.8 (1.5-2.1)	4.8 (4.6-5.1)	8.7 (7.8-9.8)	3.6 (3.0-4.3)	0.5 (0.3-0.8)	0.7 (0.7-0.8)	3.8 (3.0-4.8)
40-44	2.2 (2.1-2.3)	1.5 (1.3-1.8)	4.1 (3.9-4.3)	7.9 (7.1-8.9)	3.1 (2.6-3.8)	0.5 (0.3-0.8)	0.7 (0.6-0.7)	3.5 (2.8-4.5)
45-49	2.0 (1.8-2.1)	1.3 (1.1-1.6)	3.5 (3.3-3.7)	7.2 (6.4-8.0)	2.9 (2.5-3.5)	0.5 (0.3-0.8)	0.6 (0.5-0.6)	3.3 (2.6-4.2)
50-54	1.7 (1.6-1.8)	1.1 (1.0-1.3)	3.0 (2.8-3.1)	6.5 (5.8-7.3)	2.8 (2.3-3.3)	0.5 (0.3-0.7)	0.5 (0.4-0.5)	3.0 (2.4-3.9)
55-59	1.5 (1.4-1.6)	1.1 (0.9-1.2)	2.6 (2.4-2.7)	6.0 (5.3-6.7)	2.7 (2.3-3.2)	0.4 (0.3-0.7)	0.4 (0.4-0.5)	2.8 (2.1-3.6)
60-64	1.4 (1.3-1.5)	1.0 (0.8-1.1)	2.3 (2.2-2.4)	5.5 (4.9-6.2)	2.7 (2.2-3.2)	0.4 (0.3-0.7)	0.4 (0.4-0.4)	2.5 (1.9-3.4)
65-69	1.3 (1.3-1.4)	0.9 (0.8-1.1)	2.0 (1.9-2.1)	5.1 (4.5-5.9)	2.7 (2.2-3.2)	0.4 (0.2-0.7)	0.4 (0.3-0.4)	2.4 (1.7-3.3)
70-74	1.3 (1.2-1.3)	0.8 (0.7-1.0)	1.9 (1.7-2.0)	4.8 (4.1-5.6)	2.7 (2.2-3.4)	0.4 (0.2-0.7)	0.3 (0.3-0.3)	2.2 (1.5-3.3)
75-79	1.2 (1.1-1.3)	0.8 (0.6-1.0)	1.6 (1.5-1.7)	4.6 (3.8-5.5)	2.7 (2.2-3.4)	0.4 (0.2-0.7)	0.3 (0.3-0.4)	2.1 (1.4-3.3)
80-84	1.2 (1.1-1.3)	0.7 (0.6-0.9)	1.4 (1.3-1.5)	4.4 (3.6-5.3)	2.7 (2.1-3.5)	0.4 (0.2-0.7)	0.3 (0.3-0.4)	2.1 (1.3-3.4)
85+	1.2 (1.1-1.3)	0.7 (0.5-0.9)	1.3 (1.2-1.4)	4.2 (3.3-5.2)	2.7 (2.0-3.5)	0.4 (0.2-0.8)	0.3 (0.3-0.4)	1.9 (1.2-3.4)
Education years								
0-6	1.9 (1.7-2.0)	1.8 (1.4-2.3)	3.7 (3.4-3.9)	8.1 (7.2-9.1)	4.3 (3.6-5.2)	0.4 (0.3-0.7)	0.5 (0.5-0.6)	2.5 (2.0-3.3)
>6-12	2.4 (2.3-2.6)	1.9 (1.6-2.3)	3.9 (3.7-4.1)	9.1 (8.2-10.1)	4.2 (3.6-5.0)	0.5 (0.3-0.8)	0.8 (0.8-0.9)	5.3 (4.3-6.5)
>12	2.9 (2.8-3.1)	1.7 (1.4-2.0)	4.1 (3.9-4.4)	9.0 (7.8-10.3)	3.6 (3.1-4.1)	2.0 (1.3-3.2)	1.1 (1.0-1.2)	7.1 (5.5-9.1)
Area of residence								
Rural	1.5 (1.4-1.6)	2.1 (1.8-2.5)	4.0 (3.8-4.2)	8.1 (7.2-9.1)	4.5 (3.7-5.4)	0.3 (0.2-0.4)	0.6 (0.5-0.7)	2.7 (2.2-3.4)
Urban	3.3 (3.2-3.5)	1.6 (1.4-1.9)	4.0 (3.8-4.2)	8.9 (8.1-9.8)	3.9 (3.4-4.5)	1.4 (0.9-2.3)	1.2 (1.1-1.3)	5.7 (4.6-7.2)

*Data are the mean intakes (95% uncertainty interval) in 8 oz servings per week. All intakes are reported adjusted to 2,000 kcal/d for ages 20 to 74 years, and 1,700 kcal/d for ages 75+ years. Data are based on a Bayesian model that incorporated up to 451 individual-level dietary surveys, and additional survey-level and country-level covariates, to estimate dietary consumption levels. Total sugar-sweetened beverages (SSBs) intake was defined as any beverage with added sugars having ≥50 kcal per 8 oz serving, including commercial or homemade beverages, soft drinks, energy drinks, fruit drinks, punch, lemonade, and aguas frescas. This definition excludes 100% fruit and vegetable juices and non-caloric artificially sweetened drinks. Standardized serving size used for this analysis: 8 oz serving = 248 grams. Education level "Low" 0 to 6 years of education; "Medium" >6 years to 12 years of education; and "High" >12 years of education. Source data are provided as Source Data file 2.

† In prior GDD reports, the region Central or Eastern Europe and Central Asia was referred as Former Soviet Union, and Southeast and East Asia was referred as Asia.

Oz, ounces; SSBs, sugar-sweetened beverages; UIs, uncertainty intervals.

Supplementary Table 12. Global and regional mean (95% UI) sugar-sweetened beverage intakes (8 oz serving/week) in adults (20+ years) by age, sex, education, and area of residence across 185 countries in 2005.

	World	Central or Eastern Europe and Central Asia†	High-Income Countries	Latin America and Caribbean	Middle East and North Africa	South Asia†	Southeast and East Asia	Sub-Saharan Africa
	mean (95% UI)	*mean (95% UI)*	*mean (95% UI)*	*mean (95% UI)*	*mean (95% UI)*	*mean (95% UI)*	*mean (95% UI)*	*mean (95% UI)*
Overall	2.6 (2.4-2.7)	2.2 (1.9-2.5)	4.7 (4.5-4.9)	7.4 (6.7-8.1)	4.5 (3.8-5.2)	0.8 (0.6-1.2)	0.8 (0.8-1.0)	5.0 (3.9-6.4)
Sex								
Female	2.5 (2.3-2.6)	2.0 (1.7-2.3)	4.2 (4.0-4.4)	7.0 (6.4-7.8)	4.4 (3.8-5.2)	0.8 (0.5-1.2)	0.8 (0.7-1.0)	4.8 (3.8-6.2)
Male	2.7 (2.5-2.9)	2.4 (2.0-2.7)	5.2 (5.0-5.5)	7.7 (7.0-8.6)	4.6 (3.9-5.4)	0.8 (0.6-1.2)	0.9 (0.8-1.0)	5.1 (4.0-6.6)
Age								
20-24	3.9 (3.7-4.2)	4.2 (3.7-4.8)	8.3 (7.9-8.7)	9.9 (9.1-10.8)	6.9 (6.0-8.0)	1.1 (0.8-1.5)	1.7 (1.6-2.0)	5.4 (4.2-6.9)
25-29	3.5 (3.3-3.8)	3.4 (2.9-3.9)	7.5 (7.1-7.8)	9.2 (8.4-10.2)	5.7 (4.8-6.7)	1.0 (0.7-1.4)	1.4 (1.2-1.6)	5.6 (4.4-7.2)
30-34	3.0 (2.8-3.2)	2.7 (2.3-3.2)	6.5 (6.2-6.9)	8.4 (7.6-9.5)	4.7 (4.0-5.6)	0.9 (0.6-1.3)	1.0 (0.9-1.1)	5.5 (4.3-7.3)
35-39	2.6 (2.4-2.8)	2.3 (1.9-2.7)	5.7 (5.4-6.1)	7.7 (6.9-8.6)	4.0 (3.3-4.8)	0.8 (0.5-1.2)	0.8 (0.7-0.9)	5.3 (4.0-7.0)
40-44	2.4 (2.2-2.6)	1.9 (1.6-2.3)	5.1 (4.8-5.4)	6.9 (6.2-7.9)	3.5 (2.9-4.2)	0.7 (0.5-1.2)	0.7 (0.6-0.8)	5.0 (3.8-6.7)
45-49	2.2 (2.0-2.3)	1.7 (1.4-1.9)	4.5 (4.3-4.8)	6.3 (5.6-7.1)	3.1 (2.6-3.8)	0.7 (0.5-1.1)	0.6 (0.5-0.7)	4.6 (3.5-6.2)
50-54	1.9 (1.8-2.0)	1.5 (1.3-1.7)	3.9 (3.7-4.1)	5.8 (5.1-6.5)	3.0 (2.5-3.6)	0.6 (0.4-0.9)	0.5 (0.4-0.6)	4.2 (3.2-5.7)
55-59	1.8 (1.7-1.9)	1.4 (1.2-1.6)	3.3 (3.1-3.5)	5.3 (4.7-6.0)	2.8 (2.4-3.4)	0.6 (0.4-0.9)	0.5 (0.4-0.5)	3.8 (2.8-5.3)
60-64	1.6 (1.5-1.8)	1.2 (1.1-1.5)	2.8 (2.7-2.9)	4.9 (4.3-5.6)	2.8 (2.3-3.5)	0.6 (0.4-0.9)	0.4 (0.4-0.5)	3.5 (2.5-5.0)
65-69	1.5 (1.4-1.6)	1.2 (1.0-1.4)	2.4 (2.2-2.5)	4.6 (4.0-5.3)	2.8 (2.2-3.4)	0.5 (0.3-0.9)	0.4 (0.3-0.4)	3.3 (2.2-4.8)
70-74	1.4 (1.3-1.5)	1.1 (0.9-1.4)	2.1 (2.0-2.2)	4.3 (3.7-5.1)	2.7 (2.2-3.3)	0.5 (0.3-0.9)	0.4 (0.3-0.4)	3.0 (2.0-4.6)
75-79	1.3 (1.2-1.4)	1.0 (0.8-1.3)	1.9 (1.8-2.0)	4.0 (3.4-4.9)	2.7 (2.1-3.4)	0.5 (0.3-0.9)	0.4 (0.3-0.4)	2.8 (1.8-4.6)
80-84	1.3 (1.2-1.4)	0.9 (0.7-1.2)	1.7 (1.6-1.8)	3.9 (3.2-4.7)	2.7 (2.1-3.5)	0.5 (0.3-1.0)	0.4 (0.3-0.4)	2.7 (1.6-4.5)
85+	1.3 (1.2-1.4)	0.9 (0.6-1.2)	1.5 (1.4-1.7)	3.7 (3.0-4.6)	2.7 (2.0-3.6)	0.5 (0.3-1.0)	0.4 (0.3-0.4)	2.5 (1.5-4.4)
Education years								
0-6	2.1 (1.9-2.4)	2.4 (1.9-3.1)	4.1 (3.8-4.3)	6.9 (6.1-7.8)	5.2 (4.2-6.4)	0.5 (0.3-0.8)	0.8 (0.7-1.0)	3.5 (2.6-4.8)
>6-12	2.5 (2.3-2.7)	2.3 (1.9-2.7)	4.5 (4.2-4.7)	7.4 (6.6-8.2)	4.5 (3.8-5.3)	0.9 (0.6-1.3)	0.7 (0.7-0.9)	6.5 (5.1-8.5)
>12	2.9 (2.8-3.1)	2.1 (1.8-2.4)	4.8 (4.6-5.1)	7.7 (6.7-8.8)	3.7 (3.2-4.3)	1.2 (0.8-1.8)	1.0 (0.9-1.1)	7.4 (5.6-9.8)
Area of residence								
Rural	1.8 (1.6-1.9)	2.3 (1.9-2.6)	4.6 (4.4-4.8)	7.0 (6.2-7.9)	4.9 (4.0-6.1)	0.4 (0.3-0.5)	0.7 (0.7-0.9)	4.0 (3.1-5.2)
Urban	3.4 (3.2-3.6)	2.1 (1.8-2.4)	4.7 (4.5-5.0)	7.5 (6.8-8.2)	4.2 (3.6-4.9)	1.8 (1.2-2.9)	1.0 (0.9-1.1)	6.8 (5.2-8.9)

*Data are the mean intakes (95% uncertainty interval) in 8 oz servings per week. All intakes are reported adjusted to 2,000 kcal/d for ages 11 to 74 years, and 1,700 kcal/d for ages 75+ years. Data are based on a Bayesian model that incorporated up to 451 individual-level dietary surveys, and additional survey-level and country-level covariates, to estimate dietary consumption levels. Total sugar-sweetened beverages (SSBs) intake was defined as any beverage with added sugars having ≥50 kcal per 8 oz serving, including commercial or homemade beverages, soft drinks, energy drinks, fruit drinks, punch, lemonade, and aguas frescas. This definition excludes 100% fruit and vegetable juices and non-caloric artificially sweetened drinks. Standardized serving size used for this analysis: 8 oz serving = 248 grams. Education level "Low" 0 to 6 years of education; "Medium" >6 years to 12 years of education; and "High" >12 years of education. Source data are provided as Source Data file 2.

† In prior GDD reports, the region Central or Eastern Europe and Central Asia was referred as Former Soviet Union, and Southeast and East Asia was referred as Asia.

Oz, ounces; SSBs, sugar-sweetened beverages; UIs, uncertainty intervals.

Supplementary Table 13. National mean (95% UI) sugar-sweetened beverage intakes (8 oz servings/week) in adults (20+ years) by sex, age, education, and area of residence in the 25 most populous countries in 1990.

	Sex		Age category			Education			Area of residence	
	Female	Male	20-39 years	40-59 years	60+ years	≤6 years	>6-12 years	>12 years	Rural	Urban
Country	*mean (95% UI)*	*mean (95% UI)*	*mean (95% UI)*	*mean (95% UI)*	*mean (95% UI)*	*mean (95% UI)*	*mean (95% UI)*	*mean (95% UI)*	*mean (95% UI)*	*mean (95% UI)*
China	0.3 (0.3-0.4)	0.3 (0.3-0.4)	0.5 (0.4-0.6)	0.2 (0.2-0.3)	0.1 (0.1-0.2)	0.3 (0.2-0.4)	0.4 (0.3-0.5)	0.4 (0.3-0.5)	0.3 (0.3-0.4)	0.3 (0.3-0.4)
India	0.1 (0.1-0.2)	0.2 (0.1-0.3)	0.2 (0.1-0.3)	0.1 (0.1-0.2)	0.1 (0.1-0.2)	0.1 (0.1-0.2)	0.2 (0.1-0.3)	0.3 (0.2-0.4)	0.1 (0.1-0.1)	0.4 (0.2-0.6)
United States	5.1 (4.8-5.5)	6.5 (6.1-6.9)	8.0 (7.5-8.4)	4.9 (4.6-5.2)	2.7 (2.5-2.9)	7.5 (6.9-8.1)	7.3 (6.9-7.9)	5.4 (5.1-5.7)	6.0 (5.7-6.3)	5.7 (5.4-6.1)
Indonesia	0.5 (0.3-0.6)	0.5 (0.4-0.7)	0.6 (0.5-0.8)	0.3 (0.2-0.4)	0.2 (0.1-0.2)	0.4 (0.3-0.6)	0.5 (0.4-0.7)	0.5 (0.4-0.7)	0.5 (0.4-0.6)	0.5 (0.4-0.7)
Brazil	7.2 (6.3-8.5)	8.0 (6.9-9.4)	9.0 (7.9-10.2)	6.2 (5.4-7.1)	4.5 (3.9-5.2)	7.4 (6.4-8.5)	7.9 (6.9-9.1)	8.0 (6.7-9.4)	6.9 (6.0-8.1)	7.9 (7.0-9.0)
Pakistan	4.2 (2.5-7.2)	4.2 (2.6-7.0)	4.8 (3.1-7.8)	3.6 (2.1-6.4)	2.8 (1.5-5.1)	3.1 (1.8-5.4)	5.7 (3.4-9.8)	7.6 (4.4-13.4)	2.0 (1.3-3.2)	9.2 (5.4-16.1)
Russia	1.0 (0.8-1.2)	1.2 (1.0-1.5)	1.6 (1.3-1.9)	0.8 (0.6-1.0)	0.6 (0.4-0.7)	1.0 (0.8-1.4)	1.2 (0.9-1.4)	1.1 (0.9-1.3)	1.2 (0.9-1.4)	1.1 (0.9-1.3)
Japan	2.3 (1.9-2.7)	2.8 (2.4-3.3)	4.1 (3.5-4.7)	1.9 (1.7-2.2)	1.1 (1.0-1.3)	2.2 (1.8-2.6)	2.6 (2.2-3.0)	2.6 (2.2-3.0)	2.5 (2.1-2.9)	2.6 (2.2-3.0)
Bangladesh	0.2 (0.1-0.3)	0.2 (0.1-0.3)	0.2 (0.1-0.3)	0.2 (0.1-0.2)	0.1 (0.1-0.2)	0.1 (0.1-0.2)	0.2 (0.2-0.4)	0.3 (0.2-0.5)	0.1 (0.1-0.2)	0.5 (0.3-0.8)
Nigeria	0.5 (0.3-0.8)	0.5 (0.3-0.8)	0.5 (0.3-0.8)	0.4 (0.3-0.7)	0.3 (0.2-0.5)	0.4 (0.2-0.7)	0.7 (0.4-1.1)	0.8 (0.4-1.3)	0.4 (0.3-0.7)	0.6 (0.4-1.1)
Mexico	9.1 (8.2-10.2)	10.4 (9.3-11.7)	11.5 (10.5-12.6)	7.9 (7.2-8.7)	5.6 (4.9-6.5)	9.3 (8.3-10.4)	10.0 (8.9-11.2)	10.1 (8.7-11.7)	8.9 (8.0-9.9)	10.1 (9.2-11.2)
Germany	2.3 (2.0-2.7)	3.3 (2.9-3.8)	4.1 (3.6-4.6)	2.4 (2.2-2.7)	1.3 (1.2-1.5)	3.2 (2.8-3.6)	3.1 (2.8-3.5)	2.3 (2.0-2.6)	2.9 (2.6-3.3)	2.8 (2.5-3.1)
Vietnam	1.3 (0.9-1.7)	1.3 (1.0-1.8)	1.7 (1.3-2.3)	0.8 (0.6-1.0)	0.5 (0.3-0.6)	1.2 (0.9-1.6)	1.4 (1.1-1.9)	1.4 (1.1-1.9)	1.3 (1.0-1.7)	1.4 (1.0-1.8)
Philippines	4.0 (3.5-4.7)	3.5 (2.9-4.4)	4.8 (4.2-5.6)	2.2 (1.9-2.6)	1.3 (1.1-1.6)	3.3 (2.8-3.9)	4.0 (3.4-4.6)	3.9 (3.4-4.6)	3.7 (3.2-4.3)	3.9 (3.4-4.5)
Egypt	3.3 (2.7-4.0)	2.8 (2.2-3.5)	3.7 (3.0-4.6)	2.2 (1.8-2.7)	2.0 (1.6-2.6)	3.0 (2.4-3.8)	3.3 (2.6-4.0)	2.7 (2.1-3.3)	3.2 (2.6-4.0)	2.8 (2.2-3.4)
Iran	2.8 (2.4-3.2)	2.9 (2.5-3.3)	3.4 (3.0-3.8)	2.0 (1.7-2.3)	1.8 (1.6-2.2)	2.8 (2.5-3.3)	3.1 (2.7-3.5)	2.5 (2.2-2.9)	3.0 (2.6-3.5)	2.6 (2.3-3.0)
Turkey	3.1 (2.3-4.3)	3.2 (2.4-4.4)	3.9 (3.0-5.1)	2.3 (1.7-3.1)	2.1 (1.6-2.9)	3.1 (2.4-4.2)	3.4 (2.6-4.4)	2.8 (2.1-3.7)	3.5 (2.6-4.6)	3.0 (2.3-3.9)
Thailand	0.8 (0.4-1.7)	0.9 (0.4-1.8)	1.1 (0.6-2.4)	0.5 (0.2-1.1)	0.3 (0.2-0.6)	0.8 (0.4-1.7)	1.0 (0.5-2.0)	1.0 (0.5-2.0)	0.8 (0.4-1.8)	0.9 (0.4-1.8)
Ethiopia	3.5 (2.7-4.6)	3.7 (2.8-4.8)	4.0 (3.1-5.1)	3.3 (2.5-4.2)	2.3 (1.5-3.4)	2.9 (2.2-3.8)	4.9 (3.7-6.5)	5.5 (3.9-7.8)	3.3 (2.6-4.3)	5.3 (4.0-6.9)
United Kingdom	4.6 (4.0-5.2)	4.9 (4.4-5.6)	6.9 (6.3-7.6)	4.2 (3.8-4.6)	2.3 (2.1-2.6)	5.1 (4.6-5.7)	5.0 (4.6-5.6)	3.7 (3.3-4.1)	4.9 (4.4-5.5)	4.7 (4.3-5.2)
Italy	1.5 (1.3-1.7)	1.8 (1.6-2.1)	2.4 (2.1-2.7)	1.4 (1.3-1.6)	0.8 (0.7-0.9)	1.8 (1.6-2.0)	1.8 (1.6-2.0)	1.3 (1.1-1.4)	1.7 (1.5-1.9)	1.6 (1.4-1.8)
France	1.7 (1.5-2.0)	2.5 (2.2-2.9)	3.0 (2.7-3.3)	1.8 (1.7-2.0)	1.0 (0.9-1.1)	2.3 (2.1-2.6)	2.3 (2.1-2.6)	1.7 (1.5-1.9)	2.2 (2.0-2.4)	2.1 (1.9-2.3)
Korea	0.9 (0.8-0.9)	1.3 (1.2-1.4)	1.4 (1.3-1.5)	0.6 (0.6-0.7)	0.4 (0.3-0.4)	0.9 (0.8-1.0)	1.1 (1.0-1.2)	1.1 (1.0-1.2)	1.0 (0.9-1.1)	1.1 (1.0-1.2)
Spain	2.4 (2.0-3.0)	2.8 (2.3-3.4)	3.7 (3.1-4.5)	2.2 (1.9-2.6)	1.3 (1.1-1.5)	2.8 (2.3-3.3)	2.7 (2.3-3.3)	2.0 (1.7-2.4)	2.7 (2.2-3.2)	2.6 (2.2-3.1)
DR Congo	1.4 (0.5-3.8)	1.6 (0.6-4.4)	1.6 (0.6-4.5)	1.3 (0.5-3.6)	0.9 (0.3-2.6)	1.2 (0.4-3.4)	2.1 (0.7-5.7)	2.3 (0.8-6.2)	1.2 (0.4-3.4)	2.0 (0.7-5.5)

*Data are mean intakes (95% uncertainty interval) in 8 oz servings per week. Standardized serving size used for this analysis: 8 oz serving = 248 grams. Countries are ordered top to bottom from most to least populous based on 2018 adult (20+ years) population data. Source data are provided as Source Data file 8.

Supplementary Table 14. National mean (95% UI) sugar-sweetened beverage intakes (8 oz servings/week) in adults (20+ years) by sex, age, education, and area of residence in the 25 most populous countries in 2005.

	Sex		Age category			Education			Area of residence	
	Female	Male	20-39 years	40-59 years	60+ years	≤6 years	>6-12 years	>12 years	Rural	Urban
Country	*mean (95% UI)*	*mean (95% UI)*	*mean (95% UI)*	*mean (95% UI)*	*mean (95% UI)*	*mean (95% UI)*	*mean (95% UI)*	*mean (95% UI)*	*mean (95% UI)*	*mean (95% UI)*
China	0.3 (0.2-0.3)	0.3 (0.2-0.3)	0.4 (0.3-0.5)	0.2 (0.1-0.2)	0.1 (0.1-0.1)	0.2 (0.2-0.3)	0.3 (0.2-0.3)	0.3 (0.2-0.3)	0.3 (0.2-0.3)	0.3 (0.2-0.3)
India	0.2 (0.1-0.3)	0.2 (0.1-0.4)	0.2 (0.1-0.4)	0.2 (0.1-0.3)	0.1 (0.1-0.2)	0.1 (0.1-0.2)	0.2 (0.1-0.3)	0.3 (0.2-0.5)	0.1 (0.1-0.1)	0.4 (0.3-0.7)
United States	6.9 (6.5-7.4)	8.6 (8.1-9.2)	11.1 (10.5-11.8)	6.8 (6.4-7.2)	3.7 (3.5-4.0)	10.1 (9.4-11.0)	10.0 (9.3-10.7)	7.3 (6.9-7.8)	8.0 (7.6-8.4)	7.6 (7.2-8.2)
Indonesia	0.7 (0.5-0.9)	0.8 (0.6-1.0)	1.0 (0.7-1.3)	0.5 (0.3-0.6)	0.3 (0.2-0.4)	0.6 (0.5-0.9)	0.8 (0.6-1.0)	0.8 (0.6-1.0)	0.7 (0.5-0.9)	0.8 (0.6-1.0)
Brazil	4.3 (3.7-5.0)	4.8 (4.1-5.6)	5.5 (4.8-6.2)	3.8 (3.3-4.3)	2.7 (2.3-3.2)	4.3 (3.7-5.0)	4.6 (4.0-5.3)	4.6 (3.9-5.4)	4.1 (3.5-4.7)	4.6 (4.1-5.2)
Pakistan	5.2 (3.2-8.8)	5.2 (3.2-8.8)	5.9 (3.8-9.7)	4.5 (2.6-8.0)	3.4 (1.9-6.2)	3.3 (1.9-5.7)	6.0 (3.6-10.4)	8.0 (4.7-14.2)	2.3 (1.5-3.7)	10.7 (6.5-18.6)
Russia	2.2 (1.7-2.7)	2.7 (2.2-3.3)	3.7 (3.0-4.4)	1.8 (1.5-2.2)	1.2 (1.0-1.6)	2.4 (1.8-3.2)	2.6 (2.1-3.3)	2.4 (2.0-2.9)	2.6 (2.1-3.1)	2.4 (1.9-2.9)
Japan	2.0 (1.7-2.3)	2.4 (2.0-2.9)	3.8 (3.3-4.4)	1.7 (1.5-2.0)	1.0 (0.9-1.2)	1.9 (1.6-2.2)	2.2 (1.9-2.6)	2.2 (1.9-2.5)	2.1 (1.8-2.5)	2.2 (1.9-2.5)
Bangladesh	0.3 (0.2-0.4)	0.3 (0.2-0.4)	0.3 (0.2-0.5)	0.2 (0.2-0.4)	0.2 (0.1-0.3)	0.2 (0.1-0.3)	0.3 (0.2-0.5)	0.4 (0.2-0.7)	0.1 (0.1-0.2)	0.6 (0.4-1.1)
Nigeria	4.6 (3.0-7.1)	4.9 (3.2-7.4)	5.3 (3.5-7.9)	4.3 (2.8-6.8)	3.1 (1.8-5.3)	3.8 (2.4-6.0)	6.3 (4.1-9.8)	7.0 (4.4-11.3)	3.9 (2.5-6.0)	6.2 (4.0-9.4)
Mexico	8.7 (7.7-9.8)	9.9 (8.8-11.1)	11.1 (10.1-12.3)	7.7 (7.0-8.6)	5.5 (4.7-6.4)	8.7 (7.8-9.8)	9.4 (8.4-10.5)	9.4 (8.2-11.0)	8.4 (7.5-9.3)	9.5 (8.6-10.6)
Germany	2.3 (2.0-2.7)	3.2 (2.8-3.7)	4.2 (3.7-4.7)	2.6 (2.3-2.9)	1.4 (1.3-1.6)	3.4 (3.1-3.9)	3.4 (3.0-3.8)	2.5 (2.2-2.8)	2.9 (2.5-3.2)	2.7 (2.4-3.0)
Vietnam	1.8 (1.4-2.3)	1.9 (1.4-2.5)	2.5 (2.0-3.2)	1.2 (0.9-1.5)	0.7 (0.5-0.9)	1.6 (1.3-2.1)	1.9 (1.5-2.5)	1.9 (1.5-2.5)	1.8 (1.4-2.3)	1.9 (1.5-2.4)
Philippines	3.7 (3.1-4.2)	3.2 (2.6-4.0)	4.6 (4.0-5.3)	2.1 (1.8-2.4)	1.3 (1.1-1.5)	3.0 (2.5-3.5)	3.5 (3.0-4.1)	3.5 (3.0-4.1)	3.4 (2.9-3.9)	3.5 (3.0-4.1)
Egypt	3.0 (2.5-3.7)	2.6 (2.0-3.2)	3.4 (2.8-4.2)	2.0 (1.6-2.5)	1.9 (1.4-2.4)	2.9 (2.3-3.6)	3.1 (2.5-3.8)	2.5 (2.0-3.1)	3.0 (2.4-3.7)	2.6 (2.1-3.2)
Iran	2.8 (2.4-3.2)	2.9 (2.5-3.3)	3.4 (3.0-3.8)	2.0 (1.8-2.3)	1.8 (1.5-2.2)	2.9 (2.5-3.3)	3.1 (2.8-3.6)	2.6 (2.2-3.0)	3.1 (2.7-3.6)	2.7 (2.4-3.0)
Turkey	2.1 (1.6-2.9)	2.2 (1.7-3.0)	2.7 (2.1-3.5)	1.6 (1.2-2.1)	1.5 (1.1-2.0)	2.1 (1.6-2.9)	2.3 (1.8-3.0)	1.9 (1.4-2.5)	2.4 (1.8-3.2)	2.1 (1.6-2.7)
Thailand	2.7 (1.3-5.9)	3.0 (1.5-6.1)	4.2 (2.0-8.6)	2.0 (1.0-4.1)	1.2 (0.6-2.4)	2.6 (1.3-5.3)	3.1 (1.5-6.4)	3.1 (1.5-6.3)	2.8 (1.4-5.9)	3.0 (1.5-6.1)
Ethiopia	5.5 (4.3-7.2)	5.9 (4.5-7.7)	6.3 (5.0-8.0)	5.2 (4.0-6.6)	3.6 (2.5-5.5)	4.3 (3.4-5.6)	7.3 (5.5-9.6)	8.1 (5.9-11.4)	5.2 (4.1-6.7)	8.3 (6.4-10.8)
United Kingdom	3.9 (3.4-4.4)	4.1 (3.7-4.6)	5.9 (5.3-6.5)	3.6 (3.3-4.0)	2.0 (1.8-2.2)	4.7 (4.3-5.3)	4.6 (4.2-5.1)	3.4 (3.1-3.8)	4.1 (3.7-4.6)	3.9 (3.6-4.4)
Italy	1.4 (1.2-1.6)	1.8 (1.6-2.0)	2.4 (2.1-2.7)	1.5 (1.3-1.6)	0.8 (0.7-0.9)	1.8 (1.6-2.0)	1.8 (1.6-2.0)	1.3 (1.2-1.5)	1.6 (1.5-1.8)	1.6 (1.4-1.7)
France	2.0 (1.8-2.3)	2.9 (2.6-3.3)	3.6 (3.3-4.0)	2.2 (2.0-2.4)	1.2 (1.1-1.3)	2.9 (2.6-3.2)	2.8 (2.6-3.1)	2.1 (1.9-2.3)	2.5 (2.3-2.8)	2.4 (2.2-2.7)
Korea	0.8 (0.7-0.9)	1.2 (1.1-1.3)	1.5 (1.4-1.6)	0.7 (0.7-0.8)	0.4 (0.4-0.5)	0.9 (0.8-0.9)	1.0 (0.9-1.1)	1.0 (0.9-1.1)	1.0 (0.9-1.1)	1.0 (0.9-1.1)
Spain	2.7 (2.2-3.3)	3.1 (2.5-3.8)	4.2 (3.5-4.9)	2.6 (2.1-3.0)	1.4 (1.2-1.7)	3.3 (2.8-3.9)	3.2 (2.7-3.9)	2.4 (2.0-2.8)	3.0 (2.5-3.6)	2.9 (2.4-3.4)
DR Congo	1.7 (0.6-4.8)	2.0 (0.7-5.4)	2.0 (0.7-5.6)	1.7 (0.6-4.5)	1.2 (0.4-3.3)	1.5 (0.5-4.2)	2.5 (0.9-6.9)	2.8 (1.0-7.6)	1.5 (0.5-4.2)	2.4 (0.9-6.6)

*Data are mean intakes (95% uncertainty interval) in 8 oz servings per week. Standardized serving size used for this analysis: 8 oz serving = 248 grams. Countries are ordered top to bottom from most to least populous based on 2018 adult (20+ years) population data. Source data are provided as Source Data file 8.

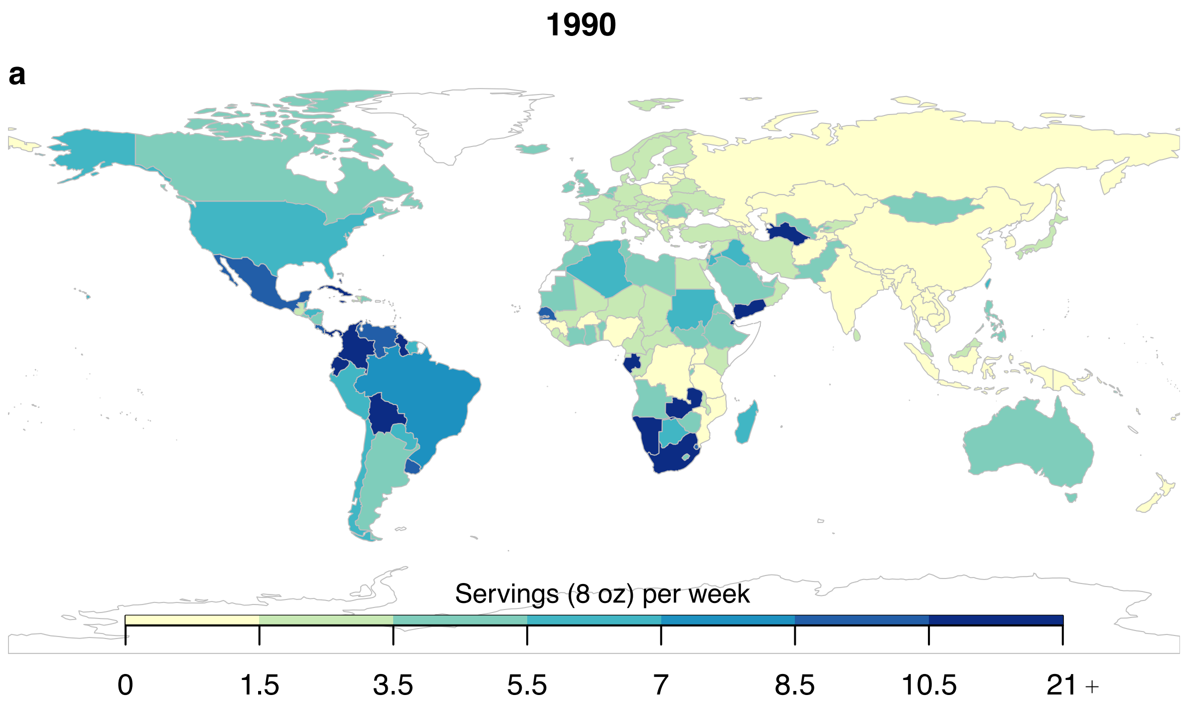

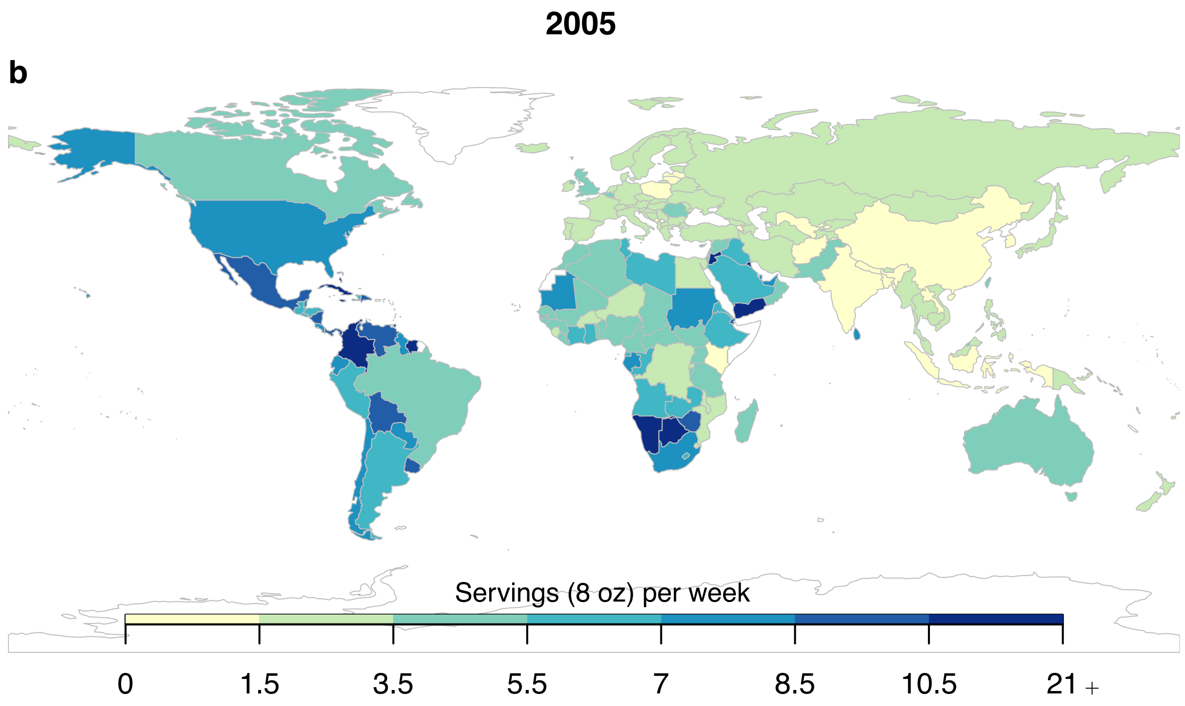

Supplementary Figure 3. **National mean sugar-sweetened beverage intakes (8 oz servings/week) in adults (20+ years) across 185 countries in 1990 (a) and 2005 (b).** SSBs were defined as any beverage with added sugars and ≥50 kcal per 8 oz serving, including commercial or homemade beverages, soft drinks, energy drinks, fruit drinks, punch, lemonade, and aguas frescas. This definition excludes 100% fruit and vegetable juices, non-caloric artificially sweetened drinks, and sweetened milks. The standardized serving size used for this analysis is 8 oz serving (248 grams). For this visual representation, values were truncated at 21 servings/week to better reflect the distribution of intakes globally. The analysis of the data was done using the rworldmap package (v1.3-6). Source data are provided as Source Data file 1.

Oz, ounces; SSBs, sugar-sweetened beverages.

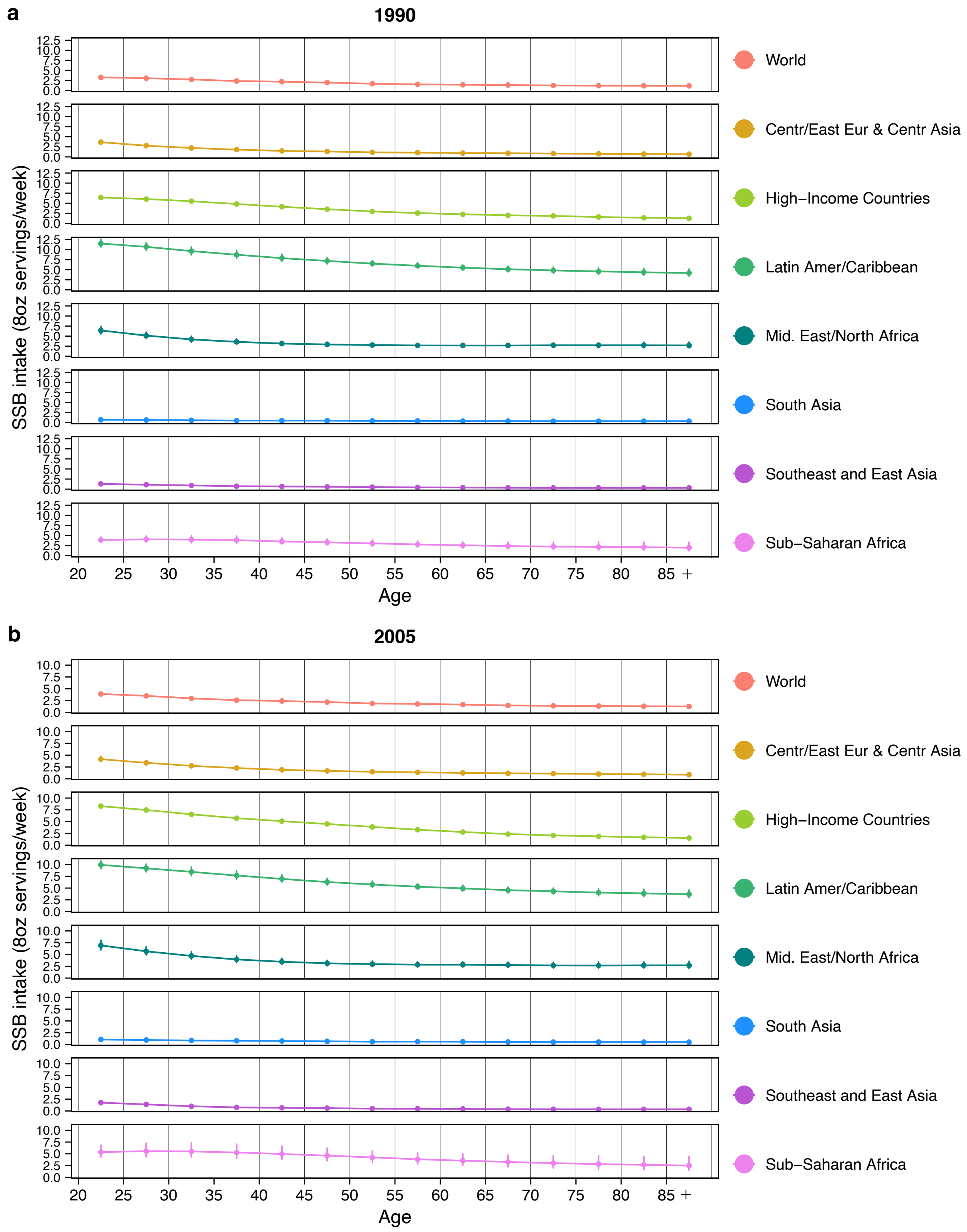

Supplementary Figure 4. **Global and regional sugar-sweetened beverage intakes (8 oz servings/week) by age in adults (+20 years) in 1990 (a) and 2005 (b).** SSBs were defined as any beverage with added sugars and ≥50 kcal per 8 oz serving, including commercial or homemade beverages, soft drinks, energy drinks, fruit drinks, punch, lemonade, and aguas frescas. This definition excludes 100% fruit and vegetable juices, non-caloric artificially sweetened drinks, and sweetened milks. The standardized serving size used for this analysis is 8 oz serving (248 grams). The filled circles represent the mean SSBs intake (8 oz serving/week) and the error bars the 95% UIs. Age groups are 20-24, 25-29, 30-34, 35-39, 40-44, 45-49, 50-54, 55-59, 60-64, 65-69, 70-74, 75-79, 80-84, 85+ years. In prior GDD reports, the region Central/ Eastern Europe and Central Asia was referred as Former Soviet Union, and Southeast and East Asia was referred as Asia. Source data are provided as Source Data file 2.

GDD, Global Dietary Database; oz, ounces; SSBs, sugar-sweetened beverages; UIs, uncertainty intervals.

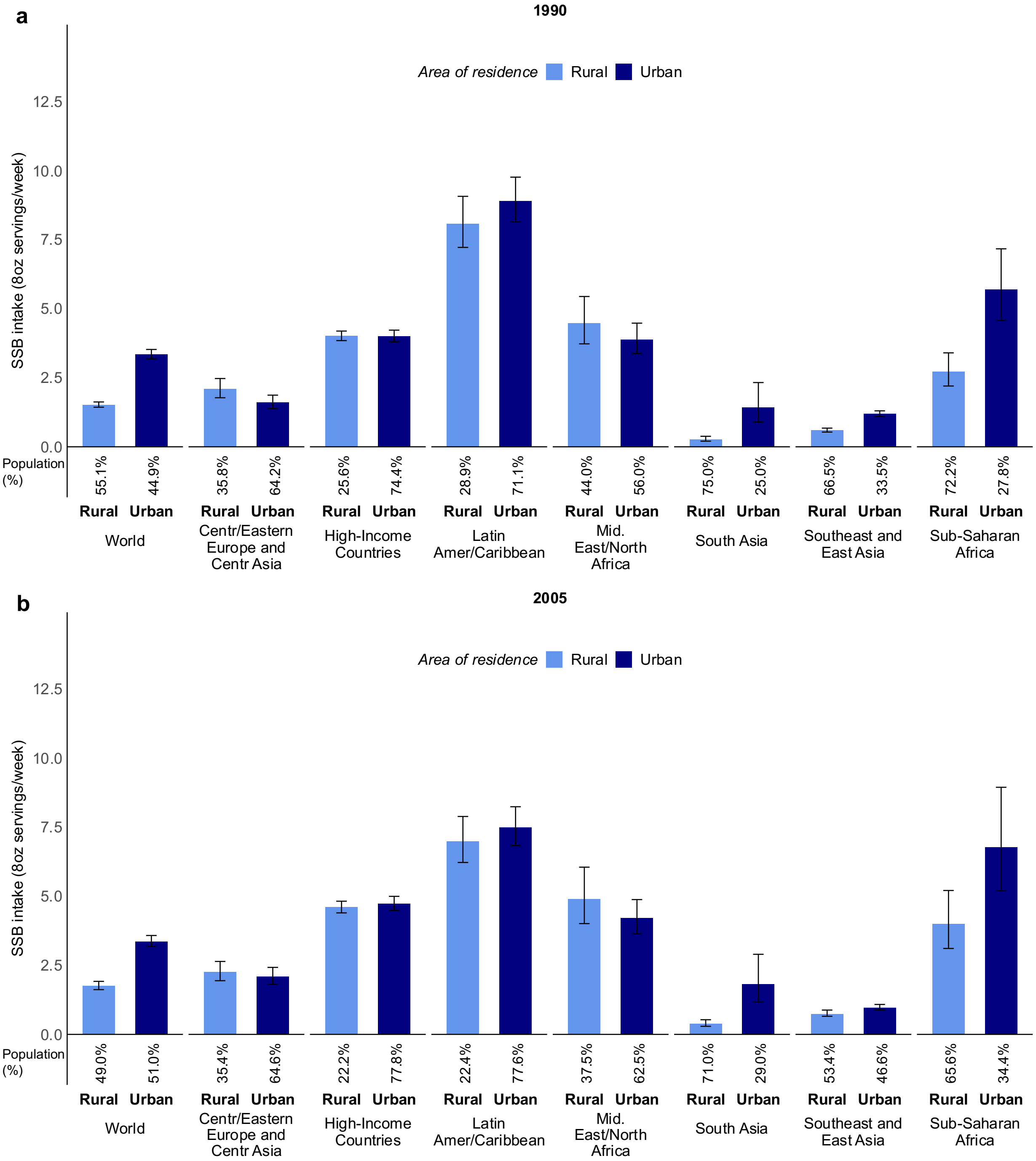

Supplementary Figure 5. **Global and regional sugar-sweetened beverage intakes (8 oz servings/week) in adults (+20 years) by area of residence in 1990 (a) and 2005 (b).** SSBs were defined as any beverage with added sugars having ≥50 kcal per 8 oz serving, including commercial or homemade beverages, soft drinks, energy drinks, fruit drinks, punch, lemonade, and aguas frescas. This definition excludes 100% fruit and vegetable juices, non-caloric artificially sweetened drinks, and sweetened milks. The standardized serving size used for this analysis is 8 oz serving (248 grams).The filled bars represent the mean SSBs intake (8 oz servings/week) and the error bars the 95% UIs. The values below the bars correspond to the percentage (%) of the global population represented in that strata. Colors represent the area of residence as "rural" (light blue) or "urban" (dark blue). In prior GDD reports, the region Central or Eastern Europe and Central Asia was referred as Former Soviet Union, and Southeast and East Asia was referred as Asia. Source data are provided as Source Data file 2.

GDD, Global Dietary Database; oz, ounces; SSBs, sugar-sweetened beverages; UIs, uncertainty intervals.

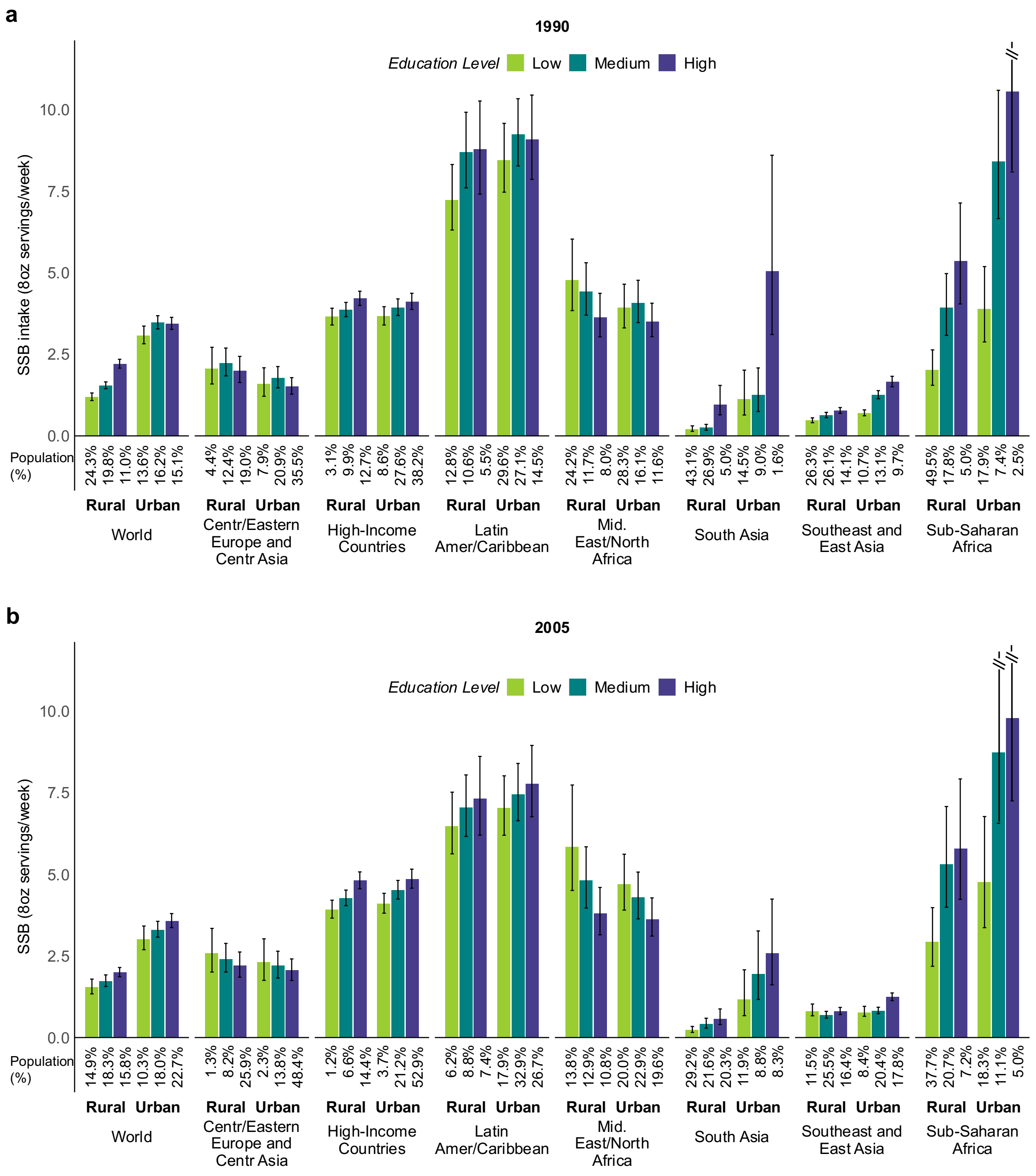

Supplementary Figure 6. **Global and regional sugar-sweetened beverage intakes (8 oz servings/week) in adults (+20 years) by education and area of residence in 1990 (a) and 2005 (b).** SSBs were defined as any beverage with added sugars having ≥50 kcal per 8 oz serving, including commercial or homemade beverages, soft drinks, energy drinks, fruit drinks, punch, lemonade, and aguas frescas. This definition excludes 100% fruit and vegetable juices, non-caloric artificially sweetened drinks, and sweetened milks. The standardized serving size used for this analysis is 8 oz serving (248 grams). The filled bars represent the mean SSBs intake (8 oz servings/week) and the error bars the 95% UIs. Values were truncated at 14.5 (8 oz) servings/week to better reflect the distribution of intakes. Upper 95% UIs above that value are shown with a dashed line. The values below the bars correspond to the percentage (%) of the global population represented in that strata. Colors represent the education level as "low" 0 to 6 years of education (light green), "medium" >6 years to 12 years of education (dark green), or "high" >12 years of education (purple). In prior GDD reports, the region Central or Eastern Europe and Central Asia was referred as Former Soviet Union, and Southeast and East Asia was referred as Asia. Source data are provided as Source Data file 2.

GDD, Global Dietary Database; oz, ounces; SSBs, sugar-sweetened beverages; UIs, uncertainty intervals.

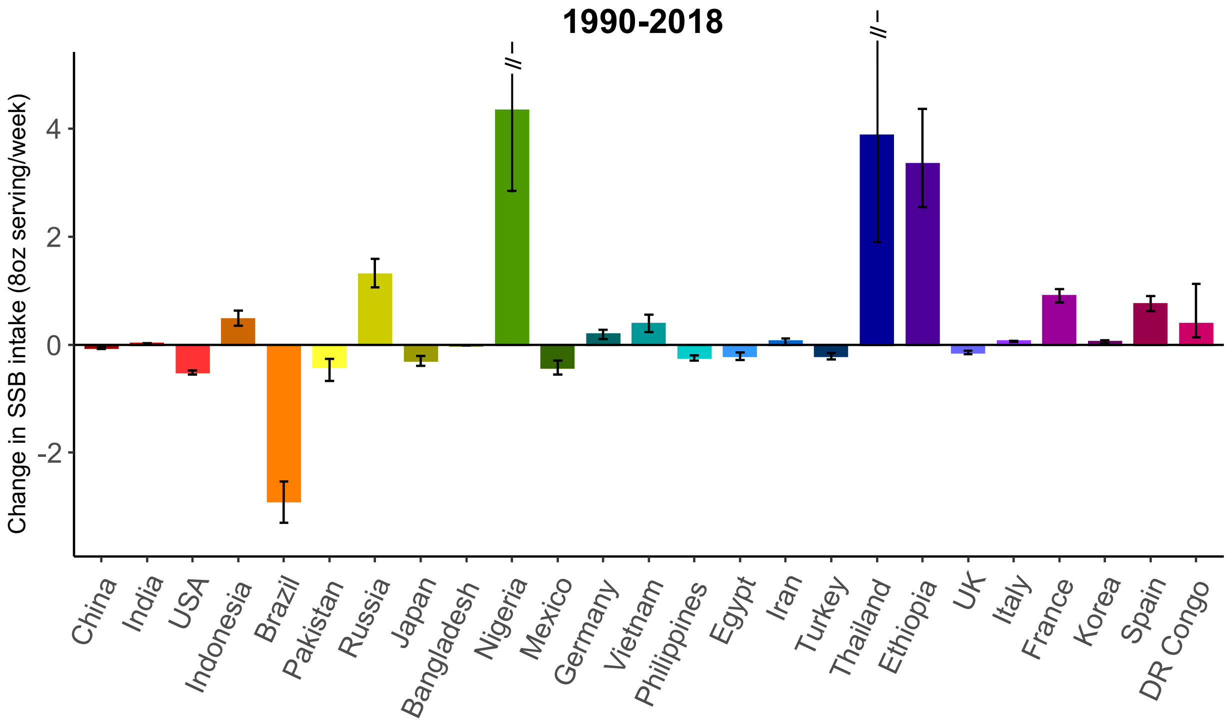

Supplementary Figure 7. **Absolute change in mean sugar-sweetened beverage intakes (8 oz servings/week) in adults (20+ years) in the 25 most populous countries from 1990 to 2018.** SSBs were defined as any beverage with added sugars having ≥50 kcal per 8 oz serving, including commercial or homemade beverages, soft drinks, energy drinks, fruit drinks, punch, lemonade, and aguas frescas. This definition excludes 100% fruit and vegetable juices, non-caloric artificially sweetened drinks, and sweetened milks. The sandardized serving size used for this analysis is 8 oz serving (248 grams). The filled bars represent the mean SSB intakes (8 oz serving/week) and the error bars the 95% UIs. Values were truncated at 5.0 (8 oz) servings/week. Upper 95% UIs above that value are shown with a dashed line. Countries are ordered left to right from most to least populous based on 2018 adult (20+ years) population data. Source data are provided as Source Data file 7.

Oz, ounces; SSBs, sugar-sweetened beverages; UIs, uncertainty intervals.

Supplementary Table 15. Absolute change in mean sugar-sweetened beverage intakes (8 oz servings/week) from 1990-2005, 2005-2018, and 1990-2018 in adults (20+ years) by sex, age education, and area of residence and by world region across 185 countries.

		World	Central/ Eastern Europe and Central Asia†	High-Income Countries	Latin America and Caribbean	Middle East and North Africa	South Asia§	Southeast and East Asia	Sub-Saharan Africa
		mean (95% UI)	*mean (95% UI)*	*mean (95% UI)*	*mean (95% UI)*	*mean (95% UI)*	*mean (95% UI)*	*mean (95% UI)*	*mean (95% UI)*
Sex									
Female	1990-2005	0.20 (0.15,0.27)	0.24 (0.08,0.38)	0.90 (0.83,0.98)	-1.17 (-1.43,-0.93)	0.37 (0.20,0.59)	0.07 (0.05,0.10)	0.08 (0.04,0.14)	0.99 (0.58,1.62)
	2005-2018	0.17 (0.11,0.23)	0.09 (0.02,0.18)	-0.98 (-1.06,-0.90)	0.66 (0.44,0.89)	0.00 (-0.15,0.14)	-0.02 (-0.04,0.01)	0.11 (0.07,0.19)	1.91 (1.45,2.46)
	1990-2018	0.36 (0.28,0.47)	0.33 (0.18,0.48)	-0.07 (-0.09,-0.05)	-0.50 (-0.76,-0.26)	0.37 (0.21,0.56)	0.05 (0.02,0.10)	0.19 (0.12,0.33)	2.91 (2.20,3.81)
Male	1990-2005	0.24 (0.18,0.31)	0.27 (0.08,0.44)	1.14 (1.04,1.24)	-1.29 (-1.57,-1.03)	0.54 (0.34,0.79)	0.06 (0.04,0.09)	0.08 (0.05,0.14)	1.08 (0.64,1.75)
	2005-2018	0.14 (0.08,0.20)	0.12 (0.04,0.23)	-1.24 (-1.34,-1.14)	0.72 (0.49,0.95)	-0.10 (-0.30,0.09)	-0.01 (-0.03,0.02)	0.12 (0.07,0.19)	1.95 (1.47,2.52)
	1990-2018	0.37 (0.28,0.48)	0.39 (0.22,0.57)	-0.10 (-0.13,-0.08)	-0.57 (-0.85,-0.31)	0.44 (0.25,0.68)	0.05 (0.02,0.09)	0.20 (0.13,0.33)	3.05 (2.25,4.02)
Age									
20-39	1990-2005	0.30 (0.23,0.41)	0.28 (-0.01,0.52)	1.63 (1.51,1.77)	-1.47 (-1.77,-1.17)	0.62 (0.39,0.89)	0.06 (0.05,0.09)	0.14 (0.08,0.23)	1.18 (0.73,1.89)
	2005-2018	0.25 (0.17,0.33)	0.14 (0.01,0.31)	-1.75 (-1.89,-1.63)	0.90 (0.62,1.15)	-0.16 (-0.36,0.04)	-0.01 (-0.04,0.02)	0.19 (0.12,0.30)	2.07 (1.60,2.64)
	1990-2018	0.55 (0.43,0.70)	0.42 (0.15,0.66)	-0.12 (-0.15,-0.09)	-0.57 (-0.87,-0.29)	0.46 (0.25,0.72)	0.05 (0.02,0.10)	0.32 (0.22,0.52)	3.28 (2.49,4.26)
40-59	1990-2005	0.17 (0.13,0.23)	0.24 (0.13,0.35)	0.91 (0.83,0.98)	-1.11 (-1.35,-0.88)	0.36 (0.20,0.56)	0.07 (0.05,0.09)	0.06 (0.03,0.11)	0.89 (0.50,1.52)
	2005-2018	0.10 (0.05,0.15)	0.10 (0.05,0.17)	-1.02 (-1.10,-0.95)	0.54 (0.33,0.77)	0.04 (-0.13,0.20)	-0.01 (-0.03,0.01)	0.08 (0.05,0.13)	1.80 (1.35,2.35)
	1990-2018	0.27 (0.20,0.35)	0.35 (0.24,0.46)	-0.12 (-0.14,-0.09)	-0.56 (-0.79,-0.34)	0.39 (0.25,0.61)	0.05 (0.03,0.09)	0.13 (0.08,0.24)	2.71 (1.98,3.66)
60+	1990-2005	0.10 (0.07,0.13)	0.23 (0.16,0.32)	0.48 (0.44,0.53)	-0.83 (-1.03,-0.65)	0.08 (-0.01,0.21)	0.06 (0.04,0.10)	0.02 (0.00,0.05)	0.52 (0.24,1.02)
	2005-2018	0.03 (0.00,0.06)	0.06 (0.03,0.10)	-0.51 (-0.55,-0.47)	0.43 (0.27,0.61)	0.15 (0.07,0.24)	-0.01 (-0.02,0.00)	0.04 (0.02,0.08)	1.38 (0.95,1.92)
	1990-2018	0.13 (0.09,0.18)	0.29 (0.22,0.39)	-0.03 (-0.04,-0.01)	-0.39 (-0.58,-0.23)	0.24 (0.14,0.37)	0.05 (0.03,0.09)	0.06 (0.02,0.12)	1.91 (1.28,2.80)
Education									
0-6	1990-2005	0.21 (0.14,0.32)	0.35 (0.18,0.54)	0.73 (0.65,0.81)	-1.16 (-1.45,-0.87)	0.61 (0.40,0.89)	0.03 (0.01,0.05)	0.18 (0.13,0.28)	1.04 (0.67,1.62)
	2005-2018	0.35 (0.27,0.45)	0.11 (0.03,0.21)	-0.47 (-0.52,-0.43)	0.54 (0.29,0.78)	-0.07 (-0.24,0.10)	0.00 (-0.02,0.03)	0.18 (0.11,0.30)	1.67 (1.24,2.20)
	1990-2018	0.57 (0.44,0.73)	0.46 (0.30,0.64)	0.26 (0.21,0.31)	-0.62 (-0.89,-0.36)	0.55 (0.34,0.80)	0.03 (0.00,0.07)	0.37 (0.25,0.57)	2.72 (2.02,3.63)
>6-12	1990-2005	0.10 (0.05,0.18)	0.17 (0.01,0.31)	0.70 (0.63,0.78)	-1.40 (-1.66,-1.14)	0.24 (0.02,0.51)	0.06 (0.04,0.08)	0.04 (0.02,0.09)	1.18 (0.58,2.08)
	2005-2018	0.21 (0.16,0.27)	0.19 (0.10,0.30)	-0.65 (-0.71,-0.59)	0.83 (0.60,1.07)	0.05 (-0.20,0.30)	-0.01 (-0.03,0.01)	0.08 (0.05,0.13)	2.50 (1.91,3.21)
	1990-2018	0.31 (0.23,0.41)	0.36 (0.21,0.52)	0.05 (0.03,0.08)	-0.57 (-0.84,-0.32)	0.29 (0.09,0.56)	0.05 (0.03,0.08)	0.12 (0.07,0.22)	3.70 (2.69,4.97)
>12	1990-2005	0.39 (0.34,0.45)	0.28 (0.09,0.44)	1.30 (1.20,1.40)	-1.04 (-1.34,-0.76)	0.33 (0.16,0.58)	0.44 (0.32,0.60)	-0.03 (-0.06,0.01)	0.52 (-0.09,1.41)
	2005-2018	-0.31 (-0.35,-0.26)	0.05 (-0.02,0.16)	-1.56 (-1.68,-1.44)	0.75 (0.49,1.01)	-0.15 (-0.34,0.04)	-0.15 (-0.24,-0.08)	0.06 (0.04,0.10)	2.34 (1.72,3.05)
	1990-2018	0.09 (0.03,0.14)	0.34 (0.15,0.50)	-0.26 (-0.28,-0.24)	-0.29 (-0.58,-0.02)	0.19 (0.03,0.39)	0.29 (0.18,0.45)	0.03 (-0.02,0.10)	2.89 (1.95,4.06)
Area of residence									
Rural	1990-2005	0.24 (0.19,0.32)	0.03 (-0.19,0.20)	0.99 (0.92,1.08)	-1.02 (-1.26,-0.80)	0.45 (0.25,0.68)	0.05 (0.04,0.06)	0.15 (0.11,0.23)	0.99 (0.66,1.46)
	2005-2018	0.29 (0.23,0.36)	0.24 (0.13,0.39)	-1.05 (-1.13,-0.98)	0.95 (0.73,1.21)	0.03 (-0.07,0.13)	0.00 (-0.01,0.01)	0.14 (0.09,0.23)	1.85 (1.45,2.33)
	1990-2018	0.53 (0.44,0.65)	0.27 (0.06,0.45)	-0.06 (-0.08,-0.04)	-0.07 (-0.30,0.17)	0.48 (0.29,0.70)	0.05 (0.03,0.07)	0.29 (0.21,0.45)	2.86 (2.21,3.64)
Urban	1990-2005	0.20 (0.14,0.27)	0.38 (0.23,0.52)	1.02 (0.94,1.11)	-1.28 (-1.51,-1.06)	0.46 (0.28,0.69)	0.10 (0.07,0.15)	0.03 (0.00,0.08)	1.12 (0.52,2.06)
	2005-2018	0.04 (-0.01,0.10)	0.04 (-0.02,0.11)	-1.12 (-1.21,-1.03)	0.63 (0.42,0.83)	-0.09 (-0.26,0.08)	-0.03 (-0.08,0.02)	0.09 (0.06,0.15)	2.05 (1.46,2.75)
	1990-2018	0.24 (0.16,0.33)	0.41 (0.28,0.55)	-0.10 (-0.12,-0.08)	-0.65 (-0.89,-0.43)	0.37 (0.21,0.58)	0.07 (0.01,0.15)	0.12 (0.06,0.23)	3.18 (2.25,4.42)

*Data are mean absolute change in intakes (95% uncertainty interval) in 8 oz servings per week. Standardized serving size used for this analysis: 8 oz serving = 248 grams. †Referred to as Former Soviet Union in previous Global Dietary Database reports. §Referred to as Asia in previous Global Dietary Database reports. Source data are provided as Source Data file 6.

Supplementary Discussion 1. Trends over time by age, sex, education, and urbanicity within the 25 most populous countries

Within the 25 most populous countries, trends were similar between males and females (Supplementary Table 18). By age, from 1990 to 2018 the largest increases were among adults age 20-39 years in Thailand (+6·33 [3·11, 12·95]; 551.2%) and Nigeria (+4·75 [3·05, 7·35]; 817·0%); while the largest decreases were seen in Brazil among adults age 20-39 years (-3·63 [-4·16, -3·16]; -39.8%) and adults age 40-59 years (-2·50 [-2·88, -2·16]; 39·6%) (Supplementary Table 19). By education, from 1990 to 2018 the largest increases were in Nigeria among high educated (+6·75 [4·07, 10·74]; 815·7%) and medium educated (+6·09 [3·82, 9·39]; 815·9%) adults, and the largest decreases in Brazil among high educated (-3·03 [-3·56, -2·54]; -39·8%), medium educated (-2·99 [-3·45, -2·57]; -39·6%), and low educated (-2·79 [-3·24, -2·40]; -39·8%) adults (Supplementary Table 20). Finally, by area of residence the most noticeable increases in SSB intake between 1990 and 2018 were among urban adults in Nigeria (+5·29 [3·27, 8·36]; 819·8%) and Ethiopia (+4·77 [3·51, 6·37]; 89·5 %), and the largest decreases in urban (-2·95 [-3·36, -2·58]; 39·8%) and rural (-2·59 [-3·04, -2·21]; 39·8%) adults in Brazil (Supplementary Table 21).

Supplementary Table 16. Absolute change in mean sugar-sweetened beverage intakes (8 oz servings/week) in adults (20+ years) from 1990-2005, 2005-2018, and 1990-2018 by sex in the 25 most populous countries.

	Female			Male		
	1990-2005	2005-2018	1990-2018	1990-2005	2005-2018	1990-2018
Country	*mean (95% UI)*	*mean (95% UI)*	*mean (95% UI)*	*mean (95% UI)*	*mean (95% UI)*	*mean (95% UI)*
China	-0.06 (-0.07,-0.04)	0.00 (0.00,0.00)	-0.06 (-0.08,-0.05)	-0.06 (-0.08,-0.04)	0.00 (0.00,0.00)	-0.06 (-0.08,-0.05)
India	0.00 (0.00,0.00)	0.02 (0.01,0.03)	0.01 (0.01,0.03)	0.00 (0.00,0.00)	0.02 (0.01,0.04)	0.02 (0.01,0.04)
United States	2.03 (1.88,2.20)	-2.48 (-2.68,-2.30)	-0.45 (-0.49,-0.42)	2.52 (2.34,2.73)	-3.11 (-3.36,-2.88)	-0.58 (-0.63,-0.54)
Indonesia	0.20 (0.15,0.28)	0.24 (0.17,0.33)	0.44 (0.32,0.61)	0.23 (0.16,0.31)	0.27 (0.19,0.37)	0.50 (0.36,0.68)
Brazil	-2.81 (-3.28,-2.41)	0.07 (0.06,0.08)	-2.74 (-3.20,-2.35)	-3.13 (-3.71,-2.67)	0.08 (0.06,0.09)	-3.06 (-3.61,-2.60)
Pakistan	-0.03 (-0.05,-0.02)	-0.38 (-0.63,-0.23)	-0.42 (-0.68,-0.25)	-0.03 (-0.05,-0.02)	-0.38 (-0.63,-0.23)	-0.41 (-0.68,-0.26)
Russia	1.22 (0.96,1.52)	-0.05 (-0.06,-0.03)	1.17 (0.92,1.47)	1.51 (1.20,1.89)	-0.07 (-0.09,-0.05)	1.44 (1.14,1.81)
Japan	-0.18 (-0.25,-0.13)	-0.09 (-0.12,-0.06)	-0.27 (-0.36,-0.19)	-0.22 (-0.30,-0.15)	-0.10 (-0.15,-0.07)	-0.32 (-0.44,-0.22)
Bangladesh	0.02 (0.01,0.04)	-0.03 (-0.06,-0.02)	-0.01 (-0.02,-0.01)	0.03 (0.01,0.05)	-0.04 (-0.06,-0.02)	-0.01 (-0.02,-0.01)
Nigeria	4.00 (2.53,6.39)	0.19 (-1.53,1.91)	4.18 (2.65,6.59)	4.24 (2.71,6.66)	0.16 (-1.60,1.92)	4.42 (2.80,6.91)
Mexico	-0.41 (-0.55,-0.28)	0.01 (-0.01,0.02)	-0.40 (-0.53,-0.28)	-0.47 (-0.63,-0.32)	0.02 (0.01,0.04)	-0.45 (-0.59,-0.31)
Germany	0.35 (0.22,0.49)	-0.19 (-0.25,-0.13)	0.16 (0.09,0.23)	0.48 (0.30,0.67)	-0.26 (-0.35,-0.18)	0.22 (0.12,0.32)
Vietnam	0.48 (0.29,0.70)	-0.10 (-0.15,-0.07)	0.37 (0.22,0.55)	0.50 (0.31,0.73)	-0.11 (-0.16,-0.08)	0.39 (0.23,0.58)
Philippines	-0.24 (-0.30,-0.20)	-0.01 (-0.02,-0.01)	-0.26 (-0.31,-0.21)	-0.21 (-0.27,-0.16)	-0.01 (-0.01,-0.01)	-0.22 (-0.29,-0.17)
Egypt	-0.20 (-0.29,-0.12)	-0.03 (-0.04,-0.01)	-0.23 (-0.31,-0.15)	-0.16 (-0.24,-0.09)	-0.03 (-0.05,-0.02)	-0.19 (-0.27,-0.13)
Iran	0.10 (0.07,0.13)	-0.04 (-0.08,0.00)	0.06 (0.00,0.12)	0.09 (0.07,0.12)	-0.03 (-0.07,0.00)	0.06 (0.00,0.12)
Turkey	-0.86 (-1.17,-0.64)	0.66 (0.49,0.90)	-0.20 (-0.27,-0.15)	-0.90 (-1.21,-0.67)	0.69 (0.51,0.92)	-0.22 (-0.29,-0.16)
Thailand	1.68 (0.81,3.60)	1.98 (0.95,4.22)	3.65 (1.75,7.81)	1.87 (0.93,3.79)	2.20 (1.08,4.47)	4.08 (2.00,8.25)
Ethiopia	1.62 (1.21,2.15)	1.61 (1.18,2.19)	3.24 (2.40,4.34)	1.72 (1.29,2.27)	1.72 (1.25,2.31)	3.44 (2.54,4.58)
United Kingdom	-0.24 (-0.31,-0.17)	0.10 (0.06,0.14)	-0.14 (-0.17,-0.11)	-0.26 (-0.33,-0.19)	0.10 (0.06,0.14)	-0.16 (-0.19,-0.13)
Italy	0.07 (0.06,0.09)	-0.02 (-0.02,-0.02)	0.05 (0.04,0.07)	0.10 (0.08,0.12)	-0.03 (-0.03,-0.02)	0.07 (0.06,0.09)
France	0.45 (0.38,0.53)	0.29 (0.25,0.34)	0.74 (0.63,0.87)	0.65 (0.55,0.75)	0.42 (0.36,0.50)	1.07 (0.90,1.25)
Korea	0.03 (0.02,0.05)	0.01 (0.00,0.02)	0.04 (0.02,0.06)	0.05 (0.02,0.07)	0.02 (0.01,0.03)	0.06 (0.03,0.10)
Spain	0.47 (0.37,0.58)	0.24 (0.19,0.29)	0.70 (0.56,0.87)	0.53 (0.42,0.65)	0.27 (0.22,0.33)	0.80 (0.64,0.98)
DR Congo	0.27 (0.09,0.79)	0.09 (0.03,0.27)	0.36 (0.12,1.06)	0.30 (0.10,0.90)	0.10 (0.03,0.31)	0.40 (0.14,1.20)

*Data are mean absolute change in intakes (95% uncertainty interval) in 8 oz servings per day. Standardized serving size used for this analysis: 8 oz serving = 248 grams. Countries are ordered top to bottom from most to least populous based on 2018 adult (20+ years) population data. Source data are provided as Source Data file 7.

Supplementary Table 17. Absolute change in mean sugar-sweetened beverage intakes (8 oz servings/week) in adults (20+ years) from 1990-2005, 2005-2018, and 1990-2018 by age in the 25 most populous countries.

	20-39 years			40-59 years			60+ years		
	1990-2005	2005-2018	1990-2018	1990-2005	2005-2018	1990-2018	1990-2005	2005-2018	1990-2018
Country	*mean (95% UI)*	*mean (95% UI)*	*mean (95% UI)*	*mean (95% UI)*	*mean (95% UI)*	*mean (95% UI)*	*mean (95% UI)*	*mean (95% UI)*	*mean (95% UI)*
China	-0.10 (-0.13,-0.08)	0.00 (0.00,0.00)	-0.10 (-0.13,-0.08)	-0.03 (-0.04,-0.03)	-0.01 (-0.01,-0.01)	-0.04 (-0.05,-0.03)	-0.02 (-0.03,-0.02)	0.00 (0.00,0.00)	-0.02 (-0.03,-0.02)
India	0.00 (0.00,0.00)	0.02 (0.01,0.04)	0.02 (0.01,0.04)	0.00 (0.00,0.00)	0.02 (0.01,0.03)	0.01 (0.01,0.03)	0.00 (0.00,0.00)	0.01 (0.01,0.02)	0.01 (0.01,0.02)
United States	3.43 (3.20,3.70)	-4.10 (-4.42,-3.82)	-0.67 (-0.72,-0.62)	2.01 (1.86,2.17)	-2.60 (-2.81,-2.42)	-0.60 (-0.64,-0.56)	1.13 (1.04,1.23)	-1.36 (-1.48,-1.25)	-0.23 (-0.25,-0.21)
Indonesia	0.30 (0.22,0.40)	0.36 (0.27,0.49)	0.66 (0.49,0.88)	0.15 (0.11,0.20)	0.17 (0.12,0.23)	0.32 (0.24,0.43)	0.09 (0.07,0.12)	0.11 (0.08,0.15)	0.19 (0.14,0.26)
Brazil	-3.72 (-4.26,-3.24)	0.09 (0.07,0.11)	-3.63 (-4.16,-3.16)	-2.56 (-2.94,-2.22)	0.06 (0.05,0.07)	-2.50 (-2.88,-2.16)	-1.88 (-2.19,-1.60)	0.06 (0.05,0.07)	-1.82 (-2.12,-1.55)
Pakistan	-0.05 (-0.07,-0.03)	-0.43 (-0.67,-0.27)	-0.48 (-0.75,-0.31)	-0.01 (-0.03,0.01)	-0.33 (-0.59,-0.19)	-0.34 (-0.60,-0.20)	-0.03 (-0.05,-0.02)	-0.25 (-0.46,-0.14)	-0.28 (-0.51,-0.15)
Russia	2.13 (1.75,2.60)	-0.17 (-0.21,-0.14)	1.96 (1.59,2.40)	1.04 (0.83,1.29)	0.00 (-0.01,0.01)	1.04 (0.83,1.29)	0.68 (0.53,0.89)	0.03 (0.02,0.04)	0.71 (0.56,0.93)
Japan	-0.35 (-0.47,-0.24)	-0.20 (-0.27,-0.14)	-0.55 (-0.74,-0.37)	-0.20 (-0.26,-0.15)	-0.03 (-0.06,0.00)	-0.23 (-0.32,-0.15)	-0.11 (-0.14,-0.08)	-0.08 (-0.10,-0.06)	-0.19 (-0.24,-0.14)
Bangladesh	0.03 (0.01,0.05)	-0.04 (-0.07,-0.02)	-0.01 (-0.02,-0.01)	0.02 (0.01,0.04)	-0.03 (-0.06,-0.02)	-0.01 (-0.01,0.00)	0.02 (0.01,0.03)	-0.03 (-0.04,-0.01)	-0.01 (-0.02,0.00)
Nigeria	4.53 (2.90,7.06)	0.18 (-1.63,2.04)	4.75 (3.05,7.35)	3.72 (2.36,6.04)	0.17 (-1.40,1.76)	3.89 (2.39,6.23)	2.66 (1.56,4.63)	0.10 (-1.06,1.26)	2.75 (1.59,4.75)
Mexico	-0.60 (-0.78,-0.42)	0.03 (0.02,0.05)	-0.56 (-0.73,-0.40)	-0.30 (-0.42,-0.19)	-0.03 (-0.05,-0.01)	-0.33 (-0.44,-0.22)	-0.27 (-0.36,-0.19)	0.05 (0.04,0.06)	-0.23 (-0.31,-0.15)
Germany	0.57 (0.33,0.82)	-0.25 (-0.36,-0.14)	0.33 (0.19,0.46)	0.47 (0.32,0.63)	-0.29 (-0.37,-0.22)	0.18 (0.10,0.26)	0.22 (0.14,0.31)	-0.14 (-0.18,-0.10)	0.08 (0.04,0.13)
Vietnam	0.69 (0.42,0.98)	-0.16 (-0.22,-0.11)	0.53 (0.31,0.77)	0.37 (0.24,0.51)	-0.08 (-0.11,-0.06)	0.29 (0.18,0.40)	0.18 (0.11,0.26)	-0.01 (-0.02,0.00)	0.17 (0.11,0.24)
Philippines	-0.32 (-0.39,-0.26)	-0.01 (-0.01,-0.01)	-0.33 (-0.40,-0.27)	-0.14 (-0.17,-0.11)	-0.02 (-0.03,-0.02)	-0.16 (-0.20,-0.13)	-0.08 (-0.10,-0.06)	0.00 (0.00,0.00)	-0.08 (-0.10,-0.06)
Egypt	-0.21 (-0.31,-0.12)	-0.07 (-0.09,-0.05)	-0.28 (-0.38,-0.19)	-0.14 (-0.21,-0.09)	0.02 (0.01,0.02)	-0.13 (-0.18,-0.08)	-0.13 (-0.19,-0.08)	0.02 (0.01,0.03)	-0.11 (-0.17,-0.07)
Iran	0.13 (0.10,0.16)	-0.13 (-0.18,-0.07)	0.00 (-0.07,0.08)	0.06 (0.04,0.08)	0.06 (0.04,0.09)	0.13 (0.08,0.17)	0.04 (0.01,0.08)	0.07 (0.04,0.09)	0.11 (0.06,0.17)
Turkey	-1.15 (-1.51,-0.89)	0.84 (0.65,1.11)	-0.31 (-0.40,-0.24)	-0.66 (-0.88,-0.50)	0.54 (0.41,0.72)	-0.12 (-0.16,-0.09)	-0.62 (-0.83,-0.45)	0.50 (0.37,0.68)	-0.12 (-0.16,-0.09)
Thailand	2.85 (1.40,5.84)	3.48 (1.70,7.11)	6.33 (3.11,12.95)	1.39 (0.68,2.86)	1.52 (0.74,3.14)	2.91 (1.42,5.99)	0.82 (0.40,1.65)	0.96 (0.47,1.98)	1.77 (0.87,3.62)
Ethiopia	1.85 (1.42,2.39)	1.84 (1.36,2.41)	3.69 (2.78,4.80)	1.50 (1.15,1.97)	1.53 (1.15,2.06)	3.03 (2.31,4.03)	1.06 (0.71,1.63)	1.01 (0.65,1.63)	2.07 (1.36,3.27)
United Kingdom	-0.41 (-0.52,-0.30)	0.21 (0.15,0.28)	-0.20 (-0.24,-0.15)	-0.20 (-0.27,-0.14)	0.04 (0.01,0.08)	-0.16 (-0.19,-0.13)	-0.12 (-0.16,-0.09)	0.05 (0.02,0.07)	-0.08 (-0.10,-0.06)
Italy	0.11 (0.09,0.14)	-0.01 (-0.01,0.00)	0.10 (0.08,0.12)	0.11 (0.10,0.13)	-0.04 (-0.04,-0.03)	0.08 (0.06,0.09)	0.04 (0.03,0.04)	-0.02 (-0.03,-0.02)	0.01 (0.01,0.02)
France	0.88 (0.77,1.00)	0.57 (0.49,0.65)	1.45 (1.26,1.65)	0.51 (0.45,0.59)	0.32 (0.28,0.37)	0.84 (0.72,0.96)	0.27 (0.23,0.31)	0.19 (0.17,0.22)	0.46 (0.40,0.53)
Korea	0.04 (0.00,0.07)	0.04 (0.03,0.06)	0.08 (0.03,0.13)	0.05 (0.03,0.07)	-0.01 (-0.02,0.00)	0.04 (0.02,0.06)	0.02 (0.01,0.03)	0.00 (0.00,0.01)	0.03 (0.01,0.04)
Spain	0.73 (0.61,0.87)	0.39 (0.32,0.47)	1.12 (0.92,1.34)	0.53 (0.44,0.64)	0.25 (0.21,0.30)	0.78 (0.65,0.94)	0.23 (0.19,0.28)	0.13 (0.11,0.16)	0.37 (0.30,0.44)
DR Congo	0.32 (0.11,0.93)	0.11 (0.04,0.32)	0.42 (0.15,1.26)	0.26 (0.09,0.77)	0.09 (0.03,0.27)	0.35 (0.12,1.04)	0.18 (0.06,0.55)	0.05 (0.02,0.17)	0.23 (0.07,0.72)

*Data are mean absolute change in intakes (95% uncertainty interval) in 8 oz servings per week. Standardized serving size used for this analysis: 8 oz serving = 248 grams. Countries are ordered top to bottom from most to least populous based on 2018 adult (20+ years) population data. Source data are provided as Source Data file 7.

Supplementary Table 18. Absolute change in mean sugar-sweetened beverage intakes (8 oz servings/week) in adults (20+ years) from 1990-2005, 2005-2018, and 1990-2018 by education level in the 25 most populous countries.

	≤6 years of education			>6-12 years of education			>12 years of education		
	1990-2005	2005-2018	1990-2018	1990-2005	2005-2018	1990-2018	1990-2005	2005-2018	1990-2018
Country	*mean (95% UI)*	*mean (95% UI)*	*mean (95% UI)*	*mean (95% UI)*	*mean (95% UI)*	*mean (95% UI)*	*mean (95% UI)*	*mean (95% UI)*	*mean (95% UI)*
China	-0.05 (-0.07,-0.04)	0.00 (0.00,0.00)	-0.05 (-0.07,-0.04)	-0.06 (-0.08,-0.05)	0.00 (0.00,0.00)	-0.06 (-0.08,-0.05)	-0.06 (-0.08,-0.05)	0.00 (0.00,0.00)	-0.06 (-0.08,-0.05)
India	0.00 (0.00,0.00)	0.01 (0.01,0.03)	0.01 (0.01,0.02)	0.00 (-0.01,0.00)	0.02 (0.01,0.05)	0.02 (0.01,0.04)	0.00 (-0.01,0.00)	0.03 (0.02,0.06)	0.03 (0.02,0.05)
United States	2.92 (2.67,3.20)	-3.58 (-3.92,-3.28)	-0.66 (-0.72,-0.61)	2.87 (2.64,3.13)	-3.53 (-3.84,-3.24)	-0.65 (-0.71,-0.60)	2.11 (1.96,2.28)	-2.59 (-2.79,-2.40)	-0.48 (-0.51,-0.45)
Indonesia	0.20 (0.15,0.26)	0.23 (0.17,0.32)	0.43 (0.32,0.58)	0.24 (0.18,0.32)	0.28 (0.21,0.38)	0.52 (0.39,0.69)	0.24 (0.17,0.32)	0.28 (0.20,0.38)	0.51 (0.38,0.70)
Brazil	-2.86 (-3.32,-2.46)	0.07 (0.06,0.08)	-2.79 (-3.24,-2.40)	-3.07 (-3.54,-2.64)	0.07 (0.06,0.09)	-2.99 (-3.45,-2.57)	-3.11 (-3.64,-2.61)	0.08 (0.06,0.09)	-3.03 (-3.56,-2.54)
Pakistan	-0.03 (-0.04,-0.02)	-0.28 (-0.49,-0.16)	-0.31 (-0.53,-0.18)	-0.05 (-0.08,-0.03)	-0.51 (-0.89,-0.31)	-0.56 (-0.97,-0.34)	-0.06 (-0.10,-0.04)	-0.69 (-1.12,-0.40)	-0.75 (-1.21,-0.44)
Russia	1.31 (0.97,1.76)	-0.05 (-0.07,-0.04)	1.26 (0.93,1.69)	1.44 (1.13,1.82)	-0.06 (-0.08,-0.04)	1.38 (1.08,1.76)	1.32 (1.08,1.61)	-0.05 (-0.07,-0.04)	1.26 (1.03,1.55)
Japan	-0.17 (-0.23,-0.12)	-0.08 (-0.11,-0.05)	-0.25 (-0.35,-0.17)	-0.21 (-0.27,-0.14)	-0.10 (-0.13,-0.06)	-0.30 (-0.40,-0.21)	-0.20 (-0.27,-0.14)	-0.10 (-0.13,-0.06)	-0.30 (-0.40,-0.21)
Bangladesh	0.02 (0.01,0.03)	-0.03 (-0.04,-0.01)	-0.01 (-0.01,0.00)	0.03 (0.02,0.06)	-0.05 (-0.08,-0.03)	-0.01 (-0.02,-0.01)	0.04 (0.02,0.08)	-0.06 (-0.12,-0.03)	-0.02 (-0.03,-0.01)
Nigeria	3.46 (2.13,5.58)	0.14 (-1.31,1.70)	3.60 (2.23,5.84)	5.85 (3.68,9.22)	0.20 (-2.09,2.55)	6.09 (3.82,9.39)	6.47 (4.00,10.51)	0.26 (-2.19,2.96)	6.75 (4.07,10.74)
Mexico	-0.42 (-0.56,-0.28)	0.01 (0.00,0.03)	-0.41 (-0.53,-0.28)	-0.45 (-0.60,-0.31)	0.02 (0.00,0.03)	-0.44 (-0.57,-0.31)	-0.46 (-0.61,-0.31)	0.02 (0.00,0.03)	-0.44 (-0.58,-0.31)
Germany	0.46 (0.29,0.64)	-0.25 (-0.34,-0.17)	0.21 (0.12,0.31)	0.46 (0.29,0.63)	-0.25 (-0.33,-0.17)	0.21 (0.11,0.30)	0.34 (0.21,0.46)	-0.18 (-0.24,-0.13)	0.15 (0.08,0.22)
Vietnam	0.44 (0.28,0.64)	-0.10 (-0.13,-0.07)	0.35 (0.21,0.50)	0.53 (0.33,0.76)	-0.12 (-0.16,-0.08)	0.42 (0.25,0.60)	0.53 (0.33,0.76)	-0.12 (-0.16,-0.08)	0.41 (0.25,0.60)
Philippines	-0.20 (-0.25,-0.16)	-0.01 (-0.01,-0.01)	-0.21 (-0.26,-0.17)	-0.24 (-0.29,-0.19)	-0.01 (-0.02,-0.01)	-0.25 (-0.31,-0.20)	-0.24 (-0.29,-0.19)	-0.01 (-0.02,-0.01)	-0.25 (-0.31,-0.20)
Egypt	-0.18 (-0.26,-0.11)	-0.03 (-0.04,-0.02)	-0.21 (-0.29,-0.14)	-0.19 (-0.28,-0.11)	-0.03 (-0.05,-0.02)	-0.23 (-0.31,-0.15)	-0.16 (-0.23,-0.09)	-0.03 (-0.04,-0.02)	-0.19 (-0.26,-0.12)
Iran	0.10 (0.07,0.13)	-0.04 (-0.08,0.00)	0.06 (0.00,0.12)	0.10 (0.08,0.13)	-0.04 (-0.08,0.00)	0.06 (0.00,0.13)	0.09 (0.06,0.11)	-0.03 (-0.07,0.00)	0.05 (0.00,0.11)
Turkey	-0.87 (-1.16,-0.65)	0.66 (0.50,0.89)	-0.21 (-0.27,-0.16)	-0.94 (-1.23,-0.72)	0.72 (0.55,0.94)	-0.22 (-0.29,-0.17)	-0.77 (-1.01,-0.58)	0.59 (0.44,0.77)	-0.18 (-0.24,-0.14)
Thailand	1.66 (0.82,3.39)	1.95 (0.96,4.01)	3.61 (1.78,7.36)	1.99 (0.97,4.16)	2.33 (1.14,4.86)	4.31 (2.12,9.05)	1.98 (0.97,4.11)	2.33 (1.13,4.82)	4.30 (2.10,8.94)
Ethiopia	1.37 (1.03,1.81)	1.36 (0.99,1.85)	2.73 (2.02,3.65)	2.30 (1.72,3.08)	2.29 (1.67,3.15)	4.60 (3.40,6.21)	2.57 (1.84,3.65)	2.56 (1.80,3.70)	5.13 (3.64,7.36)
United Kingdom	-0.27 (-0.35,-0.19)	0.11 (0.07,0.15)	-0.16 (-0.19,-0.13)	-0.26 (-0.34,-0.19)	0.11 (0.06,0.15)	-0.16 (-0.19,-0.13)	-0.19 (-0.25,-0.14)	0.08 (0.05,0.11)	-0.11 (-0.14,-0.09)
Italy	0.09 (0.08,0.11)	-0.03 (-0.03,-0.02)	0.07 (0.05,0.08)	0.09 (0.08,0.11)	-0.03 (-0.03,-0.02)	0.07 (0.05,0.08)	0.07 (0.06,0.08)	-0.02 (-0.02,-0.02)	0.05 (0.04,0.06)
France	0.60 (0.52,0.69)	0.39 (0.34,0.45)	1.00 (0.86,1.15)	0.59 (0.52,0.68)	0.39 (0.33,0.45)	0.98 (0.85,1.13)	0.44 (0.38,0.50)	0.28 (0.25,0.33)	0.72 (0.63,0.83)
Korea	0.03 (0.01,0.05)	0.01 (0.00,0.02)	0.04 (0.02,0.07)	0.04 (0.02,0.06)	0.01 (0.00,0.02)	0.05 (0.02,0.08)	0.04 (0.02,0.06)	0.01 (0.00,0.02)	0.05 (0.02,0.08)
Spain	0.53 (0.44,0.63)	0.27 (0.22,0.32)	0.80 (0.66,0.96)	0.52 (0.43,0.62)	0.27 (0.22,0.32)	0.78 (0.65,0.94)	0.38 (0.32,0.46)	0.19 (0.16,0.24)	0.58 (0.48,0.69)
DR Congo	0.24 (0.08,0.71)	0.08 (0.03,0.24)	0.32 (0.11,0.96)	0.40 (0.14,1.21)	0.14 (0.05,0.41)	0.53 (0.18,1.63)	0.45 (0.15,1.30)	0.15 (0.05,0.45)	0.60 (0.20,1.74)

*Data are mean absolute change in intakes (95% uncertainty interval) in 8 oz servings per week. Standardized serving size used for this analysis: 8 oz serving = 248 grams. Countries are ordered top to bottom from most to least populous based on 2018 adult (20+ years) population data. Source data are provided as Source Data file 7.

Supplementary Table 19. Absolute change in mean sugar-sweetened beverage intakes (8 oz servings/week) in adults (20+ years) by area of residence from 1990-2005, 2005-2018, and 1990-2018 by area of residence in the 25 most populous countries.

	Rural			Urban		
	1990-2005	2005-2018	1990-2018	1990-2005	2005-2018	1990-2018
Country	*mean (95% UI)*	*mean (95% UI)*	*mean (95% UI)*	*mean (95% UI)*	*mean (95% UI)*	*mean (95% UI)*
China	-0.06 (-0.07,-0.04)	0.00 (0.00,0.00)	-0.06 (-0.08,-0.05)	-0.06 (-0.07,-0.05)	0.00 (0.00,0.00)	-0.06 (-0.08,-0.05)
India	0.00 (0.00,0.00)	0.01 (0.00,0.01)	0.01 (0.00,0.01)	0.00 (-0.01,0.00)	0.04 (0.02,0.08)	0.03 (0.02,0.07)
United States	2.36 (2.21,2.52)	-2.89 (-3.08,-2.71)	-0.54 (-0.57,-0.51)	2.25 (2.08,2.44)	-2.76 (-2.99,-2.56)	-0.51 (-0.55,-0.47)
Indonesia	0.21 (0.16,0.28)	0.25 (0.18,0.33)	0.46 (0.34,0.61)	0.22 (0.16,0.29)	0.26 (0.19,0.35)	0.48 (0.35,0.64)
Brazil	-2.65 (-3.12,-2.26)	0.06 (0.05,0.08)	-2.59 (-3.04,-2.21)	-3.02 (-3.44,-2.64)	0.07 (0.06,0.09)	-2.95 (-3.36,-2.58)
Pakistan	-0.01 (-0.02,-0.01)	-0.16 (-0.26,-0.11)	-0.18 (-0.29,-0.12)	-0.07 (-0.11,-0.04)	-0.76 (-1.28,-0.45)	-0.83 (-1.39,-0.50)
Russia	1.44 (1.15,1.77)	-0.06 (-0.08,-0.04)	1.38 (1.10,1.71)	1.32 (1.07,1.61)	-0.05 (-0.07,-0.04)	1.26 (1.02,1.55)
Japan	-0.19 (-0.26,-0.14)	-0.09 (-0.13,-0.06)	-0.29 (-0.38,-0.20)	-0.20 (-0.27,-0.14)	-0.10 (-0.13,-0.06)	-0.30 (-0.40,-0.21)
Bangladesh	0.01 (0.01,0.02)	-0.02 (-0.03,-0.01)	-0.01 (-0.01,0.00)	0.05 (0.03,0.10)	-0.07 (-0.14,-0.04)	-0.02 (-0.04,-0.01)
Nigeria	3.22 (2.05,5.09)	0.14 (-1.16,1.48)	3.36 (2.14,5.28)	5.11 (3.21,8.10)	0.21 (-1.86,2.34)	5.29 (3.27,8.36)
Mexico	-0.40 (-0.53,-0.27)	0.01 (0.00,0.03)	-0.38 (-0.50,-0.27)	-0.45 (-0.60,-0.31)	0.02 (0.00,0.03)	-0.44 (-0.57,-0.31)
Germany	0.43 (0.27,0.60)	-0.23 (-0.31,-0.16)	0.20 (0.11,0.29)	0.41 (0.26,0.56)	-0.22 (-0.29,-0.16)	0.19 (0.10,0.27)
Vietnam	0.48 (0.30,0.69)	-0.11 (-0.15,-0.07)	0.38 (0.23,0.55)	0.50 (0.31,0.72)	-0.11 (-0.15,-0.08)	0.39 (0.24,0.57)
Philippines	-0.22 (-0.27,-0.18)	-0.01 (-0.01,-0.01)	-0.24 (-0.29,-0.19)	-0.23 (-0.29,-0.19)	-0.01 (-0.02,-0.01)	-0.25 (-0.30,-0.20)
Egypt	-0.19 (-0.28,-0.11)	-0.03 (-0.05,-0.02)	-0.22 (-0.30,-0.15)	-0.17 (-0.24,-0.10)	-0.03 (-0.04,-0.02)	-0.19 (-0.27,-0.13)
Iran	0.11 (0.08,0.14)	-0.04 (-0.08,0.00)	0.07 (0.00,0.13)	0.09 (0.07,0.12)	-0.04 (-0.07,0.00)	0.06 (0.00,0.11)
Turkey	-0.98 (-1.30,-0.74)	0.75 (0.56,0.99)	-0.23 (-0.30,-0.18)	-0.85 (-1.11,-0.65)	0.65 (0.49,0.85)	-0.20 (-0.26,-0.15)
Thailand	1.75 (0.86,3.60)	2.05 (1.00,4.22)	3.80 (1.88,7.82)	1.82 (0.90,3.75)	2.14 (1.05,4.42)	3.96 (1.96,8.18)
Ethiopia	1.50 (1.15,1.97)	1.49 (1.11,2.00)	2.99 (2.26,3.96)	2.39 (1.79,3.16)	2.38 (1.73,3.22)	4.77 (3.51,6.37)
United Kingdom	-0.26 (-0.33,-0.18)	0.11 (0.06,0.15)	-0.15 (-0.19,-0.12)	-0.25 (-0.32,-0.18)	0.10 (0.06,0.14)	-0.15 (-0.18,-0.12)
Italy	0.09 (0.07,0.11)	-0.02 (-0.03,-0.02)	0.06 (0.05,0.08)	0.08 (0.07,0.10)	-0.02 (-0.03,-0.02)	0.06 (0.05,0.07)
France	0.56 (0.49,0.65)	0.37 (0.32,0.43)	0.93 (0.81,1.08)	0.54 (0.47,0.61)	0.35 (0.30,0.40)	0.89 (0.77,1.02)
Korea	0.04 (0.02,0.06)	0.01 (0.00,0.02)	0.05 (0.02,0.08)	0.04 (0.02,0.06)	0.01 (0.00,0.02)	0.05 (0.02,0.08)
Spain	0.52 (0.42,0.62)	0.26 (0.22,0.32)	0.78 (0.64,0.93)	0.49 (0.41,0.59)	0.25 (0.21,0.30)	0.74 (0.62,0.89)
DR Congo	0.23 (0.08,0.67)	0.08 (0.03,0.23)	0.30 (0.10,0.90)	0.36 (0.12,1.07)	0.12 (0.04,0.37)	0.49 (0.17,1.44)

*Data are mean absolute change in intakes (95% uncertainty interval) in 8 oz servings per week. Standardized serving size used for this analysis: 8 oz serving = 248 grams. Countries are ordered top to bottom from most to least populous based on 2018 adult (20+ years) population data. Source data are provided as Source Data file 7.

Supplementary References

1 Vehtari, A., Gelman, A. & Gabry, J. Practical Bayesian model evaluation using leave-one-out cross-validation and WAIC. . *Stat Comput 27*, 1413-1432 (2017). https://doi.org/10.1007/s11222-016-9696-4.

2 Miller, V. *et al.* The Global Dietary Database 2015: data availability and gaps on 54 major foods, beverages and nutrients among 5.6 million children and adults from 1,220 surveys worldwide. *In Review* (2020).

3 Finucane, M. M. *et al.* National, regional, and global trends in body-mass index since 1980: systematic analysis of health examination surveys and epidemiological studies with 960 country-years and 9·1 million participants. *Lancet* **377**, 557-567 (2011). 10.1016/s0140-6736(10)62037-5

4 Flaxman, A. D., Vos, T. & Murray, C. J. *An Integrative Metaregression Framework for Descriptive Epidemiology*. (University of Washington Press, 2015).

5 Carpenter, B. *et al.* Stan: A Probabilistic Programming Language. *Journal of Statistical Software* **76**, 1 - 32 (2017). 10.18637/jss.v076.i01

6 Stan Development Team. Stan modeling language users guide and reference manual, version 2.27. (2021).

7 Stan Development Team. RStan: the R interface to Stan. (2019).

8 Homan, M. D. & Gelman, A. The No-U-turn sampler: adaptively setting path lengths in Hamiltonian Monte Carlo. *J. Mach. Learn. Res.* **15**, 1593–1623 (2014).

9 Radford, N. in *Handbook of Markov Chain Monte Carlo* (eds Steve Brooks, Andrew Gelman, Galin Jones, & Xiao-Li Meng) (Chapman and Hall/CRC, 2011).

10 Gelman, A. & Pardoe, L. Bayesian measures of explained variance and pooling in multilevel (hierarchical) models. *Technometrics* **48**, 241-251 (2006). 10.1198/004017005000000517

11 McElreath, R. Statistical Rethinking: A Bayesian Course with Examples in R and Stan. *Text Stat Sci*, 1-464 (2016). Book_Doi 10.1007/978-3-642-30574-0

12 Wagner, T., Diefenbach, D. R., Christensen, S. A. & Norton, A. S. Using Multilevel Models to Quantify Heterogeneity in Resource Selection. *J Wildlife Manage* **75**, 1788-1796 (2011). 10.1002/jwmg.212
