# Supplementary material for: Sugar-sweetened beverage intakes among adults between 1990 and 2018 in 185 countries
Source: Nat Commun. 2023 Oct 3;14:5957. doi: 10.1038/s41467-023-41269-8 (PMC10614169; doi:10.1038/s41467-023-41269-8)
Supplement: Supplementary file 3 — Description of Additional Supplementary Files [file 41467_2023_41269_MOESM3_ESM.pdf]

Description of Additional Supplementary Files

File name: Supplementary Data 1

Description: Characteristics of surveys included in the modeling for sugar-sweetened beverages in the Global Dietary Database. Includes survey characteristics for the 451 surveys with sugar-sweetened beverage intake data included in the GDD model. Countries are ordered alphabetically. FFQ: Food Frequency Questionnaires; DHS: Demographic and Health Survey.

File name: Supplementary Data 2

Description: Mean Intakes and Absolute Change by World Region and 185 Countries. Data are mean intakes (95% UI) or mean absolute change in intakes (95% UI) in 8 oz servings per day. All intakes are reported adjusted to 2,000 kcal/d for ages 20 to 74 years, and 1,700 kcal/d for ages 75+ years. Data are based on a Bayesian model that incorporated up to 451 individual-level dietary surveys, and additional survey-level and country-level covariates, to estimate dietary consumption levels. Total SSBs intake was defined as any beverage with added sugars having ≥50 kcal per 8 oz serving, including commercial or homemade beverages, soft drinks, energy drinks, fruit drinks, punch, lemonade, and aguas frescas. This definition excludes 100% fruit and vegetable juices and non-caloric artificially sweetened drinks. Standardized serving size used for this analysis: 8 oz serving = 248 grams. Source data are provided as Source Data files 1, 2, 6, and 7. In prior GDD reports, the region Central/ Eastern Europe and Central Asia was referred as Former Soviet Union, and Southeast and East Asia was referred as Asia. GDD, Global Dietary Database; oz, ounces; SSB, sugar-sweetened beverage; UI, uncertainty interval.

File name: Supplementary Data 3

Description: SSB tax by country, type, and year of implementation. Year of tax implementation is indicated as “1” under the corresponding year column. Years in which no country implemented a tax are not displayed. Countries that are included in the GDD are indicated as “1” in column GDD. The countries are ordered alphabetically. Reference: World Bank Group. 2023. Global SSB Tax Database. Washington, DC: World Bank Group. License: Creative Commons Attribution 4.0 International License (CC BY 4.0). GDD, Global Dietary Database; SSB, sugar-sweetened beverage; Struct, Structure.
