# Supplementary material for: Sugar-sweetened beverage intakes among adults between 1990 and 2018 in 185 countries
Source: Nat Commun. 2023 Oct 3;14:5957. doi: 10.1038/s41467-023-41269-8 (PMC10614169; doi:10.1038/s41467-023-41269-8)
Supplement: Supplementary file 4 — Supplementary Data 1 [file 41467_2023_41269_MOESM4_ESM.pdf]

Sugar-sweetened beverage intakes among adults between 1990 and 2018 in 185 countries

Supplementary Data 1 | Individual Survey Characteristics

Supplementary Data 1. Characteristics of surveys included in the modeling for sugar-sweetened beverages in the Global Dietary Database.*

Country[f]	Survey Name	Data Collection Round	Year Survey Began	Year Survey Ended	Residence	Sample Size	Youngest Age Baseline	Oldest Age Baseline	Sex	Diet Assessment Method‡	Representativeness
Algeria	Global Student Based Student Health Survey 2011	GDD 2018	2011	2011	Both urban and rural	4431	11	15	Both female and male	FFQ	Nationally representative
Antigua and Barbuda	Global Student Based Student Health Survey 2009	GDD 2018	2009	2009	Both urban and rural	1215	11	15	Both female and male	FFQ	Nationally representative
Argentina	Encuesta Alimentaria y Nutricional de la Ciudad de Buenos Aires	GDD 2018	2011	2011	Not reported	5370	0	85	Both female and male	Multiple 24-hour recall	Sub-nationally representative
Argentina	Encuesta Nacional de Nutrición y Salud - National Nutrition and Health Survey (ENNyS)	GDD 2010	2004	2005	Not reported	4429	20	45	Only female	Single 24-hour recall	Nationally representative
Argentina	Global Student Based Student Health Survey 2012	GDD 2018	2012	2012	Both urban and rural	27540	11	15	Both female and male	FFQ	Nationally representative
Argentina	Nutritional status of school-aged children of Buenos Aires	GDD 2018	2005	2005	Only urban	1571	6	11	Both female and male	FFQ	Locally representative
Argentina	Primer estudio sobre el estado nutricional y los hábitos alimentarios de la población adulta de Rosario	GDD 2018	2012	2013	Only urban	1195	15	65	Both female and male	Single 24-hour recall	Locally representative
Argentina	School Canteens Study	GDD 2018	2008	2008	Not reported	391	6	11	Both female and male	FFQ	Locally representative
Armenia	Health Behavior in School-Aged Children 2010	GDD 2018	2010	2010	Both urban and rural	2635	11	15	Both female and male	FFQ	Nationally representative
Australia	National Nutrition Survey	GDD 2010	1995	1996	Not reported	13858	2	65	Both female and male	Single 24-hour recall	Nationally representative
Austria	Austrian Community Health and Nutrition Survey	GDD 2010	1999	1999	Not reported	7098	0	0	Not reported	Household availability/budget survey	Nationally representative
Austria	Austrian Study on Nutritional Status	GDD 2010	2005	2006	Not reported	2123	19	19	Not reported	Single 24-hour recall	Nationally representative
Austria	Health Behavior in School-Aged Children 1990	GDD 2018	1989	1990	Both urban and rural	2886	6	15	Both female and male	FFQ	Nationally representative
Austria	Health Behavior in School-Aged Children 1994	GDD 2018	1993	1994	Both urban and rural	5129	6	15	Both female and male	FFQ	Nationally representative
Austria	Health Behavior in School-Aged Children 1998	GDD 2018	1998	1998	Both urban and rural	4281	6	15	Both female and male	FFQ	Nationally representative

Supplementary Data 1. Characteristics of surveys included in the modeling for sugar-sweetened beverages in the Global Dietary Database (continued).*

Country[f]	Survey Name	Data Collection Round	Year Survey Began	Year Survey Ended	Residence	Sample Size	Youngest Age Baseline	Oldest Age Baseline	Sex	Diet Assessment Method‡	Representativeness
Austria	Health Behavior in School-Aged Children 2002	GDD 2018	2001	2002	Both urban and rural	4335	6	15	Both female and male	FFQ	Nationally representative
Austria	Health Behavior in School-Aged Children 2006	GDD 2018	2006	2006	Both urban and rural	4767	11	15	Both female and male	FFQ	Nationally representative
Austria	Health Behavior in School-Aged Children 2010	GDD 2018	2010	2010	Both urban and rural	4972	11	15	Both female and male	FFQ	Nationally representative
Bahamas, The	Global Student Based Student Health Survey 2013	GDD 2018	2013	2013	Both urban and rural	1336	11	15	Both female and male	FFQ	Nationally representative
Bangladesh	Bangladesh Integrated Household Survey (BIHS)	GDD 2018	2011	2012	Only rural	22151	0	85	Both female and male	Single 24-hour recall	Nationally representative
Bangladesh	Demographic and Health Survey Bangladesh 1993	GDD 2018	1993	1994	Both urban and rural	3014	0	2	Both female and male	DHS questionnaire	Nationally representative
Bangladesh	Demographic and Health Survey Bangladesh 1996	GDD 2018	1996	1997	Both urban and rural	4308	0	2	Both female and male	DHS questionnaire	Nationally representative
Barbados	Global Student Based Student Health Survey 2011	GDD 2018	2011	2011	Both urban and rural	1601	11	15	Both female and male	FFQ	Nationally representative
Barbados	Identifying new genetic and obesity-related factors contributing to prostate and breast cancer risk in persons of African descent	GDD 2010	2004	2007	Not reported	277	31	85	Both female and male	FFQ	Nationally representative
Belgium	Belgian Community Health and Nutrition Survey	GDD 2010	1987	1988	Not reported	3235	0	0	Not reported	Household availability/budget survey	Nationally representative
Belgium	Belgian Community Health and Nutrition Survey	GDD 2010	1996	1997	Not reported	2041	0	0	Not reported	Household availability/budget survey	Nationally representative
Belgium	Belgian Community Health and Nutrition Survey	GDD 2010	1999	1999	Not reported	3745	0	0	Not reported	Household availability/budget survey	Nationally representative
Belgium	Belgian National Food Consumption Survey	GDD 2010	2004	2004	Not reported	2240	20	85	Both female and male	Multiple 24-hour recall	Nationally representative
Belgium	Flanders preschool dietary survey	GDD 2018	2002	2003	Both urban and rural	661	2	6	Both female and male	Multiple 24-hour recall	Sub-nationally representative

Supplementary Data 1. Characteristics of surveys included in the modeling for sugar-sweetened beverages in the Global Dietary Database (continued).*

Country[f]	Survey Name	Data Collection Round	Year Survey Began	Year Survey Ended	Residence	Sample Size	Youngest Age Baseline	Oldest Age Baseline	Sex	Diet Assessment Method[‡]	Representativeness
Belgium	Health Behavior in School-Aged Children 1986	GDD 2018	1985	1986	Both urban and rural	3565	6	15	Both female and male	FFQ	Nationally representative
Belgium	Health Behavior in School-Aged Children 1990	GDD 2018	1989	1990	Both urban and rural	2948	11	15	Both female and male	FFQ	Nationally representative
Belgium	Health Behavior in School-Aged Children 1994	GDD 2018	1993	1994	Both urban and rural	5033	6	15	Both female and male	FFQ	Sub-nationally representative
Belgium	Health Behavior in School-Aged Children 1994	GDD 2018	1993	1994	Both urban and rural	4465	11	15	Both female and male	FFQ	Sub-nationally representative
Belgium	Health Behavior in School-Aged Children 1998	GDD 2018	1998	1998	Both urban and rural	2494	11	15	Both female and male	FFQ	Sub-nationally representative
Belgium	Health Behavior in School-Aged Children 1998	GDD 2018	1998	1998	Both urban and rural	4015	11	15	Both female and male	FFQ	Sub-nationally representative
Belgium	Health Behavior in School-Aged Children 2002	GDD 2018	2001	2002	Both urban and rural	10584	11	15	Both female and male	FFQ	Nationally representative
Belgium	Health Behavior in School-Aged Children 2006	GDD 2018	2006	2006	Both urban and rural	8741	11	15	Both female and male	FFQ	Nationally representative
Belgium	Health Behavior in School-Aged Children 2010	GDD 2018	2010	2010	Both urban and rural	8144	11	15	Both female and male	FFQ	Nationally representative
Belize	Global Student Based Student Health Survey 2011	GDD 2018	2011	2011	Both urban and rural	2059	11	15	Both female and male	FFQ	Nationally representative
Benin	Global Student Based Student Health Survey 2009	GDD 2018	2009	2009	Both urban and rural	2662	11	15	Both female and male	FFQ	Nationally representative
Bolivia	Cardiovascular and metabolic syndrome risk assessment of Bolivian school children and adolescents: Relationships to obesity, diabetes, income, food intake and physical activity	GDD 2018	2005	2007	Both urban and rural	3445	11	15	Both female and male	FFQ	Nationally representative
Bolivia	Global Student Based Student Health Survey 2012	GDD 2018	2012	2012	Both urban and rural	3458	11	15	Both female and male	FFQ	Nationally representative
Brazil	Brazilian Study of Cardiovascular Risks in Adolescents (ERICA)	GDD 2018	2013	2014	Both urban and rural	64781	11	15	Both female and male	Multiple 24-hour recall	Nationally representative
Brazil	Consumer Expenditure Survey	GDD 2010	2008	2009	Not reported	34003	10	85	Both female and male	Multiple 24-hour recall	Nationally representative

Supplementary Data 1. Characteristics of surveys included in the modeling for sugar-sweetened beverages in the Global Dietary Database (continued).*

Country[ƒ]	Survey Name	Data Collection Round	Year Survey Began	Year Survey Ended	Residence	Sample Size	Youngest Age Baseline	Oldest Age Baseline	Sex	Diet Assessment Method[‡]	Representativeness
Brazil	Health Survey of São Paulo	GDD 2018	2008	2009	Only urban	1636	11	85	Both female and male	Multiple 24-hour recall	Sub-nationally representative
Brunei	Global Student Based Student Health Survey 2014	GDD 2018	2014	2014	Both urban and rural	2565	6	15	Both female and male	FFQ	Nationally representative
Bulgaria	Health Behavior in School-Aged Children 2006	GDD 2018	2006	2006	Both urban and rural	4753	11	15	Both female and male	FFQ	Nationally representative
Bulgaria	National Nutrition Survey	GDD 2010	2004	2004	Not reported	853	20	85	Both female and male	Single 24-hour recall	Nationally representative
Bulgaria	Nutrition of Children Survey (NUTRICHILD)	GDD 2018	2007	2007	Both urban and rural	1723	0	2	Both female and male	Multiple 24-hour recall	Nationally representative
Burkina Faso	Nutrition transition and double burden of malnutrition among adults in Ouagadougou, Burkina Faso (West Africa)	GDD 2018	2010	2010	Both urban and rural	310	25	55	Both female and male	Multiple 24-hour recall	Locally representative
Cambodia	Demographic and Health Survey Cambodia 2005	GDD 2018	2005	2006	Both urban and rural	6222	15	45	Only female	DHS questionnaire	Nationally representative
Cambodia	Global Student Based Student Health Survey 2013	GDD 2018	2013	2013	Both urban and rural	3787	6	15	Both female and male	FFQ	Nationally representative
Canada	Canadian Community Health Survey (CCHS)	GDD 2018	2015	2015	Not reported	20372	1	85	Both female and male	Multiple 24-hour recall	Nationally representative
Canada	Canadian Community Health and Nutrition Survey	GDD 2010	2004	2004	Not reported	34848	0	85	Both female and male	Multiple 24-hour recall	Nationally representative
Canada	Health Behavior in School-Aged Children 1990	GDD 2018	1989	1990	Both urban and rural	5541	11	15	Both female and male	FFQ	Nationally representative
Canada	Health Behavior in School-Aged Children 1994	GDD 2018	1993	1994	Both urban and rural	6746	6	15	Both female and male	FFQ	Nationally representative
Canada	Health Behavior in School-Aged Children 1998	GDD 2018	1998	1998	Both urban and rural	6557	11	15	Both female and male	FFQ	Nationally representative
Canada	Health Behavior in School-Aged Children 2002	GDD 2018	2001	2002	Both urban and rural	4351	11	15	Both female and male	FFQ	Nationally representative
Canada	Health Behavior in School-Aged Children 2006	GDD 2018	2006	2006	Both urban and rural	5771	11	15	Both female and male	FFQ	Nationally representative
Canada	Health Behavior in School-Aged Children 2010	GDD 2018	2010	2010	Both urban and rural	15613	11	15	Both female and male	FFQ	Nationally representative

Supplementary Data 1. Characteristics of surveys included in the modeling for sugar-sweetened beverages in the Global Dietary Database (continued).*

Country[ʃ]	Survey Name	Data Collection Round	Year Survey Began	Year Survey Ended	Residence	Sample Size	Youngest Age Baseline	Oldest Age Baseline	Sex	Diet Assessment Method[‡]	Representativeness
Chile	Estado nutricional, consumo de alimentos y actividad física en escolares mujeres de diferente nivel socioeconómico de Santiago de Chile	GDD 2018	2003	2004	Not reported	202	6	11	Only female	FFQ	Sub-nationally representative
Chile	Estado nutricional, consumo de alimentos y actividad física en escolares mujeres de diferente nivel socioeconómico de Santiago de Chile	GDD 2018	2003	2004	Both urban and rural	356	6	11	Only female	FFQ	Sub-nationally representative
Chile	Global Student Based Student Health Survey 2013	GDD 2018	2013	2013	Both urban and rural	2023	6	15	Both female and male	FFQ	Nationally representative
China	2002 China National Nutrition and Health Survey	GDD 2010	2002	2002	Not reported	65249	1	85	Both female and male	Multiple 24-hour recall	Nationally representative
Colombia	Database from National survey of nutritional situation in Colombia	GDD 2010	2005	2005	Not reported	11409	20	55	Both female and male	Multiple 24-hour recall	Nationally representative
Congo, Dem. Rep.	Women First Preconception Maternal Nutrition RCT	GDD 2018	2014	2016	Only rural	214	15	25	Only female	Multiple 24-hour recall	Locally representative
Costa Rica	Global Student Based Student Health Survey 2009	GDD 2018	2009	2009	Both urban and rural	2655	11	15	Both female and male	FFQ	Nationally representative
Croatia	Croatian Community Health and Nutrition Survey	GDD 2010	1999	1999	Not reported	2937	0	0	Not reported	Household availability/budget survey	Nationally representative
Croatia	Croatian Community Health and Nutrition Survey	GDD 2010	2004	2004	Not reported	2847	0	0	Not reported	Household availability/budget survey	Nationally representative
Croatia	Health Behavior in School-Aged Children 2002	GDD 2018	2001	2002	Both urban and rural	4346	6	15	Both female and male	FFQ	Nationally representative
Croatia	Health Behavior in School-Aged Children 2006	GDD 2018	2006	2006	Both urban and rural	4927	11	15	Both female and male	FFQ	Nationally representative
Croatia	Health Behavior in School-Aged Children 2010	GDD 2018	2010	2010	Both urban and rural	6218	11	15	Both female and male	FFQ	Nationally representative
Cyprus	Cypriot Community Health and Nutrition Survey	GDD 2010	1996	1997	Not reported	3308	0	0	Not reported	Household availability/budget survey	Nationally representative

Supplementary Data 1. Characteristics of surveys included in the modeling for sugar-sweetened beverages in the Global Dietary Database (continued).*

Country[ʃ]	Survey Name	Data Collection Round	Year Survey Began	Year Survey Ended	Residence	Sample Size	Youngest Age Baseline	Oldest Age Baseline	Sex	Diet Assessment Method[‡]	Representativeness
Cyprus	Cypriot Community Health and Nutrition Survey	GDD 2010	2003	2003	Not reported	2990	0	0	Not reported	Household availability/budget survey	Nationally representative
Czech Republic	Health Behavior in School-Aged Children 1994	GDD 2018	1993	1994	Both urban and rural	3579	11	15	Both female and male	FFQ	Nationally representative
Czech Republic	Health Behavior in School-Aged Children 1998	GDD 2018	1998	1998	Both urban and rural	3646	6	15	Both female and male	FFQ	Nationally representative
Czech Republic	Health Behavior in School-Aged Children 2002	GDD 2018	2001	2002	Both urban and rural	5000	6	15	Both female and male	FFQ	Nationally representative
Czech Republic	Health Behavior in School-Aged Children 2006	GDD 2018	2006	2006	Both urban and rural	4747	11	15	Both female and male	FFQ	Nationally representative
Czech Republic	Health Behavior in School-Aged Children 2010	GDD 2018	2010	2010	Both urban and rural	4386	11	15	Both female and male	FFQ	Nationally representative
Czech Republic	Individual food consumption study	GDD 2010	2003	2004	Not reported	1751	16	16	Not reported	Multiple 24-hour recall	Nationally representative
Denmark	Health Behavior in School-Aged Children 1994	GDD 2018	1993	1994	Both urban and rural	3789	6	15	Both female and male	FFQ	Nationally representative
Denmark	Health Behavior in School-Aged Children 1998	GDD 2018	1998	1998	Both urban and rural	4919	6	15	Both female and male	FFQ	Nationally representative
Denmark	Health Behavior in School-Aged Children 2002	GDD 2018	2001	2002	Both urban and rural	4549	6	15	Both female and male	FFQ	Nationally representative
Denmark	Health Behavior in School-Aged Children 2006	GDD 2018	2006	2006	Both urban and rural	5659	11	15	Both female and male	FFQ	Nationally representative
Denmark	Health Behavior in School-Aged Children 2010	GDD 2018	2010	2010	Both urban and rural	4007	11	15	Both female and male	FFQ	Nationally representative
Dominica	Global Student Based Student Health Survey 2009	GDD 2018	2009	2009	Both urban and rural	1616	11	15	Both female and male	FFQ	Nationally representative
Egypt, Arab Rep.	Demographic and Health Survey Egypt 1995	GDD 2018	1995	1995	Both urban and rural	7660	0	2	Both female and male	DHS questionnaire	Nationally representative
Egypt, Arab Rep.	Demographic and Health Survey Egypt 2000	GDD 2018	2000	2000	Both urban and rural	10912	0	2	Both female and male	DHS questionnaire	Nationally representative
Egypt, Arab Rep.	Demographic and Health Survey Egypt 2005	GDD 2018	2005	2005	Both urban and rural	19159	0	45	Both female and male	DHS questionnaire	Nationally representative

Supplementary Data 1. Characteristics of surveys included in the modeling for sugar-sweetened beverages in the Global Dietary Database (continued).*

Country[f]	Survey Name	Data Collection Round	Year Survey Began	Year Survey Ended	Residence	Sample Size	Youngest Age Baseline	Oldest Age Baseline	Sex	Diet Assessment Method[‡]	Representativeness
Egypt, Arab Rep.	Global Student Based Student Health Survey 2011	GDD 2018	2011	2011	Both urban and rural	2451	11	15	Both female and male	FFQ	Nationally representative
El Salvador	Global Student Based Student Health Survey 2013	GDD 2018	2013	2013	Both urban and rural	1858	11	15	Both female and male	FFQ	Nationally representative
Estonia	Health Behavior in School-Aged Children 1994	GDD 2018	1993	1994	Both urban and rural	3490	6	15	Both female and male	FFQ	Nationally representative
Estonia	Health Behavior in School-Aged Children 1998	GDD 2018	1998	1998	Both urban and rural	1896	11	15	Both female and male	FFQ	Nationally representative
Estonia	Health Behavior in School-Aged Children 2002	GDD 2018	2001	2002	Both urban and rural	3976	6	15	Both female and male	FFQ	Nationally representative
Estonia	Health Behavior in School-Aged Children 2006	GDD 2018	2006	2006	Both urban and rural	4450	11	15	Both female and male	FFQ	Nationally representative
Estonia	Health Behavior in School-Aged Children 2010	GDD 2018	2010	2010	Both urban and rural	4201	11	15	Both female and male	FFQ	Nationally representative
Estonia	National Dietary Survey (RTU)	GDD 2018	2013	2015	Not reported	3039	11	65	Both female and male	Multiple 24-hour recall	Nationally representative
Ethiopia (excludes Eritrea)	Demographic and Health Study 2005	GDD 2018	2005	2005	Both urban and rural	8153	0	2	Both female and male	DHS questionnaire	Nationally representative
Ethiopia (excludes Eritrea)	Dietary Practices, Maternal Nutritional Status and Child Stunting: Comparative and Intervention Studies in Pulse and Non-pulse Growing Rural Communities in Ethiopia	GDD 2018	2013	2013	Only rural	427	0	35	Both female and male	Single food record	Sub-nationally representative
Ethiopia (excludes Eritrea)	Nutritional status and dietary intake of urban residents in Gondar, Northwest Ethiopia	GDD 2018	2004	2007	Only urban	34	25	45	Both female and male	Single 24-hour recall	Sub-nationally representative
Finland	FINDIET 1992	GDD 2010	1992	1992	Not reported	1861	25	55	Both female and male	Multiple 24-hour recall	Nationally representative
Finland	FINDIET 2007	GDD 2010	2007	2007	Not reported	2039	25	65	Both female and male	Multiple 24-hour recall	Nationally representative
Finland	Finn Community Health and Nutrition Survey	GDD 2010	1985	1985	Not reported	8200	0	0	Not reported	Household availability/budget survey	Nationally representative

Supplementary Data 1. Characteristics of surveys included in the modeling for sugar-sweetened beverages in the Global Dietary Database (continued).*

Country[f]	Survey Name	Data Collection Round	Year Survey Began	Year Survey Ended	Residence	Sample Size	Youngest Age Baseline	Oldest Age Baseline	Sex	Diet Assessment Method[‡]	Representativeness
Finland	Finn Community Health and Nutrition Survey	GDD 2010	1990	1990	Not reported	8258	0	0	Not reported	Household availability/budget survey	Nationally representative
Finland	Finn Community Health and Nutrition Survey	GDD 2010	1998	1998	Not reported	4359	0	0	Not reported	Household availability/budget survey	Nationally representative
Finland	Health Behavior in School-Aged Children 1986	GDD 2018	1985	1986	Both urban and rural	3178	11	15	Both female and male	FFQ	Nationally representative
Finland	Health Behavior in School-Aged Children 1990	GDD 2018	1989	1990	Both urban and rural	2920	6	15	Both female and male	FFQ	Nationally representative
Finland	Health Behavior in School-Aged Children 1994	GDD 2018	1993	1994	Both urban and rural	4033	11	15	Both female and male	FFQ	Nationally representative
Finland	Health Behavior in School-Aged Children 1998	GDD 2018	1998	1998	Both urban and rural	4809	11	15	Both female and male	FFQ	Nationally representative
Finland	Health Behavior in School-Aged Children 2002	GDD 2018	2001	2002	Both urban and rural	5340	6	15	Both female and male	FFQ	Nationally representative
Finland	Health Behavior in School-Aged Children 2006	GDD 2018	2006	2006	Both urban and rural	5180	11	15	Both female and male	FFQ	Nationally representative
Finland	Health Behavior in School-Aged Children 2010	GDD 2018	2010	2010	Both urban and rural	6604	11	15	Both female and male	FFQ	Nationally representative
Finland	Increased Health and Wellbeing in Preschools (DAGIS) cross-sectional survey	GDD 2018	2015	2016	Both urban and rural	815	2	6	Both female and male	Multiple food record	Sub-nationally representative
France	Enquete Individuelle et Nationale sur les Consommations Alimentaires	GDD 2010	1999	1999	Not reported	1195	16	16	Not reported	Multiple 24-hour recall	Nationally representative
France	Etude nationale nutrition santè (ENNS); National Nutrition and Health survey	GDD 2018	2006	2007	Both urban and rural	4374	3	65	Both female and male	Multiple 24-hour recall	Nationally representative
France	Health Behavior in School-Aged Children 1994	GDD 2018	1993	1994	Both urban and rural	3982	6	15	Both female and male	FFQ	Nationally representative
France	Health Behavior in School-Aged Children 1998	GDD 2018	1998	1998	Both urban and rural	4128	11	15	Both female and male	FFQ	Sub-nationally representative
France	Health Behavior in School-Aged Children 2002	GDD 2018	2001	2002	Both urban and rural	8155	6	15	Both female and male	FFQ	Nationally representative

Supplementary Data 1. Characteristics of surveys included in the modeling for sugar-sweetened beverages in the Global Dietary Database (continued).*

Country[∫]	Survey Name	Data Collection Round	Year Survey Began	Year Survey Ended	Residence	Sample Size	Youngest Age Baseline	Oldest Age Baseline	Sex	Diet Assessment Method[‡]	Representativeness
France	Health Behavior in School-Aged Children 2006	GDD 2018	2006	2006	Both urban and rural	7066	11	15	Both female and male	FFQ	Nationally representative
France	Health Behavior in School-Aged Children 2010	GDD 2018	2010	2010	Both urban and rural	6045	11	15	Both female and male	FFQ	Nationally representative
Germany	DOrtmund Nutritional and Anthropometric Longitudinally Designed Study (DONALD)	GDD 2018	2008	2008	Only urban	921	1	6	Both female and male	Multiple food record	Locally representative
Germany	EsKiMo	GDD 2018	2006	2006	Both urban and rural	1257	11	15	Both female and male	FFQ	Nationally representative
Germany	German Community Health and Nutrition Survey	GDD 2010	1988	1988	Not reported	17855	0	0	Not reported	Household availability/budget survey	Nationally representative
Germany	German Community Health and Nutrition Survey	GDD 2010	1993	1993	Not reported	15825	0	0	Not reported	Household availability/budget survey	Nationally representative
Germany	German Community Health and Nutrition Survey	GDD 2010	1998	1998	Not reported	12680	0	0	Not reported	Household availability/budget survey	Nationally representative
Germany	German Nutrition Survey 1998	GDD 2018	1997	1999	Both urban and rural	4008	15	75	Both female and male	FFQ	Nationally representative
Germany	German Nutrition Survey 1998	GDD 2010	1997	1999	Not reported	3861	20	75	Both female and male	FFQ	Nationally representative
Germany	Health Behavior in School-Aged Children 1994	GDD 2018	1993	1994	Both urban and rural	3248	6	15	Both female and male	FFQ	Nationally representative
Germany	Health Behavior in School-Aged Children 1998	GDD 2018	1998	1998	Both urban and rural	4767	6	15	Both female and male	FFQ	Sub-nationally representative
Germany	Health Behavior in School-Aged Children 2002	GDD 2018	2001	2002	Both urban and rural	5605	6	15	Both female and male	FFQ	Nationally representative
Germany	Health Behavior in School-Aged Children 2006	GDD 2018	2006	2006	Both urban and rural	7139	11	15	Both female and male	FFQ	Nationally representative
Germany	Health Behavior in School-Aged Children 2010	GDD 2018	2010	2010	Both urban and rural	4932	11	15	Both female and male	FFQ	Nationally representative
Ghana	Global School-Based Student Health Survey Junior High 2012	GDD 2018	2012	2012	Both urban and rural	1606	11	15	Both female and male	FFQ	Nationally representative

Supplementary Data 1. Characteristics of surveys included in the modeling for sugar-sweetened beverages in the Global Dietary Database (continued).*

Country[ƒ]	Survey Name	Data Collection Round	Year Survey Began	Year Survey Ended	Residence	Sample Size	Youngest Age Baseline	Oldest Age Baseline	Sex	Diet Assessment Method[‡]	Representativeness
Ghana	Global School-Based Student Health Survey Senior High 2012	GDD 2018	2012	2012	Both urban and rural	1954	11	15	Both female and male	FFQ	Nationally representative
Ghana	Modeling the Epidemiologic Transition Study (METS) - Ghana	GDD 2018	2010	2012	Only rural	483	25	45	Both female and male	Multiple 24-hour recall	Locally representative
Greece	ATTICA Study	GDD 2010	2001	2002	Not reported	1017	18	65	Both female and male	FFQ	Sub-nationally representative
Greece	Greek Community Health Survey and Nutrition Nutrition	GDD 2010	1981	1981	Not reported	6034	0	0	Not reported	Household availability/budget survey	Nationally representative
Greece	Greek Community Health Survey and Nutrition Nutrition	GDD 2010	1987	1987	Not reported	6489	0	0	Not reported	Household availability/budget survey	Nationally representative
Greece	Greek Community Health Survey and Nutrition Nutrition	GDD 2010	1998	1998	Not reported	6258	0	0	Not reported	Household availability/budget survey	Nationally representative
Greece	Greek Community Health Survey and Nutrition Nutrition	GDD 2010	2004	2004	Not reported	6555	0	0	Not reported	Household availability/budget survey	Nationally representative
Greece	Greek component of the European Prospective Investigation into Cancer and Nutrition (EPIC)	GDD 2010	1994	1999	Not reported	28024	25	75	Both female and male	FFQ	Locally representative
Greece	Health Behavior in School-Aged Children 1998	GDD 2018	1998	1998	Both urban and rural	4205	6	15	Both female and male	FFQ	Nationally representative
Greece	Health Behavior in School-Aged Children 2002	GDD 2018	2001	2002	Both urban and rural	3766	11	15	Both female and male	FFQ	Nationally representative
Greece	Health Behavior in School-Aged Children 2006	GDD 2018	2006	2006	Both urban and rural	3663	11	15	Both female and male	FFQ	Nationally representative
Greece	Health Behavior in School-Aged Children 2010	GDD 2018	2010	2010	Both urban and rural	4863	11	15	Both female and male	FFQ	Nationally representative
Guatemala	Demographic and Health Survey Guatemala 1995	GDD 2018	1995	1995	Both urban and rural	5786	0	2	Both female and male	DHS questionnaire	Nationally representative
Guatemala	Demographic and Health Survey Guatemala 1998	GDD 2018	1998	1999	Both urban and rural	2899	0	2	Both female and male	DHS questionnaire	Nationally representative

Supplementary Data 1. Characteristics of surveys included in the modeling for sugar-sweetened beverages in the Global Dietary Database (continued).*

Country[ʃ]	Survey Name	Data Collection Round	Year Survey Began	Year Survey Ended	Residence	Sample Size	Youngest Age Baseline	Oldest Age Baseline	Sex	Diet Assessment Method[‡]	Representativeness
Guatemala	Demographic and Health Survey Guatemala 2014	GDD 2018	2014	2015	Both urban and rural	37889	0	45	Both female and male	DHS questionnaire	Nationally representative
Guatemala	Global Student Based Student Health Survey 2009	GDD 2018	2009	2009	Both urban and rural	5438	11	15	Both female and male	FFQ	Nationally representative
Guyana	Global Student Based Student Health Survey 2010	GDD 2018	2010	2010	Both urban and rural	2338	11	15	Both female and male	FFQ	Nationally representative
Haiti	Demographic and Health Survey Haiti 1995	GDD 2018	1995	1995	Both urban and rural	995	0	2	Both female and male	DHS questionnaire	Nationally representative
Honduras	Demographic and Health Survey Honduras 2005	GDD 2018	2005	2006	Both urban and rural	5557	15	45	Only female	DHS questionnaire	Nationally representative
Honduras	Global Student Based Student Health Survey 2012	GDD 2018	2012	2012	Both urban and rural	1729	11	15	Both female and male	FFQ	Nationally representative
Hungary	Health Behavior in School-Aged Children 1986	GDD 2018	1985	1986	Both urban and rural	4448	11	15	Both female and male	FFQ	Nationally representative
Hungary	Health Behavior in School-Aged Children 1990	GDD 2018	1989	1990	Both urban and rural	5460	11	15	Both female and male	FFQ	Nationally representative
Hungary	Health Behavior in School-Aged Children 1998	GDD 2018	1998	1998	Both urban and rural	3599	11	15	Both female and male	FFQ	Nationally representative
Hungary	Health Behavior in School-Aged Children 2002	GDD 2018	2001	2002	Both urban and rural	4044	6	15	Both female and male	FFQ	Nationally representative
Hungary	Health Behavior in School-Aged Children 2006	GDD 2018	2006	2006	Both urban and rural	3467	11	15	Both female and male	FFQ	Nationally representative
Hungary	Health Behavior in School-Aged Children 2010	GDD 2018	2010	2010	Both urban and rural	4762	11	15	Both female and male	FFQ	Nationally representative
Hungary	Hungarian Community Health and Nutrition Survey	GDD 2010	1991	1991	Not reported	11813	0	0	Not reported	Household availability/budget survey	Nationally representative
Hungary	The 3rd National Hungarian Survey	GDD 2010	2003	2004	Not reported	927	18	18	Not reported	Multiple 24-hour recall	Nationally representative
Iceland	Diet of Icelandic 9- and 15-year-old children and adolescents	GDD 2010	2003	2004	Not reported	345	9	15	Both female and male	Multiple 24-hour recall	Nationally representative
Iceland	Diet of Icelandic schoolchildren	GDD 2010	1992	1993	Both urban and rural	780	9	15	Both female and male	Single 24-hour recall	Nationally representative

Supplementary Data 1. Characteristics of surveys included in the modeling for sugar-sweetened beverages in the Global Dietary Database (continued).*

Country[f]	Survey Name	Data Collection Round	Year Survey Began	Year Survey Ended	Residence	Sample Size	Youngest Age Baseline	Oldest Age Baseline	Sex	Diet Assessment Method[‡]	Representativeness
Iceland	Dietary Survey of the Icelanders	GDD 2010	1990	1990	Not reported	1095	20	75	Both female and male	Single 24-hour recall	Nationally representative
Iceland	Health Behavior in School-Aged Children 2006	GDD 2018	2006	2006	Not reported	9374	11	15	Both female and male	FFQ	Nationally representative
Iceland	Health Behavior in School-Aged Children 2010	GDD 2018	2010	2010	Both urban and rural	10971	11	15	Both female and male	FFQ	Nationally representative
Iceland	Research on infant nutrition in Iceland	GDD 2010	1995	1997	Both urban and rural	111	1	1	Both female and male	Multiple 24-hour recall	Nationally representative
Iceland	The Diet of Icelanders, Dietary Survey of The Icelandic Nutrition Council 2002	GDD 2010	2002	2002	Not reported	1118	20	75	Both female and male	Single 24-hour recall	Nationally representative
Iceland	The diet of Icelandic infants	GDD 2010	2005	2007	Not reported	365	1	1	Both female and male	Multiple 24-hour recall	Nationally representative
India	Diet and Nutritional Status of Rural Population, Prevalence of Hypertension and Diabetes among Adults, and Infant & Young child feeding practices	GDD 2018	2009	2012	Not reported	39719	0	85	Both female and male	Single 24-hour recall	Nationally representative
India	Prevalence of Coronary Heart Disease (CHD) and Its Risk Factors in Residents of Urban and Rural Areas of National Capital Region - A Repeat Survey	GDD 2018	2010	2012	Only rural	1398	25	85	Both female and male	FFQ	Locally representative
India	Urban School Children Survey	GDD 2018	2012	2012	Only rural	1384	6	15	Both female and male	FFQ	Locally representative
Indonesia	Cardiovascular disease risk of adult in west Sumatera, Indonesia	GDD 2018	2006	2006	Only urban	794	15	85	Both female and male	Single 24-hour recall	Sub-nationally representative
Iran, Islamic Rep.	Comprehensive National Household Food Consumption Survey	GDD 2010	1990	1995	Not reported	69	0	0	Not reported	Multiple 24-hour recall	Nationally representative
Iran, Islamic Rep.	Non communicable disease surveillance in Islamic Republic of Iran	GDD 2010	2005	2005	Not reported	72714	20	55	Both female and male	Single 24-hour recall	Nationally representative
Iran, Islamic Rep.	Tehran Lipid and Glucose Study	GDD 2018	2005	2008	Not reported	2771	20	75	Both female and male	FFQ	Locally representative
Iran, Islamic Rep.	Tehran Lipid and Glucose Study	GDD 2018	2009	2011	Only urban	6107	20	85	Both female and male	FFQ	Locally representative
Iran, Islamic Rep.	Tehran Lipid and Glucose Study	GDD 2018	2012	2015	Only urban	5689	20	85	Both female and male	FFQ	Locally representative

Supplementary Data 1. Characteristics of surveys included in the modeling for sugar-sweetened beverages in the Global Dietary Database (continued).*

Country[f]	Survey Name	Data Collection Round	Year Survey Began	Year Survey Ended	Residence	Sample Size	Youngest Age Baseline	Oldest Age Baseline	Sex	Diet Assessment Method[‡]	Representativeness
Iran, Islamic Rep.	The CASPIAN IV study	GDD 2018	2012	2012	Both urban and rural	9689	6	15	Both female and male	FFQ	Nationally representative
Iran, Islamic Rep.	Yazd Health Study	GDD 2018	2014	2015	Only urban	9780	20	60	Both female and male	FFQ	Sub-nationally representative
Iraq	Global Student Based Student Health Survey 2012	GDD 2018	2012	2012	Both urban and rural	1976	11	15	Both female and male	FFQ	Nationally representative
Ireland	Health Behavior in School-Aged Children 1998	GDD 2018	1997	1998	Both urban and rural	4377	11	15	Both female and male	FFQ	Nationally representative
Ireland	Health Behavior in School-Aged Children 2002	GDD 2018	2001	2002	Both urban and rural	2869	6	15	Both female and male	FFQ	Nationally representative
Ireland	Health Behavior in School-Aged Children 2006	GDD 2018	2006	2006	Both urban and rural	4787	11	15	Both female and male	FFQ	Nationally representative
Ireland	Health Behavior in School-Aged Children 2010	GDD 2018	2010	2010	Both urban and rural	4641	11	15	Both female and male	FFQ	Nationally representative
Ireland	Irish Community Health and Nutrition Survey	GDD 2010	1987	1987	Not reported	7705	0	0	Not reported	Household availability/budget survey	Nationally representative
Ireland	Irish Community Health and Nutrition Survey	GDD 2010	1994	1994	Not reported	7877	0	0	Not reported	Household availability/budget survey	Nationally representative
Ireland	Irish Community Health and Nutrition Survey	GDD 2010	1999	1999	Not reported	7644	0	0	Not reported	Household availability/budget survey	Nationally representative
Ireland	North South Ireland Food Consumption Survey (NSIFCS)	GDD 2010	1997	1999	Not reported	1373	18	18	Not reported	Multiple 24-hour recall	Nationally representative
Israel	Health Behavior in School-Aged Children 1994	GDD 2018	1993	1994	Both urban and rural	4202	11	15	Both female and male	FFQ	Nationally representative
Israel	Health Behavior in School-Aged Children 1998	GDD 2018	1998	1998	Both urban and rural	4726	11	15	Both female and male	FFQ	Nationally representative
Israel	Health Behavior in School-Aged Children 2002	GDD 2018	2001	2002	Both urban and rural	5265	11	15	Both female and male	FFQ	Nationally representative
Israel	Health Behavior in School-Aged Children 2006	GDD 2018	2006	2006	Both urban and rural	5228	11	15	Both female and male	FFQ	Nationally representative

Supplementary Data 1. Characteristics of surveys included in the modeling for sugar-sweetened beverages in the Global Dietary Database (continued).[*]

Country[ƒ]	Survey Name	Data Collection Round	Year Survey Began	Year Survey Ended	Residence	Sample Size	Youngest Age Baseline	Oldest Age Baseline	Sex	Diet Assessment Method[‡]	Representativeness
Israel	Health Behavior in School-Aged Children 2010	GDD 2018	2010	2010	Both urban and rural	4098	11	15	Both female and male	FFQ	Nationally representative
Israel	Mabat Elderly National Health and Nutrition Survey	GDD 2018	2005	2006	Only urban	326	65	85	Both female and male	Single 24-hour recall	Nationally representative
Israel	Mabat First National Health and Nutrition Survey	GDD 2018	1999	2001	Both urban and rural	1389	25	55	Both female and male	Single 24-hour recall	Nationally representative
Israel	Mabat Youth National Health and Nutrition Survey	GDD 2018	2003	2004	Both urban and rural	395	11	15	Both female and male	Single 24-hour recall	Nationally representative
Italy	Health Behavior in School-Aged Children 2002	GDD 2018	2001	2002	Both urban and rural	4322	6	15	Both female and male	FFQ	Nationally representative
Italy	Health Behavior in School-Aged Children 2006	GDD 2018	2005	2006	Both urban and rural	3899	11	15	Both female and male	FFQ	Nationally representative
Italy	Health Behavior in School-Aged Children 2010	GDD 2018	2009	2010	Both urban and rural	4801	11	15	Both female and male	FFQ	Nationally representative
Italy	INN 1980-84	GDD 2010	1980	1984	Not reported	36000	1	1	Not reported	Multiple 24-hour recall	Nationally representative
Italy	INN-CA 1994-96	GDD 2010	1994	1996	Not reported	1617	20	85	Both female and male	Multiple 24-hour recall	Nationally representative
Italy	INRAN-SCAI 2005-06	GDD 2010	2005	2006	Not reported	2777	20	85	Both female and male	Multiple 24-hour recall	Nationally representative
Italy	Italian Community Health and Nutrition Survey	GDD 2010	1990	1990	Not reported	33172	0	0	Not reported	Household availability/budget survey	Nationally representative
Italy	Italian Community Health and Nutrition Survey	GDD 2010	1993	1993	Not reported	34273	0	0	Not reported	Household availability/budget survey	Nationally representative
Italy	Italian Community Health and Nutrition Survey	GDD 2010	1996	1996	Not reported	22740	0	0	Not reported	Household availability/budget survey	Nationally representative
Italy	Mediterranean healthy Eating, Aging and Lifestyles (MEAL) study	GDD 2018	2014	2016	Not reported	1957	15	85	Both female and male	FFQ	Locally representative
Italy	Moli-Sani Study	GDD 2018	2005	2010	Both urban and rural	23348	35	85	Both female and male	FFQ	Sub-nationally representative

Supplementary Data 1. Characteristics of surveys included in the modeling for sugar-sweetened beverages in the Global Dietary Database (continued).*

Country^f	Survey Name	Data Collection Round	Year Survey Began	Year Survey Ended	Residence	Sample Size	Youngest Age Baseline	Oldest Age Baseline	Sex	Diet Assessment Method‡	Representativeness
Jamaica	Global Student Based Student Health Survey 2010	GDD 2018	2010	2010	Both urban and rural	1576	11	15	Both female and male	FFQ	Nationally representative
Jamaica	Social and dietary determinants of body max index of adult Jamaicans	GDD 2010	1993	1995	Not reported	922	25	65	Both female and male	FFQ	Nationally representative
Japan	Japan Public Health Center-based prospective Study (JPHC Study) Cohort II	GDD 2018	1993	2000	Not reported	55720	45	65	Both female and male	FFQ	Sub-nationally representative
Japan	Japan Public Health Center-based prospective Study (JPHC) Cohort I	GDD 2018	1990	1995	Not reported	42115	45	55	Both female and male	FFQ	Locally representative
Japan	Japan Public Health Center-based prospective Study (JPHC) Cohort I	GDD 2018	1990	1995	Not reported	41536	45	65	Both female and male	FFQ	Locally representative
Japan	Japan Public Health Center-based prospective Study (JPHC) Cohort II	GDD 2018	1993	2000	Not reported	50766	45	75	Both female and male	FFQ	Sub-nationally representative
Japan	National Nutrition Survey (in Japan)	GDD 2010	1995	1995	Not reported	10766	20	85	Both female and male	Single 24-hour recall	Nationally representative
Japan	National Nutrition Survey (in Japan)	GDD 2010	1998	1998	Not reported	10917	20	85	Both female and male	Single 24-hour recall	Nationally representative
Japan	The Japan Public Health Center-based Prospective Study (JPHC Study)	GDD 2010	1995	1998	Not reported	97768	45	65	Both female and male	FFQ	Locally representative
Japan	The Japan Public Health Center-based Prospective Study (JPHC Study)	GDD 2010	2000	2003	Not reported	92302	50	75	Both female and male	FFQ	Locally representative
Jordan	Demographic and Health Survey Jordan 2002	GDD 2018	2002	2002	Both urban and rural	1456	0	2	Both female and male	DHS questionnaire	Nationally representative
Jordan	Global Student Based Student Health Survey 2010	GDD 2018	2010	2010	Both urban and rural	1475	11	15	Both female and male	FFQ	Nationally representative
Kenya	Kenya National Micronutrient Survey	GDD 2018	2011	2011	Both urban and rural	826	0	45	Both female and male	Multiple 24-hour recall	Nationally representative
Kiribati	Global Student Based Student Health Survey 2011	GDD 2018	2011	2011	Both urban and rural	1551	11	15	Both female and male	FFQ	Nationally representative
Korea, Rep.	Korea National Health and Nutrition Examination Survey	GDD 2018	2001	2001	Both urban and rural	8008	11	85	Both female and male	FFQ	Nationally representative
Korea, Rep.	Korea National Health and Nutrition Examination Survey	GDD 2018	2005	2005	Both urban and rural	7415	11	85	Both female and male	FFQ	Nationally representative
Korea, Rep.	Korea National Health and Nutrition Examination Survey	GDD 2018	2007	2007	Both urban and rural	2893	11	85	Both female and male	FFQ	Nationally representative

Supplementary Data 1. Characteristics of surveys included in the modeling for sugar-sweetened beverages in the Global Dietary Database (continued).*

Country[f]	Survey Name	Data Collection Round	Year Survey Began	Year Survey Ended	Residence	Sample Size	Youngest Age Baseline	Oldest Age Baseline	Sex	Diet Assessment Method[‡]	Representativeness
Korea, Rep.	Korea National Health and Nutrition Examination Survey	GDD 2018	2008	2008	Both urban and rural	6518	11	85	Both female and male	FFQ	Nationally representative
Korea, Rep.	Korea National Health and Nutrition Examination Survey	GDD 2018	2009	2009	Both urban and rural	7264	11	85	Both female and male	FFQ	Nationally representative
Korea, Rep.	Korea National Health and Nutrition Examination Survey	GDD 2018	2010	2010	Both urban and rural	5930	11	85	Both female and male	FFQ	Nationally representative
Korea, Rep.	Korea National Health and Nutrition Examination Survey	GDD 2018	2011	2011	Both urban and rural	5778	11	85	Both female and male	FFQ	Nationally representative
Korea, Rep.	Korea National Health and Nutrition Examination Survey	GDD 2018	2012	2012	Both urban and rural	3417	15	55	Both female and male	FFQ	Nationally representative
Korea, Rep.	Korea National Health and Nutrition Examination Survey	GDD 2018	2013	2013	Both urban and rural	3354	15	55	Both female and male	FFQ	Nationally representative
Korea, Rep.	Korea National Health and Nutrition Examination Survey	GDD 2018	2014	2014	Both urban and rural	2947	15	55	Both female and male	FFQ	Nationally representative
Korea, Rep.	Korea National Health and Nutrition Examination Survey (KNHANES)	GDD 2018	2017	2017	Both urban and rural	6458	1	75	Both female and male	Single 24-hour recall	Nationally representative
Kuwait	Global Student Based Student Health Survey 2011	GDD 2018	2011	2011	Both urban and rural	2638	11	15	Both female and male	FFQ	Nationally representative
Lao PDR	Lao Food Consumption Survey	GDD 2018	2016	2017	Both urban and rural	2045	0	75	Both female and male	Single 24-hour recall	Nationally representative
Latvia	Health Behavior in School-Aged Children 1994	GDD 2018	1993	1994	Both urban and rural	3719	6	15	Both female and male	FFQ	Nationally representative
Latvia	Health Behavior in School-Aged Children 1998	GDD 2018	1998	1998	Both urban and rural	3668	6	15	Both female and male	FFQ	Nationally representative
Latvia	Health Behavior in School-Aged Children 2002	GDD 2018	2001	2002	Both urban and rural	3402	6	15	Both female and male	FFQ	Nationally representative
Latvia	Health Behavior in School-Aged Children 2006	GDD 2018	2006	2006	Both urban and rural	4199	11	15	Both female and male	FFQ	Nationally representative
Latvia	Health Behavior in School-Aged Children 2010	GDD 2018	2010	2010	Both urban and rural	4232	11	15	Both female and male	FFQ	Nationally representative
Latvia	Latvian Community Health and Nutrition Survey	GDD 2010	2002	2002	Not reported	3949	0	0	Not reported	Household availability/budget survey	Nationally representative

Supplementary Data 1. Characteristics of surveys included in the modeling for sugar-sweetened beverages in the Global Dietary Database (continued).*

Country[f]	Survey Name	Data Collection Round	Year Survey Began	Year Survey Ended	Residence	Sample Size	Youngest Age Baseline	Oldest Age Baseline	Sex	Diet Assessment Method[‡]	Representativeness
Latvia	Latvian Community Health and Nutrition Survey	GDD 2010	2003	2003	Not reported	3631	0	0	Not reported	Household availability/budget survey	Nationally representative
Latvia	Latvian Community Health and Nutrition Survey	GDD 2010	2004	2004	Not reported	3913	0	0	Not reported	Household availability/budget survey	Nationally representative
Lebanon	Global Student Based Student Health Survey 2011	GDD 2018	2011	2011	Both urban and rural	2258	11	15	Both female and male	FFQ	Nationally representative
Lebanon	Socioeconomic and dietary determinants of obesity in Lebanon	GDD 2018	2008	2009	Not reported	2992	7	85	Both female and male	FFQ	Nationally representative
Lithuania	Health Behavior in School-Aged Children 1994	GDD 2018	1993	1994	Both urban and rural	5419	6	15	Both female and male	FFQ	Nationally representative
Lithuania	Health Behavior in School-Aged Children 1998	GDD 2018	1998	1998	Both urban and rural	4504	6	15	Both female and male	FFQ	Nationally representative
Lithuania	Health Behavior in School-Aged Children 2002	GDD 2018	2001	2002	Both urban and rural	5627	6	15	Both female and male	FFQ	Nationally representative
Lithuania	Health Behavior in School-Aged Children 2006	GDD 2018	2006	2006	Both urban and rural	5577	11	15	Both female and male	FFQ	Nationally representative
Lithuania	Health Behavior in School-Aged Children 2010	GDD 2018	2010	2010	Both urban and rural	5287	11	15	Both female and male	FFQ	Nationally representative
Luxembourg	Health Behavior in School-Aged Children 2006	GDD 2018	2006	2006	Both urban and rural	4262	11	15	Both female and male	FFQ	Nationally representative
Luxembourg	Health Behavior in School-Aged Children 2010	GDD 2018	2010	2010	Both urban and rural	4042	11	15	Both female and male	FFQ	Nationally representative
Luxembourg	Luxembourgian Community Health and Nutrition Survey	GDD 2010	1993	1993	Not reported	3008	0	0	Not reported	Household availability/budget survey	Nationally representative
Macedonia, FYR	Health Behavior in School-Aged Children 2002	GDD 2018	2001	2002	Both urban and rural	3961	6	15	Both female and male	FFQ	Nationally representative
Macedonia, FYR	Health Behavior in School-Aged Children 2006	GDD 2018	2006	2006	Both urban and rural	5232	11	15	Both female and male	FFQ	Nationally representative
Macedonia, FYR	Health Behavior in School-Aged Children 2010	GDD 2018	2009	2010	Both urban and rural	3741	11	15	Both female and male	FFQ	Nationally representative

Supplementary Data 1. Characteristics of surveys included in the modeling for sugar-sweetened beverages in the Global Dietary Database (continued).*

Country[f]	Survey Name	Data Collection Round	Year Survey Began	Year Survey Ended	Residence	Sample Size	Youngest Age Baseline	Oldest Age Baseline	Sex	Diet Assessment Method‡	Representativeness
Malawi	Demographic and Health Survey Malawi 2010	GDD 2018	2010	2010	Both urban and rural	13962	0	2	Both female and male	DHS questionnaire	Nationally representative
Malaysia	Clustering of lifestyle risk factors and understanding its association with stress on health and wellbeing among school teachers in Malaysia (CLUSTer)	GDD 2018	2014	2015	Both urban and rural	4799	20	55	Both female and male	FFQ	Locally representative
Malaysia	Global Student Based Student Health Survey 2012	GDD 2018	2012	2012	Both urban and rural	25405	6	15	Both female and male	FFQ	Nationally representative
Malaysia	The Universiti Sains Malaysia Pregnancy Cohort Study	GDD 2018	2010	2015	Only rural	153	20	35	Only female	Single 24-hour recall	Locally representative
Maldives	Global Student Based Student Health Survey 2009	GDD 2018	2009	2009	Both urban and rural	3110	11	15	Both female and male	FFQ	Nationally representative
Malta	Health Behavior in School-Aged Children 2002	GDD 2018	2001	2002	Both urban and rural	1937	11	15	Both female and male	FFQ	Nationally representative
Malta	Health Behavior in School-Aged Children 2006	GDD 2018	2006	2006	Both urban and rural	1385	11	15	Both female and male	FFQ	Nationally representative
Malta	Maltese Community Health and Nutrition Survey	GDD 2010	1994	1994	Not reported	2722	0	0	Not reported	Household availability/budget survey	Nationally representative
Malta	Maltese Community Health and Nutrition Survey	GDD 2010	1995	1995	Not reported	2748	0	0	Not reported	Household availability/budget survey	Nationally representative
Malta	Maltese Community Health and Nutrition Survey	GDD 2010	2000	2000	Not reported	2586	0	0	Not reported	Household availability/budget survey	Nationally representative
Mauritania	Global Student Based Student Health Survey 2010	GDD 2018	2010	2010	Both urban and rural	1972	11	15	Both female and male	FFQ	Nationally representative
Mauritius	Dietary Habits and energy Intake- Mauritius population	GDD 2018	2013	2014	Not reported	509	11	11	Only female	FFQ	Sub-nationally representative
Mauritius	FOOD HABITS AND ENERGY INTAKE AMONG THE MAURITIAN POPULATION	GDD 2018	2014	2015	Not reported	351	20	45	Both female and male	FFQ	Nationally representative
Mauritius	Global Student Based Student Health Survey 2011	GDD 2018	2011	2011	Both urban and rural	2146	11	15	Both female and male	FFQ	Nationally representative

Supplementary Data 1. Characteristics of surveys included in the modeling for sugar-sweetened beverages in the Global Dietary Database (continued).*

Country[f]	Survey Name	Data Collection Round	Year Survey Began	Year Survey Ended	Residence	Sample Size	Youngest Age Baseline	Oldest Age Baseline	Sex	Diet Assessment Method[‡]	Representativeness
Mexico	ENSANUT	GDD 2018	2006	2006	Both urban and rural	24271	11	55	Both female and male	FFQ	Nationally representative
Mexico	National Health and Nutrition Survey (ENSANUT)	GDD 2018	2012	2012	Both urban and rural	10631	0	85	Both female and male	Multiple 24-hour recall	Nationally representative
Mexico	National Health and Nutrition Survey 2006 (Mexico)	GDD 2010	2005	2006	Not reported	15186	20	55	Both female and male	FFQ	Nationally representative
Mongolia	Global Student Based Student Health Survey 2013	GDD 2018	2013	2013	Both urban and rural	5347	6	15	Both female and male	FFQ	Nationally representative
Montenegro	Montenegrin Community Health and Nutrition Survey	GDD 2010	2003	2003	Not reported	380	0	0	Not reported	Household availability/budget survey	Nationally representative
Montenegro	Montenegrin Community Health and Nutrition Survey	GDD 2010	2004	2004	Not reported	380	0	0	Not reported	Household availability/budget survey	Nationally representative
Morocco	Arab Teens Lifestyle Study (ATLS)	GDD 2018	2011	2012	Not reported	610	15	20	Both female and male	FFQ	Locally representative
Morocco	Global Student Based Student Health Survey 2010	GDD 2018	2010	2010	Both urban and rural	2833	11	15	Both female and male	FFQ	Nationally representative
Mozambique	Food Consumption and Culinary Practices in Maputo, Mozambique	GDD 2018	2012	2013	Only urban	98	25	55	Both female and male	Single 24-hour recall	Locally representative
Mozambique	ZANE Study	GDD 2018	2010	2010	Both urban and rural	526	11	15	Only female	Multiple 24-hour recall	Sub-nationally representative
Namibia	Global Student Based Student Health Survey 2013	GDD 2018	2013	2013	Both urban and rural	4388	6	15	Both female and male	FFQ	Nationally representative
Netherlands	Diet of community-dwelling older adults 2010-2012	GDD 2018	2010	2012	Both urban and rural	1472	65	85	Both female and male	Multiple 24-hour recall	Nationally representative
Netherlands	Dutch National Consumption Survey-Young Children 2005/2006	GDD 2018	2005	2006	Both urban and rural	2558	2	6	Both female and male	Multiple 24-hour recall	Nationally representative
Netherlands	Dutch National Food Consumption Survey	GDD 2010	1987	1988	Not reported	4111	20	75	Both female and male	Multiple 24-hour recall	Nationally representative
Netherlands	Dutch National Food Consumption Survey	GDD 2010	1992	1992	Not reported	4447	20	85	Both female and male	Multiple 24-hour recall	Nationally representative
Netherlands	Dutch National Food Consumption Survey	GDD 2010	1997	1998	Not reported	4612	20	85	Both female and male	Multiple 24-hour recall	Nationally representative

Supplementary Data 1. Characteristics of surveys included in the modeling for sugar-sweetened beverages in the Global Dietary Database (continued).*

Country[f]	Survey Name	Data Collection Round	Year Survey Began	Year Survey Ended	Residence	Sample Size	Youngest Age Baseline	Oldest Age Baseline	Sex	Diet Assessment Method[‡]	Representativeness
Netherlands	Dutch National Food Consumption Survey	GDD 2010	2003	2003	Not reported	691	20	25	Both female and male	Multiple 24-hour recall	Nationally representative
Netherlands	Dutch National Food Consumption Survey 2007-2010 Diet of Children and adults aged 7-69 years	GDD 2018	2007	2010	Both urban and rural	4204	15	65	Both female and male	Multiple 24-hour recall	Nationally representative
Netherlands	Health Behavior in School-Aged Children 2002	GDD 2018	2001	2002	Both urban and rural	4240	11	15	Both female and male	FFQ	Nationally representative
Netherlands	Health Behavior in School-Aged Children 2006	GDD 2018	2006	2006	Both urban and rural	4208	11	15	Both female and male	FFQ	Nationally representative
Netherlands	Health Behavior in School-Aged Children 2010	GDD 2018	2010	2010	Both urban and rural	4501	11	15	Both female and male	FFQ	Nationally representative
New Zealand	NZ Childrens Nutrition Survey	GDD 2010	2002	2002	Not reported	3562	5	11	Both female and male	Multiple 24-hour recall	Nationally representative
New Zealand	NZ National Nutrition Survey 1997	GDD 2010	1997	1997	Not reported	4289	20	85	Both female and male	Multiple 24-hour recall	Nationally representative
Norway	Health Behavior in School-Aged Children 1990	GDD 2018	1989	1990	Both urban and rural	4956	6	15	Both female and male	FFQ	Nationally representative
Norway	Health Behavior in School-Aged Children 1994	GDD 2018	1993	1994	Both urban and rural	4842	6	15	Both female and male	FFQ	Nationally representative
Norway	Health Behavior in School-Aged Children 1998	GDD 2018	1998	1998	Both urban and rural	4960	6	15	Both female and male	FFQ	Nationally representative
Norway	Health Behavior in School-Aged Children 2002	GDD 2018	2001	2002	Both urban and rural	4950	6	15	Both female and male	FFQ	Nationally representative
Norway	Health Behavior in School-Aged Children 2006	GDD 2018	2006	2006	Both urban and rural	4663	11	15	Both female and male	FFQ	Nationally representative
Norway	Health Behavior in School-Aged Children 2010	GDD 2018	2010	2010	Both urban and rural	4304	11	15	Both female and male	FFQ	Nationally representative
Norway	Norwegian Community Health and Nutrition Survey	GDD 2010	1986	1988	Not reported	7724	0	0	Not reported	Household availability/budget survey	Nationally representative
Norway	Norwegian Community Health and Nutrition Survey	GDD 2010	1992	1994	Not reported	6518	0	0	Not reported	Household availability/budget survey	Nationally representative

Supplementary Data 1. Characteristics of surveys included in the modeling for sugar-sweetened beverages in the Global Dietary Database (continued).*

Country[f]	Survey Name	Data Collection Round	Year Survey Began	Year Survey Ended	Residence	Sample Size	Youngest Age Baseline	Oldest Age Baseline	Sex	Diet Assessment Method‡	Representativeness
Norway	Norwegian Community Health and Nutrition Survey	GDD 2010	1996	1998	Not reported	6504	0	0	Not reported	Household availability/budget survey	Nationally representative
Norway	Norwegian national dietary survey 1997 (Norkost 1997)	GDD 2010	1997	1997	Not reported	2494	20	75	Both female and male	FFQ	Nationally representative
Oman	Global Student Based Student Health Survey 2010	GDD 2018	2010	2010	Both urban and rural	1523	11	15	Both female and male	FFQ	Nationally representative
Pakistan	Global Student Based Student Health Survey 2009	GDD 2018	2009	2009	Both urban and rural	5146	11	15	Both female and male	FFQ	Nationally representative
Palestine	Global Student Based Student Health Survey 2010	GDD 2018	2010	2010	Both urban and rural	8507	11	15	Both female and male	FFQ	Nationally representative
Peru	Global Student Based Student Health Survey 2010	GDD 2018	2010	2010	Both urban and rural	2851	11	15	Both female and male	FFQ	Nationally representative
Philippines	Cebu Longitudinal Health and Nutrition Survey (CLHNS)	GDD 2018	2002	2002	Both urban and rural	2437	15	55	Both female and male	Multiple 24-hour recall	Sub-nationally representative
Philippines	Cebu Longitudinal Health and Nutrition Survey (CLHNS)	GDD 2018	2005	2005	Both urban and rural	3929	20	65	Both female and male	Multiple 24-hour recall	Sub-nationally representative
Philippines	Global Student Based Student Health Survey 2011	GDD 2018	2011	2011	Both urban and rural	5211	11	15	Both female and male	FFQ	Nationally representative
Poland	Health Behavior in School-Aged Children 1990	GDD 2018	1989	1990	Both urban and rural	4394	6	15	Both female and male	FFQ	Nationally representative
Poland	Health Behavior in School-Aged Children 1994	GDD 2018	1993	1994	Both urban and rural	4310	6	15	Both female and male	FFQ	Nationally representative
Poland	Health Behavior in School-Aged Children 1998	GDD 2018	1998	1998	Both urban and rural	4828	6	15	Both female and male	FFQ	Nationally representative
Poland	Health Behavior in School-Aged Children 2002	GDD 2018	2001	2002	Both urban and rural	6283	11	15	Both female and male	FFQ	Nationally representative
Poland	Health Behavior in School-Aged Children 2006	GDD 2018	2006	2006	Both urban and rural	5471	11	15	Both female and male	FFQ	Nationally representative
Poland	Health Behavior in School-Aged Children 2010	GDD 2018	2010	2010	Both urban and rural	4213	11	15	Both female and male	FFQ	Nationally representative
Poland	Household Food Consumption and Anthropometric Survey	GDD 2018	2000	2000	Both urban and rural	3973	2	85	Both female and male	Single 24-hour recall	Nationally representative

Supplementary Data 1. Characteristics of surveys included in the modeling for sugar-sweetened beverages in the Global Dietary Database (continued).*

Country[f]	Survey Name	Data Collection Round	Year Survey Began	Year Survey Ended	Residence	Sample Size	Youngest Age Baseline	Oldest Age Baseline	Sex	Diet Assessment Method‡	Representativeness
Poland	Household Food Consumption and Anthropometric Survey	GDD 2010	2000	2000	Not reported	4134	0	85	Both female and male	Single 24-hour recall	Nationally representative
Poland	Pol-MONICA bis Warsaw Project	GDD 2010	2001	2001	Not reported	1329	20	65	Both female and male	Single 24-hour recall	Sub-nationally representative
Poland	Polish Community Health and Nutrition Survey	GDD 2010	1988	1988	Not reported	29664	0	0	Not reported	Household availability/budget survey	Nationally representative
Poland	WOBASZ - National Multicenter Health Survey	GDD 2010	2003	2005	Not reported	6661	20	65	Both female and male	Single 24-hour recall	Nationally representative
Poland	WOBASZ II	GDD 2018	2013	2014	Both urban and rural	5678	15	85	Both female and male	Single 24-hour recall	Nationally representative
Portugal	Health Behavior in School-Aged Children 1998	GDD 2018	1998	1998	Both urban and rural	3627	11	15	Both female and male	FFQ	Nationally representative
Portugal	Health Behavior in School-Aged Children 2002	GDD 2018	2001	2002	Both urban and rural	2915	11	15	Both female and male	FFQ	Nationally representative
Portugal	Health Behavior in School-Aged Children 2006	GDD 2018	2006	2006	Both urban and rural	3887	11	15	Both female and male	FFQ	Nationally representative
Portugal	Health Behavior in School-Aged Children 2010	GDD 2018	2010	2010	Both urban and rural	4000	11	15	Both female and male	FFQ	Nationally representative
Portugal	National Food, Nutrition, and Physical Activity Survey of the Portuguese General Population (IAN-AF)	GDD 2018	2015	2016	Both urban and rural	5793	0	75	Both female and male	Multiple 24-hour recall	Nationally representative
Portugal	National food and physical activity survey (IAN-AF) - Pregnant women	GDD 2018	2015	2016	Not reported	155	15	35	Only female	Multiple 24-hour recall	Nationally representative
Portugal	Portuguese Community Health and Nutrition Survey	GDD 2010	1990	1990	Not reported	12403	0	0	Not reported	Household availability/budget survey	Nationally representative
Portugal	Portuguese Community Health and Nutrition Survey	GDD 2010	1995	1995	Not reported	10554	0	0	Not reported	Household availability/budget survey	Nationally representative
Portugal	Portuguese Community Health and Nutrition Survey	GDD 2010	2000	2000	Not reported	10020	0	0	Not reported	Household availability/budget survey	Nationally representative

Supplementary Data 1. Characteristics of surveys included in the modeling for sugar-sweetened beverages in the Global Dietary Database (continued).*

Country[ƒ]	Survey Name	Data Collection Round	Year Survey Began	Year Survey Ended	Residence	Sample Size	Youngest Age Baseline	Oldest Age Baseline	Sex	Diet Assessment Method[‡]	Representativeness
Qatar	Global Student Based Student Health Survey 2011	GDD 2018	2011	2011	Both urban and rural	1936	11	15	Both female and male	FFQ	Nationally representative
Romania	DIETA PILOT Adults	GDD 2018	2012	2012	Not reported	1362	20	85	Both female and male	Multiple food record	Nationally representative
Romania	DIETA PILOT Children	GDD 2018	2012	2012	Not reported	272	15	15	Both female and male	Single 24-hour recall	Nationally representative
Romania	Health Behavior in School-Aged Children 2006	GDD 2018	2006	2006	Both urban and rural	4587	11	15	Both female and male	FFQ	Nationally representative
Romania	Health Behavior in School-Aged Children 2010	GDD 2018	2010	2010	Both urban and rural	5257	11	15	Both female and male	FFQ	Nationally representative
Russian Federation	Health Behavior in School-Aged Children 1994	GDD 2018	1993	1994	Both urban and rural	4001	6	15	Both female and male	FFQ	Nationally representative
Russian Federation	Health Behavior in School-Aged Children 1998	GDD 2018	1998	1998	Both urban and rural	3991	6	15	Both female and male	FFQ	Nationally representative
Russian Federation	Health Behavior in School-Aged Children 2002	GDD 2018	2001	2002	Both urban and rural	8025	11	15	Both female and male	FFQ	Nationally representative
Russian Federation	Health Behavior in School-Aged Children 2006	GDD 2018	2006	2006	Both urban and rural	8113	11	15	Both female and male	FFQ	Nationally representative
Russian Federation	Health Behavior in School-Aged Children 2010	GDD 2018	2010	2010	Both urban and rural	5100	11	15	Both female and male	FFQ	Nationally representative
Samoa	Global Student Based Student Health Survey 2011	GDD 2018	2011	2011	Both urban and rural	2279	11	15	Both female and male	FFQ	Nationally representative
Serbia	Serbian Community Health and Nutrition Survey	GDD 2010	2003	2003	Not reported	4800	0	0	Not reported	Household availability/budget survey	Nationally representative
Serbia	Serbian Community Health and Nutrition Survey	GDD 2010	2004	2004	Not reported	4800	0	0	Not reported	Household availability/budget survey	Nationally representative
Seychelles	Seychelles Heart Survey	GDD 2018	2013	2015	Both urban and rural	1236	25	55	Both female and male	FFQ	Nationally representative
Singapore	National Nutrition Survey 1998	GDD 2010	1998	1998	Not reported	2270	20	65	Both female and male	FFQ	Nationally representative
Singapore	National Nutrition Survey 2004	GDD 2010	2004	2004	Not reported	1314	20	65	Both female and male	FFQ	Nationally representative

Supplementary Data 1. Characteristics of surveys included in the modeling for sugar-sweetened beverages in the Global Dietary Database (continued).*

Country[f]	Survey Name	Data Collection Round	Year Survey Began	Year Survey Ended	Residence	Sample Size	Youngest Age Baseline	Oldest Age Baseline	Sex	Diet Assessment Method[‡]	Representativeness
Slovak Republic	Compilation of existing individual food consumption data collected within the most recent national dietary surveys in Europe (SK MON)	GDD 2018	2008	2008	Both urban and rural	2759	15	55	Both female and male	Single 24-hour recall	Nationally representative
Slovak Republic	Health Behavior in School-Aged Children 1994	GDD 2018	1993	1994	Both urban and rural	3350	6	15	Both female and male	FFQ	Nationally representative
Slovak Republic	Health Behavior in School-Aged Children 1998	GDD 2018	1998	1998	Both urban and rural	3777	11	15	Both female and male	FFQ	Nationally representative
Slovak Republic	Health Behavior in School-Aged Children 2006	GDD 2018	2006	2006	Both urban and rural	3822	11	15	Both female and male	FFQ	Nationally representative
Slovak Republic	Health Behavior in School-Aged Children 2010	GDD 2018	2010	2010	Both urban and rural	5165	11	15	Both female and male	FFQ	Nationally representative
Slovak Republic	Monitoring of the nutritional status of particular groups of adult population	GDD 2010	2006	2006	Not reported	2208	19	19	Not reported	Single 24-hour recall	Nationally representative
Slovak Republic	Slovakian Community Health and Nutrition Survey	GDD 2010	1997	1997	Not reported	1671	0	0	Not reported	Household availability/budget survey	Nationally representative
Slovak Republic	Slovakian Community Health and Nutrition Survey	GDD 2010	2000	2000	Not reported	1647	0	0	Not reported	Household availability/budget survey	Nationally representative
Slovak Republic	Slovakian Community Health and Nutrition Survey	GDD 2010	2003	2003	Not reported	1645	0	0	Not reported	Household availability/budget survey	Nationally representative
Slovenia	Health Behavior in School-Aged Children 2002	GDD 2018	2001	2002	Both urban and rural	3904	11	15	Both female and male	FFQ	Nationally representative
Slovenia	Health Behavior in School-Aged Children 2006	GDD 2018	2006	2006	Both urban and rural	5089	11	15	Both female and male	FFQ	Nationally representative
Slovenia	Health Behavior in School-Aged Children 2010	GDD 2018	2010	2010	Both urban and rural	5409	11	15	Both female and male	FFQ	Nationally representative
Slovenia	Slovenian Community Health and Nutrition Survey	GDD 2010	1998	1998	Not reported	4979	0	0	Not reported	Household availability/budget survey	Nationally representative

Supplementary Data 1. Characteristics of surveys included in the modeling for sugar-sweetened beverages in the Global Dietary Database (continued).*

Country[f]	Survey Name	Data Collection Round	Year Survey Began	Year Survey Ended	Residence	Sample Size	Youngest Age Baseline	Oldest Age Baseline	Sex	Diet Assessment Method‡	Representativeness
Slovenia	Slovenian Community Health and Nutrition Survey	GDD 2010	2000	2000	Not reported	4904	0	0	Not reported	Household availability/budget survey	Nationally representative
Slovenia	Slovenian Community Health and Nutrition Survey	GDD 2010	2002	2002	Not reported	3687	0	0	Not reported	Household availability/budget survey	Nationally representative
Solomon Islands	Global Student Based Student Health Survey 2011	GDD 2018	2011	2011	Both urban and rural	1310	11	15	Both female and male	FFQ	Nationally representative
South Africa	Food and nutrient availability in South African Households	GDD 2010	1995	1995	Not reported	1502	0	85	Both female and male	FFQ	Nationally representative
Spain	Health Behavior in School-Aged Children 1994	GDD 2018	1993	1994	Both urban and rural	4540	11	15	Both female and male	FFQ	Nationally representative
Spain	Health Behavior in School-Aged Children 2002	GDD 2018	2001	2002	Both urban and rural	5808	6	15	Both female and male	FFQ	Nationally representative
Spain	Health Behavior in School-Aged Children 2006	GDD 2018	2005	2006	Both urban and rural	8849	11	15	Both female and male	FFQ	Nationally representative
Spain	Health Behavior in School-Aged Children 2010	GDD 2018	2009	2010	Both urban and rural	5025	11	15	Both female and male	FFQ	Nationally representative
Spain	Spanish Community Health and Nutrition Survey	GDD 2010	1980	1981	Not reported	30331	0	0	Not reported	Household availability/budget survey	Nationally representative
Spain	Spanish Community Health and Nutrition Survey	GDD 2010	1990	1991	Not reported	30331	0	0	Not reported	Household availability/budget survey	Nationally representative
Spain	Spanish Community Health and Nutrition Survey	GDD 2010	1998	1999	Not reported	14644	0	0	Not reported	Household availability/budget survey	Nationally representative
Sri Lanka	Abdominal obesity and its association with selected risk factors of coronary heart disease in an adult population in the district of Colombo; MD Thesis (Community Medicine), Postgraduate Institute of Medicine, University of Colombo, Sri Lanka	GDD 2010	2004	2004	Not reported	1400	20	55	Both female and male	FFQ	Sub-nationally representative

Supplementary Data 1. Characteristics of surveys included in the modeling for sugar-sweetened beverages in the Global Dietary Database (continued).*

Country^f	Survey Name	Data Collection Round	Year Survey Began	Year Survey Ended	Residence	Sample Size	Youngest Age Baseline	Oldest Age Baseline	Sex	Diet Assessment Method‡	Representativeness
Sudan	Global Student Based Student Health Survey 2012	GDD 2018	2012	2012	Both urban and rural	2084	11	15	Both female and male	FFQ	Nationally representative
Suriname	Global Student Based Student Health Survey 2009	GDD 2018	2009	2009	Both urban and rural	1661	11	15	Both female and male	FFQ	Nationally representative
Swaziland	Global Student Based Student Health Survey 2013	GDD 2018	2013	2013	Both urban and rural	3590	6	15	Both female and male	FFQ	Nationally representative
Sweden	Dietary habits and nutrient intake among Swedish children	GDD 2010	2003	2003	Not reported	2495	0	11	Both female and male	Single 24-hour recall	Nationally representative
Sweden	Dietary habits and nutrient intake in Sweden 1989	GDD 2010	1989	1989	Not reported	1573	18	65	Both female and male	Single 24-hour recall	Nationally representative
Sweden	Dietary habits and nutrient intake in Sweden 1997-98	GDD 2010	1997	1998	Not reported	1208	17	65	Both female and male	Single 24-hour recall	Nationally representative
Sweden	Health Behavior in School-Aged Children 1986	GDD 2018	1985	1986	Both urban and rural	2917	11	15	Both female and male	FFQ	Nationally representative
Sweden	Health Behavior in School-Aged Children 1990	GDD 2018	1989	1990	Both urban and rural	3445	6	15	Both female and male	FFQ	Nationally representative
Sweden	Health Behavior in School-Aged Children 1994	GDD 2018	1993	1994	Both urban and rural	3522	11	15	Both female and male	FFQ	Nationally representative
Sweden	Health Behavior in School-Aged Children 1998	GDD 2018	1997	1998	Both urban and rural	3760	6	15	Both female and male	FFQ	Nationally representative
Sweden	Health Behavior in School-Aged Children 2002	GDD 2018	2001	2002	Both urban and rural	3880	6	15	Both female and male	FFQ	Nationally representative
Sweden	Health Behavior in School-Aged Children 2006	GDD 2018	2005	2006	Both urban and rural	4287	11	15	Both female and male	FFQ	Nationally representative
Sweden	Health Behavior in School-Aged Children 2010	GDD 2018	2009	2010	Both urban and rural	6524	11	15	Both female and male	FFQ	Nationally representative
Sweden	Swedish Community Health and Nutrition Survey	GDD 2010	1989	1989	Not reported	2970	0	0	Not reported	Household availability/budget survey	Nationally representative
Sweden	Swedish Community Health and Nutrition Survey	GDD 2010	1996	1996	Not reported	2026	18	18	Not reported	Household availability/budget survey	Nationally representative
Sweden	Swedish National Dietary Survey-Riksmaten 2010-11	GDD 2018	2010	2011	Both urban and rural	1792	15	75	Both female and male	Multiple 24-hour recall	Nationally representative

Supplementary Data 1. Characteristics of surveys included in the modeling for sugar-sweetened beverages in the Global Dietary Database (continued).*

Country[f]	Survey Name	Data Collection Round	Year Survey Began	Year Survey Ended	Residence	Sample Size	Youngest Age Baseline	Oldest Age Baseline	Sex	Diet Assessment Method[‡]	Representativeness
Switzerland	Bus Santè	GDD 2018	1994	2012	Not reported	4842	35	65	Both female and male	FFQ	Sub-nationally representative
Switzerland	CoLaus	GDD 2018	2009	2012	Not reported	4249	35	65	Both female and male	FFQ	Locally representative
Switzerland	Etude Bus Santè	GDD 2010	2004	2009	Not reported	3319	35	65	Both female and male	FFQ	Locally representative
Switzerland	Health Behavior in School-Aged Children 1998	GDD 2018	1997	1998	Both urban and rural	5512	11	15	Both female and male	FFQ	Nationally representative
Switzerland	Health Behavior in School-Aged Children 2002	GDD 2018	2001	2002	Both urban and rural	4518	6	15	Both female and male	FFQ	Nationally representative
Switzerland	Health Behavior in School-Aged Children 2006	GDD 2018	2005	2006	Both urban and rural	4551	11	15	Both female and male	FFQ	Nationally representative
Switzerland	Health Behavior in School-Aged Children 2010	GDD 2018	2009	2010	Both urban and rural	6597	11	15	Both female and male	FFQ	Nationally representative
Syrian Arab Republic	Global Student Based Student Health Survey 2010	GDD 2018	2010	2010	Both urban and rural	2079	11	15	Both female and male	FFQ	Nationally representative
Syrian Arab Republic	Global Student Based Student Health Survey 2011	GDD 2018	2011	2011	Both urban and rural	3071	11	15	Both female and male	FFQ	Nationally representative
Taiwan	2005-2008 Nutrition and Health Survey in Taiwan	GDD 2010	2005	2008	Not reported	2908	19	85	Both female and male	Single 24-hour recall	Nationally representative
Thailand	National Food and Nutrition Survey, Thailand	GDD 2010	2003	2003	Not reported	1610	15	60	Not reported	Single 24-hour recall	Nationally representative
Tonga	Global Student Based Student Health Survey 2010	GDD 2018	2010	2010	Both urban and rural	2167	11	15	Both female and male	FFQ	Nationally representative
Trinidad and Tobago	Global Student Based Student Health Survey 2011	GDD 2018	2011	2011	Both urban and rural	2731	11	15	Both female and male	FFQ	Nationally representative
Turkey	Determination of dietary habits as a risk factor of cardiovascular heart disease in Turkish adolescents	GDD 2018	2003	2004	Only urban	300	11	15	Both female and male	Multiple 24-hour recall	Locally representative
Turkey	Dietary intake of adult population living in Ankara	GDD 2010	2005	2005	Not reported	1484	20	75	Both female and male	Single 24-hour recall	Locally representative
Turkey	Health Behavior in School-Aged Children 2006	GDD 2018	2005	2006	Both urban and rural	5380	11	15	Both female and male	FFQ	Nationally representative

Supplementary Data 1. Characteristics of surveys included in the modeling for sugar-sweetened beverages in the Global Dietary Database (continued).*

Country^f	Survey Name	Data Collection Round	Year Survey Began	Year Survey Ended	Residence	Sample Size	Youngest Age Baseline	Oldest Age Baseline	Sex	Diet Assessment Method‡	Representativeness
Turkey	Health Behavior in School-Aged Children 2010	GDD 2018	2009	2010	Both urban and rural	5545	11	15	Both female and male	FFQ	Nationally representative
Ukraine	Health Behavior in School-Aged Children 2002	GDD 2018	2001	2002	Both urban and rural	4061	6	15	Both female and male	FFQ	Nationally representative
Ukraine	Health Behavior in School-Aged Children 2006	GDD 2018	2005	2006	Both urban and rural	5015	11	15	Both female and male	FFQ	Nationally representative
Ukraine	Health Behavior in School-Aged Children 2010	GDD 2018	2009	2010	Both urban and rural	5820	11	15	Both female and male	FFQ	Nationally representative
United Arab Emirates	Global Student Based Student Health Survey 2010	GDD 2018	2010	2010	Both urban and rural	2524	11	15	Both female and male	FFQ	Nationally representative
United Kingdom	Dietary and Nutritional Survey of British Adults	GDD 2010	1986	1987	Not reported	2197	16	50	Both female and male	Single 24-hour recall	Nationally representative
United Kingdom	Health Behavior in School-Aged Children 1986	GDD 2018	1985	1986	Both urban and rural	4757	11	15	Both female and male	FFQ	Nationally representative
United Kingdom	Health Behavior in School-Aged Children 1990	GDD 2018	1989	1990	Both urban and rural	6674	11	15	Both female and male	FFQ	Sub-nationally representative
United Kingdom	Health Behavior in School-Aged Children 1994	GDD 2018	1993	1994	Both urban and rural	3933	11	15	Both female and male	FFQ	Sub-nationally representative
United Kingdom	Health Behavior in School-Aged Children 1994	GDD 2018	1993	1994	Both urban and rural	4871	6	15	Both female and male	FFQ	Sub-nationally representative
United Kingdom	Health Behavior in School-Aged Children 1994	GDD 2018	1993	1994	Both urban and rural	3827	11	15	Both female and male	FFQ	Sub-nationally representative
United Kingdom	Health Behavior in School-Aged Children 1998	GDD 2018	1997	1998	Both urban and rural	6315	6	15	Both female and male	FFQ	Sub-nationally representative
United Kingdom	Health Behavior in School-Aged Children 1998	GDD 2018	1997	1998	Both urban and rural	3331	11	15	Both female and male	FFQ	Sub-nationally representative
United Kingdom	Health Behavior in School-Aged Children 1998	GDD 2018	1997	1998	Both urban and rural	5580	6	15	Both female and male	FFQ	Sub-nationally representative
United Kingdom	Health Behavior in School-Aged Children 1998	GDD 2018	1997	1998	Both urban and rural	4117	11	15	Both female and male	FFQ	Sub-nationally representative
United Kingdom	Health Behavior in School-Aged Children 2002	GDD 2018	2001	2002	Both urban and rural	14260	6	15	Both female and male	FFQ	Sub-nationally representative
United Kingdom	Health Behavior in School-Aged Children 2006	GDD 2018	2005	2006	Both urban and rural	15234	11	15	Both female and male	FFQ	Nationally representative

Supplementary Data 1. Characteristics of surveys included in the modeling for sugar-sweetened beverages in the Global Dietary Database (continued).*

Country[f]	Survey Name	Data Collection Round	Year Survey Began	Year Survey Ended	Residence	Sample Size	Youngest Age Baseline	Oldest Age Baseline	Sex	Diet Assessment Method[‡]	Representativeness
United Kingdom	Health Behavior in School-Aged Children 2010	GDD 2018	2009	2010	Both urban and rural	15541	11	15	Both female and male	FFQ	Nationally representative
United Kingdom	National Diet and Nutrition Survey: adults aged 19-64 years	GDD 2010	2000	2001	Not reported	1724	19	50	Both female and male	Single 24-hour recall	Nationally representative
United Kingdom	National Diet and Nutrition Survey: children aged 1-4 years	GDD 2010	1992	1993	Not reported	1675	2	2	Both female and male	Single 24-hour recall	Nationally representative
United Kingdom	National Diet and Nutrition Survey: people aged 65 years and over	GDD 2010	1994	1995	Not reported	1275	65	85	Both female and male	Single 24-hour recall	Nationally representative
United Kingdom	National Diet and Nutrition Survey: young people 4-18 years	GDD 2010	1997	1997	Not reported	1601	4	15	Both female and male	Single 24-hour recall	Nationally representative
United States	Continuing Survey of Food Intakes by Individuals	GDD 2010	1989	1989	Not reported	5204	0	85	Both female and male	Multiple 24-hour recall	Nationally representative
United States	Continuing Survey of Food Intakes by Individuals	GDD 2010	1990	1990	Not reported	4956	0	85	Both female and male	Multiple 24-hour recall	Nationally representative
United States	Continuing Survey of Food Intakes by Individuals	GDD 2010	1991	1991	Not reported	5238	0	85	Both female and male	Multiple 24-hour recall	Nationally representative
United States	Continuing Survey of Food Intakes by Individuals	GDD 2010	1994	1994	Not reported	5589	0	85	Both female and male	Multiple 24-hour recall	Nationally representative
United States	Continuing Survey of Food Intakes by Individuals	GDD 2010	1995	1995	Not reported	5326	0	85	Both female and male	Multiple 24-hour recall	Nationally representative
United States	Continuing Survey of Food Intakes by Individuals	GDD 2010	1996	1996	Not reported	5188	0	85	Both female and male	Multiple 24-hour recall	Nationally representative
United States	Health Behavior in School-Aged Children 1998	GDD 2018	1997	1998	Both urban and rural	5164	11	15	Both female and male	FFQ	Nationally representative
United States	Health Behavior in School-Aged Children 2002	GDD 2018	2001	2002	Both urban and rural	4957	11	15	Both female and male	FFQ	Nationally representative
United States	Health Behavior in School-Aged Children 2006	GDD 2018	2005	2006	Both urban and rural	3858	11	15	Both female and male	FFQ	Nationally representative
United States	Health Behavior in School-Aged Children 2010	GDD 2018	2009	2010	Both urban and rural	6037	11	15	Both female and male	FFQ	Nationally representative
United States	NHANES 1999-2000	GDD 2018	1999	2000	Not reported	7866	2	85	Both female and male	Multiple 24-hour recall	Nationally representative
United States	NHANES 2001-2002	GDD 2018	2001	2002	Not reported	8874	2	85	Both female and male	Multiple 24-hour recall	Nationally representative

Supplementary Data 1. Characteristics of surveys included in the modeling for sugar-sweetened beverages in the Global Dietary Database (continued).*

Country[f]	Survey Name	Data Collection Round	Year Survey Began	Year Survey Ended	Residence	Sample Size	Youngest Age Baseline	Oldest Age Baseline	Sex	Diet Assessment Method‡	Representativeness
United States	NHANES 2003-2004	GDD 2018	2003	2004	Not reported	7506	2	85	Both female and male	Multiple 24-hour recall	Nationally representative
United States	NHANES 2005-2006	GDD 2018	2005	2006	Not reported	8217	0	85	Both female and male	Multiple 24-hour recall	Nationally representative
United States	NHANES 2007-2008	GDD 2018	2007	2008	Not reported	7699	0	75	Both female and male	Multiple 24-hour recall	Nationally representative
United States	NHANES 2009-2010	GDD 2018	2009	2010	Not reported	8278	0	75	Both female and male	Multiple 24-hour recall	Nationally representative
United States	NHANES 2011-2012	GDD 2018	2011	2012	Not reported	7451	0	75	Both female and male	Multiple 24-hour recall	Nationally representative
United States	NHANES 2013-2014	GDD 2018	2013	2014	Not reported	7460	0	75	Both female and male	Multiple 24-hour recall	Nationally representative
United States	NHANES 2015-2016	GDD 2018	2015	2016	Not reported	6880	0	75	Both female and male	Multiple 24-hour recall	Nationally representative
United States	NHANES 2017-2018	GDD 2018	2017	2018	Not reported	6462	0	75	Both female and male	Multiple 24-hour recall	Nationally representative
Uruguay	Global Student Based Student Health Survey 2012	GDD 2018	2012	2012	Both urban and rural	3456	11	15	Both female and male	FFQ	Nationally representative
Vanuatu	Global Student Based Student Health Survey 2011	GDD 2018	2011	2011	Both urban and rural	1082	11	15	Both female and male	FFQ	Nationally representative
Vietnam	Global Student Based Student Health Survey 2013	GDD 2018	2013	2013	Both urban and rural	3305	6	15	Both female and male	FFQ	Nationally representative
Vietnam	Qualitative and quantitative assessment of nutritional status and lifestyles of Vietnamese adolescents	GDD 2018	2006	2006	Both urban and rural	780	15	15	Both female and male	Single 24-hour recall	Sub-nationally representative
Yemen, Rep.	Demographic and Health Survey Yemen 1991	GDD 2018	1991	1992	Both urban and rural	39	0	2	Both female and male	DHS questionnaire	Nationally representative

*Includes survey characteristics for the 451 surveys with sugar-sweetened beverage intake data included in the GDD model.
[f]Countries are ordered alphabetically.
‡ FFQ: Food Frequency Questionnaires; DHS: Demographic and Health Survey
