# Supplementary material for: Sugar-sweetened beverage intakes among adults between 1990 and 2018 in 185 countries
Source: Nat Commun. 2023 Oct 3;14:5957. doi: 10.1038/s41467-023-41269-8 (PMC10614169; doi:10.1038/s41467-023-41269-8)
Supplement: Supplementary file 5 — Supplementary Data 2 [file 41467_2023_41269_MOESM5_ESM.pdf]

Sugar-sweetened beverage intakes among adults between 1990 and 2018 in 185 countries

Supplementary Data 2 | Mean Intakes and Absolute Change by World Region and 185 Countries

Supplementary Data 2. Sugar-sweetened beverage intakes (8 oz servings/week) in 1990, 2005, and 2018 and absolute change (8 oz serving/week) from 1990-2005, 2005-2018, and 1990-2018 in adults (20+years) globally, regionally, and nationally.*

	Mean intake (95% UI) (8 oz servings/week)			Absolute change (95% UI) (8 oz servings/week)		
	1990	2005	2018	1990-2005	2005-2018	1990-2018
World	2.3 (2.2-2.5)	2.6 (2.4-2.7)	2.7 (2.5-2.9)	0.22 (0.17,0.28)	0.15 (0.11,0.21)	0.37 (0.29,0.47)
World Region						
Central Eastern Europe and Central Asia†	1.8 (1.6-2.1)	2.2 (1.9-2.5)	2.2 (1.9-2.5)	0.25 (0.11,0.39)	0.11 (0.05,0.18)	0.36 (0.23,0.49)
High-Income Countries	4.0 (3.8-4.2)	4.7 (4.5-4.9)	3.7 (3.5-3.9)	1.02 (0.94,1.10)	-1.11 (-1.19,-1.03)	-0.09 (-0.11,-0.07)
Latin America and Caribbean	8.7 (8.0-9.5)	7.4 (6.7-8.1)	7.8 (7.1-8.6)	-1.23 (-1.43,-1.03)	0.69 (0.51,0.88)	-0.54 (-0.76,-0.34)
Middle East and North Africa	4.1 (3.6-4.8)	4.5 (3.8-5.2)	4.6 (3.9-5.4)	0.46 (0.29,0.66)	-0.05 (-0.18,0.07)	0.41 (0.25,0.59)
South Asia†	0.6 (0.4-0.8)	0.8 (0.6-1.2)	0.7 (0.5-1.1)	0.07 (0.05,0.09)	-0.01 (-0.03,0.01)	0.05 (0.03,0.09)
Southeast and East Asia	0.8 (0.7-0.9)	0.8 (0.8-1.0)	0.9 (0.8-1.1)	0.08 (0.05,0.14)	0.11 (0.08,0.19)	0.19 (0.12,0.33)
Sub-Saharan Africa	3.6 (2.9-4.3)	5.0 (3.9-6.4)	6.6 (5.3-8.3)	1.04 (0.65,1.63)	1.93 (1.53,2.37)	2.99 (2.26,3.89)
Country						
Afghanistan	0.2 (0.1-0.3)	1.0 (0.5-1.9)	2.0 (1.0-3.9)	0.72 (0.36,1.50)	1.05 (0.48,2.22)	1.79 (0.91,3.56)
Albania	1.5 (1.0-2.2)	2.0 (1.4-2.9)	22.9 (18.3-27.2)	0.65 (0.31,1.16)	20.90 (16.74,24.68)	21.60 (17.35,25.56)
Algeria	6.4 (4.4-9.6)	5.5 (3.7-8.1)	5.8 (3.9-8.6)	-0.73 (-1.09,-0.50)	0.58 (0.38,0.87)	-0.16 (-0.23,-0.11)
Angola	4.0 (2.8-5.9)	6.3 (4.3-9.2)	8.1 (5.5-11.7)	1.60 (0.29,3.31)	1.96 (0.35,3.98)	3.57 (1.95,5.75)
Antigua and Barbuda	9.3 (6.2-14.1)	6.7 (4.5-10.1)	10.0 (6.7-15.2)	-2.32 (-3.50,-1.55)	3.69 (2.46,5.61)	1.37 (0.92,2.09)
Argentina	3.8 (3.2-4.6)	6.0 (5.1-7.2)	5.6 (4.7-6.7)	2.13 (1.78,2.54)	-0.34 (-0.40,-0.28)	1.79 (1.50,2.14)
Armenia	0.6 (0.4-0.9)	1.5 (1.0-2.2)	1.6 (1.1-2.4)	0.96 (0.65,1.39)	0.14 (0.09,0.20)	1.09 (0.74,1.60)
Australia	4.7 (3.8-5.7)	3.8 (3.1-4.6)	3.1 (2.5-3.8)	-0.64 (-0.79,-0.51)	-0.67 (-0.83,-0.54)	-1.31 (-1.62,-1.06)
Austria	2.9 (2.5-3.3)	2.8 (2.5-3.2)	2.7 (2.4-3.1)	0.13 (0.08,0.19)	-0.10 (-0.13,-0.08)	0.03 (0.00,0.06)
Azerbaijan	1.0 (0.7-1.5)	1.9 (1.3-2.9)	1.3 (0.9-1.9)	1.00 (0.60,1.59)	-0.60 (-1.12,-0.22)	0.40 (0.14,0.75)
Bahamas, The	23.6 (15.3-32.1)	24.7 (16.1-32.8)	5.8 (3.7-8.7)	1.69 (1.15,1.96)	-18.06 (-23.49,-11.74)	-16.26 (-22.10,-10.55)
Bahrain	5.0 (3.5-7.5)	6.4 (4.4-9.4)	5.9 (4.1-8.7)	1.49 (-0.07,3.38)	-0.45 (-2.28,1.27)	1.05 (-0.49,2.79)
Bangladesh	0.2 (0.1-0.3)	0.3 (0.2-0.4)	0.2 (0.2-0.3)	0.02 (0.01,0.04)	-0.04 (-0.06,-0.02)	-0.01 (-0.02,-0.01)
Barbados	12.7 (9.9-16.5)	13.8 (10.7-17.7)	15.6 (12.2-20.1)	1.65 (1.28,2.13)	2.56 (1.98,3.29)	4.21 (3.26,5.43)

Supplementary Data 2. Sugar-sweetened beverage intakes (8 oz servings/week) in 1990, 2005, and 2018 and absolute change (8 oz servings/week) from 1990-2005, 2005-2018, and 1990-2018 in adults (20+years) globally, regionally, and nationally (continued).

	Mean intake (95% UI) (8 oz servings/week)			Absolute change (95% UI) (8 oz servings/week)		
	1990	2005	2018	1990-2005	2005-2018	1990-2018
Belarus	2.2 (1.5-3.1)	2.3 (1.6-3.4)	1.2 (0.8-1.7)	0.27 (-0.22,0.85)	-1.09 (-1.74,-0.64)	-0.81 (-1.36,-0.42)
Belgium	4.8 (4.3-5.3)	4.8 (4.3-5.3)	5.2 (4.7-5.9)	0.46 (0.28,0.65)	0.29 (0.17,0.41)	0.75 (0.45,1.07)
Belize	6.7 (4.5-9.9)	8.2 (5.6-12.1)	11.6 (7.9-17.1)	1.39 (0.94,2.07)	3.71 (2.51,5.53)	5.09 (3.45,7.59)
Benin	4.0 (2.6-6.3)	4.7 (3.0-7.4)	6.3 (4.0-9.8)	0.14 (0.08,0.24)	1.87 (1.01,3.17)	2.02 (1.09,3.41)
Bhutan	0.9 (0.5-1.7)	1.8 (1.0-3.5)	2.5 (1.3-4.9)	0.41 (0.06,1.09)	0.73 (0.19,1.75)	1.16 (0.53,2.47)
Bolivia	12.7 (9.6-16.8)	9.7 (7.3-12.9)	9.8 (7.4-13.0)	-3.08 (-4.14,-2.32)	0.21 (0.16,0.29)	-2.87 (-3.85,-2.17)
Bosnia and Herzegovina	0.5 (0.3-0.7)	1.7 (1.2-2.5)	1.8 (1.3-2.6)	1.25 (0.87,1.83)	0.15 (-0.18,0.53)	1.39 (0.98,2.01)
Botswana	5.8 (3.9-8.4)	10.5 (7.1-15.0)	14.2 (9.8-19.3)	3.35 (1.52,5.55)	4.35 (2.01,6.90)	7.75 (4.91,10.75)
Brazil	7.6 (6.7-8.7)	4.5 (4.0-5.1)	4.4 (3.9-4.9)	-2.97 (-3.38,-2.60)	0.07 (0.06,0.09)	-2.90 (-3.30,-2.54)
Brunei	2.2 (1.5-3.2)	3.9 (2.7-5.6)	4.4 (3.0-6.2)	1.67 (1.16,2.39)	0.77 (0.53,1.11)	2.44 (1.70,3.51)
Bulgaria	1.3 (1.0-1.6)	2.0 (1.5-2.5)	1.2 (0.9-1.5)	0.70 (0.54,0.90)	-0.68 (-0.88,-0.53)	0.01 (0.01,0.02)
Burkina Faso	0.6 (0.2-1.8)	2.0 (0.7-5.4)	3.4 (1.2-9.6)	1.19 (0.43,3.32)	1.56 (0.56,4.32)	2.75 (0.99,7.66)
Burundi	4.1 (2.7-6.4)	4.5 (3.0-6.8)	6.5 (4.4-10.1)	-0.06 (-1.64,1.48)	2.30 (0.58,4.85)	2.22 (0.48,4.75)
Cambodia	0.1 (0.1-0.2)	2.4 (1.9-3.0)	4.1 (3.2-5.2)	2.22 (1.75,2.84)	1.73 (1.31,2.28)	3.95 (3.09,5.10)
Cameroon	1.9 (1.3-2.8)	5.5 (3.7-8.0)	7.6 (5.2-11.1)	3.11 (1.95,4.88)	2.36 (0.94,4.24)	5.52 (3.63,8.16)
Canada	3.9 (3.6-4.3)	4.1 (3.7-4.5)	3.8 (3.5-4.1)	0.72 (0.58,0.87)	-0.47 (-0.57,-0.39)	0.24 (0.19,0.29)
Cape Verde	2.3 (1.6-3.4)	7.5 (5.1-10.9)	3.3 (2.2-4.9)	4.69 (2.88,7.44)	-3.94 (-6.51,-2.33)	0.71 (-0.02,1.71)
Central African Republic	1.7 (1.1-2.6)	4.9 (3.3-7.2)	1.9 (1.3-2.9)	2.83 (1.79,4.44)	-2.68 (-4.33,-1.66)	0.16 (-0.29,0.66)
Chad	3.5 (2.3-5.2)	4.2 (2.8-6.3)	5.1 (3.4-7.5)	0.42 (-0.54,1.56)	1.03 (-0.15,2.47)	1.45 (0.37,2.95)
Chile	5.7 (4.3-7.5)	7.6 (5.7-10.0)	8.3 (6.3-10.9)	1.97 (1.49,2.58)	0.99 (0.75,1.30)	2.96 (2.25,3.88)
China	0.3 (0.3-0.4)	0.3 (0.2-0.3)	0.2 (0.2-0.3)	-0.06 (-0.07,-0.04)	0.00 (0.00,0.00)	-0.06 (-0.08,-0.05)
Colombia	15.2 (11.6-19.8)	13.1 (10.0-17.1)	20.0 (15.1-25.7)	-1.81 (-2.38,-1.37)	7.32 (5.53,9.36)	5.50 (4.14,6.98)
Comoros	5.1 (3.5-7.5)	5.3 (3.7-7.7)	7.7 (5.2-11.0)	-0.19 (-1.55,1.05)	2.65 (1.17,4.80)	2.46 (0.97,4.50)
Congo, Dem. Rep.	1.5 (0.5-4.0)	1.8 (0.7-5.0)	1.9 (0.7-5.3)	0.29 (0.10,0.84)	0.10 (0.03,0.29)	0.38 (0.13,1.13)
Congo, Rep.	2.2 (1.5-3.2)	6.8 (4.6-10.0)	9.5 (6.4-13.4)	4.50 (2.86,6.92)	2.61 (0.76,4.80)	7.17 (4.73,10.25)
Costa Rica	9.3 (6.3-13.7)	8.1 (5.5-11.9)	6.1 (4.1-9.0)	-1.13 (-1.66,-0.76)	-1.82 (-2.66,-1.22)	-2.95 (-4.32,-1.97)
Cote d'Ivoire	3.5 (2.4-5.3)	5.6 (3.8-8.2)	6.1 (4.1-9.0)	1.43 (0.37,2.91)	0.90 (-0.46,2.58)	2.36 (1.09,4.14)

Supplementary Data 2. Sugar-sweetened beverage intakes (8 oz servings/week) in 1990, 2005, and 2018 and absolute change (8 oz servings/week) from 1990-2005, 2005-2018, and 1990-2018 in adults (20+years) globally, regionally, and nationally (continued).

	Mean intake (95% UI) (8 oz servings/week)			Absolute change (95% UI) (8 oz servings/week)		
	1990	2005	2018	1990-2005	2005-2018	1990-2018
Croatia	1.9 (1.5-2.4)	2.4 (1.9-3.0)	1.9 (1.5-2.4)	0.61 (0.47,0.79)	-0.40 (-0.52,-0.30)	0.21 (0.16,0.27)
Cuba	14.2 (10.7-18.5)	10.7 (8.0-14.4)	9.5 (7.1-12.8)	-2.84 (-5.11,-0.71)	-0.66 (-2.60,1.18)	-3.54 (-5.80,-1.47)
Cyprus	2.8 (1.4-5.6)	3.6 (1.8-7.1)	3.1 (1.6-6.2)	0.83 (0.42,1.66)	-0.40 (-0.79,-0.20)	0.44 (0.22,0.87)
Czech Republic	1.7 (1.4-2.1)	1.6 (1.3-1.9)	1.3 (1.1-1.6)	-0.13 (-0.16,-0.10)	-0.16 (-0.20,-0.13)	-0.29 (-0.36,-0.23)
Denmark	1.5 (1.3-1.8)	1.6 (1.3-1.9)	1.9 (1.6-2.2)	0.19 (0.16,0.24)	0.24 (0.20,0.30)	0.44 (0.35,0.54)
Djibouti	11.3 (7.5-16.2)	8.7 (5.8-12.8)	19.3 (13.6-25.4)	-3.14 (-6.08,-0.65)	11.16 (7.43,15.09)	7.96 (4.42,11.73)
Dominica	9.2 (6.2-13.7)	7.0 (4.7-10.4)	5.3 (3.5-7.9)	-2.00 (-2.98,-1.33)	-1.52 (-2.26,-1.01)	-3.51 (-5.24,-2.34)
Dominican Republic	3.8 (2.8-5.4)	10.0 (7.5-13.4)	7.2 (5.4-10.0)	6.01 (4.12,8.52)	-2.52 (-4.73,-0.63)	3.46 (2.08,5.38)
Ecuador	20.6 (16.4-25.1)	7.2 (5.2-10.1)	13.6 (10.3-17.9)	-12.93 (-15.76,-10.01)	6.52 (4.47,9.04)	-6.35 (-8.75,-3.93)
Egypt, Arab Rep.	3.0 (2.4-3.7)	2.8 (2.3-3.4)	2.8 (2.3-3.4)	-0.18 (-0.26,-0.11)	-0.03 (-0.04,-0.02)	-0.21 (-0.29,-0.14)
El Salvador	5.1 (3.5-7.5)	6.9 (4.7-10.2)	9.5 (6.5-13.9)	1.75 (1.19,2.57)	2.72 (1.84,3.98)	4.47 (3.03,6.54)
Equatorial Guinea	3.3 (2.2-4.8)	6.4 (4.3-9.4)	7.1 (4.7-10.6)	2.42 (1.02,4.35)	0.56 (-1.15,2.41)	3.01 (1.45,5.30)
Eritrea	5.4 (3.6-7.9)	6.2 (4.2-8.9)	9.3 (6.4-13.1)	0.03 (-1.38,1.54)	3.29 (1.51,5.62)	3.33 (1.64,5.59)
Estonia	1.2 (1.0-1.5)	1.8 (1.5-2.1)	1.3 (1.1-1.5)	0.60 (0.38,0.85)	-0.46 (-0.65,-0.29)	0.14 (0.09,0.20)
Ethiopia (excludes Eritrea)	3.6 (2.8-4.6)	5.7 (4.5-7.2)	7.1 (5.6-8.9)	1.68 (1.30,2.16)	1.67 (1.25,2.20)	3.34 (2.54,4.36)
Fiji	1.3 (1.0-1.9)	3.5 (2.5-5.0)	3.3 (2.4-4.7)	2.15 (1.44,3.28)	-0.10 (-0.93,0.69)	2.04 (1.34,3.11)
Finland	1.9 (1.7-2.2)	1.6 (1.4-1.8)	1.5 (1.4-1.7)	-0.17 (-0.23,-0.12)	-0.03 (-0.04,-0.02)	-0.21 (-0.27,-0.14)
France	2.1 (1.9-2.3)	2.4 (2.2-2.7)	2.8 (2.5-3.1)	0.54 (0.47,0.62)	0.36 (0.31,0.41)	0.90 (0.78,1.03)
Gabon	10.7 (7.3-15.1)	8.3 (5.5-12.2)	6.9 (4.6-10.4)	-4.25 (-7.26,-1.79)	-0.72 (-2.75,1.19)	-5.01 (-8.09,-2.56)
Gambia, The	20.6 (14.7-26.6)	7.6 (5.0-11.0)	5.9 (3.9-9.0)	-14.77 (-19.09,-10.26)	-1.21 (-3.40,0.59)	-16.04 (-20.37,-11.28)
Georgia	0.1 (0.1-0.1)	2.4 (1.7-3.5)	1.8 (1.2-2.5)	2.30 (1.64,3.26)	-0.62 (-1.17,-0.19)	1.68 (1.18,2.36)
Germany	2.8 (2.5-3.1)	2.8 (2.5-3.1)	2.7 (2.5-3.0)	0.41 (0.26,0.57)	-0.23 (-0.30,-0.16)	0.19 (0.10,0.27)
Ghana	5.0 (3.5-7.1)	6.8 (4.8-9.6)	7.4 (5.2-10.4)	1.18 (0.68,1.86)	0.85 (0.47,1.40)	2.03 (1.15,3.25)
Greece	2.1 (1.8-2.5)	2.2 (1.8-2.5)	2.0 (1.7-2.3)	0.09 (0.07,0.12)	-0.02 (-0.02,-0.01)	0.08 (0.05,0.11)
Grenada	13.9 (10.5-18.1)	9.1 (6.8-12.2)	5.9 (4.4-8.1)	-4.67 (-7.02,-2.59)	-3.06 (-4.92,-1.59)	-7.77 (-10.46,-5.51)
Guatemala	2.8 (2.3-3.5)	5.5 (4.5-6.8)	7.4 (5.9-9.1)	2.65 (2.14,3.28)	1.87 (1.50,2.32)	4.53 (3.63,5.61)
Guinea	1.3 (0.9-1.9)	5.2 (3.6-7.7)	2.2 (1.5-3.3)	3.66 (2.37,5.60)	-2.80 (-4.48,-1.69)	0.84 (0.37,1.51)

Supplementary Data 2. Sugar-sweetened beverage intakes (8 oz servings/week) in 1990, 2005, and 2018 and absolute change (8 oz servings/week) from 1990-2005, 2005-2018, and 1990-2018 in adults (20+years) globally, regionally, and nationally (continued).

	Mean intake (95% UI) (8 oz servings/week)			Absolute change (95% UI) (8 oz servings/week)		
	1990	2005	2018	1990-2005	2005-2018	1990-2018
Guinea-Bissau	0.0 (0.0-0.1)	5.0 (3.4-7.3)	7.4 (5.1-10.9)	4.73 (3.19,7.03)	2.56 (1.08,4.61)	7.35 (5.03,10.77)
Guyana	12.3 (8.3-18.3)	7.4 (5.0-11.1)	8.1 (5.5-12.1)	-4.34 (-6.44,-2.94)	0.96 (0.66,1.42)	-3.38 (-5.02,-2.28)
Haiti	3.2 (2.2-4.6)	6.3 (4.4-9.2)	3.3 (2.3-4.8)	3.08 (2.15,4.47)	-3.09 (-4.49,-2.16)	-0.01 (-0.02,-0.01)
Honduras	5.5 (4.4-7.1)	6.7 (5.2-8.5)	9.9 (7.8-12.7)	0.99 (0.78,1.27)	3.38 (2.64,4.34)	4.37 (3.41,5.60)
Hungary	2.4 (2.0-2.9)	1.9 (1.6-2.3)	1.8 (1.5-2.2)	-0.37 (-0.46,-0.29)	-0.06 (-0.07,-0.05)	-0.42 (-0.53,-0.33)
Iceland	3.5 (3.0-4.1)	3.0 (2.6-3.5)	3.8 (3.2-4.4)	-0.23 (-0.31,-0.15)	0.75 (0.45,1.08)	0.53 (0.30,0.77)
India	0.2 (0.1-0.2)	0.2 (0.1-0.3)	0.2 (0.1-0.3)	0.00 (0.00,0.00)	0.02 (0.01,0.03)	0.02 (0.01,0.03)
Indonesia	0.5 (0.4-0.6)	0.7 (0.6-1.0)	0.9 (0.7-1.2)	0.21 (0.16,0.28)	0.25 (0.19,0.34)	0.47 (0.35,0.62)
Iran, Islamic Rep.	2.8 (2.5-3.2)	2.8 (2.5-3.2)	2.7 (2.4-3.0)	0.10 (0.07,0.12)	-0.04 (-0.08,0.00)	0.06 (0.00,0.12)
Iraq	6.2 (4.3-9.5)	5.9 (4.0-9.0)	5.3 (3.6-8.0)	-0.41 (-0.66,-0.26)	-0.56 (-0.89,-0.35)	-0.97 (-1.55,-0.61)
Ireland	3.9 (3.3-4.8)	3.0 (2.5-3.7)	3.3 (2.7-4.0)	-0.71 (-0.90,-0.54)	0.27 (0.20,0.34)	-0.44 (-0.56,-0.34)
Israel	6.7 (6.0-7.5)	3.9 (3.4-4.5)	4.2 (3.7-4.8)	-2.53 (-2.95,-2.15)	0.35 (0.30,0.41)	-2.18 (-2.54,-1.84)
Italy	1.6 (1.5-1.8)	1.6 (1.4-1.8)	1.5 (1.4-1.7)	0.08 (0.07,0.10)	-0.02 (-0.03,-0.02)	0.06 (0.05,0.07)
Jamaica	10.7 (8.4-13.8)	10.8 (8.5-13.9)	9.2 (7.2-11.8)	0.15 (0.11,0.20)	-1.35 (-1.74,-1.06)	-1.20 (-1.55,-0.94)
Japan	2.5 (2.2-2.9)	2.2 (1.9-2.5)	1.9 (1.6-2.2)	-0.20 (-0.27,-0.14)	-0.10 (-0.13,-0.06)	-0.30 (-0.40,-0.21)
Jordan	6.6 (5.1-8.4)	10.6 (8.2-13.6)	7.1 (5.5-9.1)	3.97 (3.03,5.07)	-3.11 (-3.97,-2.39)	0.86 (0.65,1.11)
Kazakhstan	0.8 (0.6-1.2)	2.4 (1.7-3.6)	1.4 (1.0-2.0)	1.68 (1.15,2.49)	-1.07 (-1.73,-0.61)	0.61 (0.36,0.99)
Kenya	1.6 (1.0-2.6)	1.3 (0.8-2.1)	0.9 (0.6-1.5)	-0.49 (-0.80,-0.29)	-0.27 (-0.45,-0.17)	-0.76 (-1.25,-0.46)
Kiribati	1.1 (0.7-1.6)	2.1 (1.4-3.2)	2.0 (1.3-3.1)	1.03 (0.66,1.62)	-0.04 (-0.06,-0.02)	0.99 (0.64,1.56)
Korea, Rep.	1.1 (1.0-1.1)	1.0 (0.9-1.1)	0.9 (0.8-1.0)	0.04 (0.02,0.06)	0.01 (0.00,0.02)	0.05 (0.02,0.08)
Kuwait	4.0 (2.7-5.9)	15.4 (10.4-22.6)	9.9 (6.6-14.6)	10.36 (6.92,15.19)	-4.03 (-5.87,-2.69)	6.33 (4.22,9.34)
Kyrgyz Republic	3.5 (2.4-5.0)	2.3 (1.6-3.4)	4.6 (3.3-6.8)	-1.13 (-2.10,-0.41)	2.31 (1.36,3.75)	1.17 (0.20,2.44)
Lao PDR	0.5 (0.3-0.7)	1.1 (0.8-1.4)	1.5 (1.1-1.9)	0.60 (0.46,0.78)	0.39 (0.29,0.53)	0.99 (0.75,1.31)
Latvia	1.2 (1.0-1.4)	1.1 (0.9-1.3)	1.0 (0.9-1.2)	-0.01 (-0.02,0.00)	-0.01 (-0.02,0.00)	-0.02 (-0.02,-0.01)
Lebanon	6.8 (5.9-7.9)	4.3 (3.7-5.0)	6.3 (5.4-7.4)	-2.34 (-2.72,-2.02)	2.18 (1.88,2.54)	-0.16 (-0.19,-0.14)
Lesotho	4.8 (3.3-7.2)	6.0 (4.1-8.7)	14.3 (10.0-19.5)	0.12 (-1.13,1.37)	8.90 (5.92,12.41)	9.02 (6.06,12.52)
Liberia	2.3 (1.5-3.6)	3.9 (2.6-6.0)	9.7 (6.6-14.1)	1.52 (0.77,2.72)	5.88 (3.78,8.86)	7.47 (4.95,10.84)
Libya	5.3 (3.7-7.7)	6.7 (4.7-9.8)	5.7 (4.0-8.3)	1.39 (-0.02,3.10)	-0.70 (-2.27,0.74)	0.69 (-0.64,2.14)
Lithuania	1.1 (0.9-1.3)	1.2 (1.0-1.5)	1.2 (1.0-1.5)	0.23 (0.16,0.31)	0.02 (0.01,0.03)	0.25 (0.17,0.34)

Supplementary Data 2. Sugar-sweetened beverage intakes (8 oz servings/week) in 1990, 2005, and 2018 and absolute change (8 oz servings/week) from 1990-2005, 2005-2018, and 1990-2018 in adults (20+years) globally, regionally, and nationally (continued).

	Mean intake (95% UI) (8 oz servings/week)			Absolute change (95% UI) (8 oz servings/week)		
	1990	2005	2018	1990-2005	2005-2018	1990-2018
Luxembourg	2.9 (2.2-3.8)	3.3 (2.5-4.2)	3.5 (2.7-4.5)	0.56 (0.41,0.75)	0.08 (0.06,0.11)	0.64 (0.47,0.86)
Macedonia, FYR	1.0 (0.8-1.2)	2.7 (2.2-3.4)	6.0 (4.7-7.7)	1.78 (1.40,2.26)	3.40 (2.64,4.39)	5.18 (4.05,6.64)
Madagascar	5.7 (3.8-8.5)	4.0 (2.7-6.1)	4.9 (3.3-7.3)	-2.18 (-3.87,-1.03)	1.03 (0.07,2.32)	-1.14 (-2.67,0.13)
Malawi	2.5 (1.7-3.5)	2.4 (1.7-3.4)	1.2 (0.9-1.8)	-0.26 (-0.37,-0.18)	-1.06 (-1.52,-0.74)	-1.32 (-1.89,-0.92)
Malaysia	2.2 (1.7-2.8)	2.0 (1.6-2.6)	2.2 (1.7-2.7)	-0.16 (-0.23,-0.10)	0.27 (0.15,0.40)	0.11 (0.06,0.18)
Maldives	8.1 (5.0-12.1)	6.2 (3.8-10.1)	3.1 (1.9-5.3)	-3.26 (-4.19,-2.14)	-3.94 (-6.11,-2.39)	-7.24 (-10.09,-4.53)
Mali	1.6 (1.0-2.5)	4.5 (3.0-6.9)	2.4 (1.5-3.7)	2.60 (1.50,4.52)	-2.04 (-3.73,-0.95)	0.58 (-0.05,1.39)
Malta	3.9 (3.1-5.2)	5.1 (4.0-6.7)	6.2 (4.8-8.0)	1.12 (0.86,1.47)	1.44 (1.10,1.91)	2.57 (1.96,3.38)
Marshall Islands	1.2 (0.8-1.7)	3.1 (2.2-4.5)	2.9 (2.1-4.1)	1.86 (1.22,2.85)	-0.06 (-0.81,0.76)	1.80 (1.17,2.80)
Mauritania	3.6 (2.4-5.6)	7.9 (5.1-12.1)	10.8 (7.0-16.5)	3.65 (2.38,5.64)	3.27 (2.12,5.04)	6.93 (4.50,10.69)
Mauritius	26.5 (22.0-30.9)	17.1 (13.3-21.6)	20.4 (16.1-25.1)	-9.88 (-11.34,-8.50)	5.35 (4.46,6.31)	-4.53 (-5.17,-3.89)
Mexico	9.8 (9.0-10.7)	9.3 (8.4-10.2)	8.9 (8.2-9.8)	-0.44 (-0.58,-0.30)	0.02 (0.00,0.03)	-0.43 (-0.55,-0.30)
Micronesia, Fed. Sts.	1.2 (0.9-1.7)	3.8 (2.7-5.7)	3.3 (2.4-4.8)	2.58 (1.67,4.04)	-0.39 (-1.54,0.59)	2.19 (1.40,3.41)
Moldova	1.5 (1.1-2.2)	2.3 (1.6-3.3)	0.6 (0.4-0.9)	0.78 (0.35,1.38)	-1.63 (-2.36,-1.12)	-0.84 (-1.29,-0.56)
Mongolia	3.8 (2.7-5.3)	3.2 (2.3-4.5)	3.1 (2.1-4.3)	-0.53 (-0.94,-0.23)	-0.03 (-0.08,0.02)	-0.55 (-0.93,-0.29)
Montenegro	1.2 (0.6-2.7)	1.3 (0.6-3.0)	1.8 (0.8-4.1)	0.22 (0.10,0.50)	0.52 (0.23,1.18)	0.74 (0.33,1.68)
Morocco	4.3 (3.0-6.2)	5.0 (3.5-7.2)	4.8 (3.3-7.0)	0.89 (0.61,1.31)	-0.06 (-0.09,-0.04)	0.83 (0.56,1.23)
Mozambique	1.4 (0.9-2.3)	2.2 (1.4-3.5)	2.6 (1.6-4.1)	0.68 (0.38,1.17)	0.36 (0.19,0.65)	1.04 (0.57,1.82)
Myanmar	0.4 (0.3-0.7)	2.1 (1.5-3.0)	2.9 (2.1-4.2)	1.49 (1.02,2.28)	0.95 (0.30,1.85)	2.46 (1.73,3.64)
Namibia	16.1 (11.2-22.8)	13.1 (9.1-19.1)	14.7 (10.2-21.2)	-3.87 (-5.31,-2.51)	0.86 (0.54,1.26)	-3.00 (-4.08,-1.98)
Nepal	0.3 (0.1-0.5)	0.8 (0.4-1.5)	1.3 (0.7-2.6)	0.37 (0.18,0.79)	0.60 (0.28,1.26)	0.98 (0.50,1.95)
Netherlands	4.0 (3.7-4.4)	3.3 (3.1-3.6)	2.9 (2.7-3.2)	-0.33 (-0.43,-0.23)	-0.30 (-0.40,-0.19)	-0.63 (-0.83,-0.42)
New Zealand	1.2 (1.0-1.5)	2.3 (1.9-2.8)	1.8 (1.5-2.2)	1.13 (0.92,1.37)	-0.41 (-0.51,-0.34)	0.71 (0.58,0.87)
Nicaragua	3.7 (2.7-5.3)	9.7 (7.2-13.2)	11.9 (8.8-15.8)	5.72 (3.83,8.35)	2.49 (0.16,5.00)	8.21 (5.80,11.27)
Niger	1.7 (1.1-2.8)	3.2 (2.1-5.0)	0.0 (0.0-0.0)	1.30 (0.52,2.57)	-3.06 (-4.88,-1.97)	-1.75 (-2.78,-1.12)
Nigeria	0.5 (0.3-0.7)	4.8 (3.2-7.0)	4.9 (3.2-7.2)	4.15 (2.73,6.21)	0.16 (-1.02,1.42)	4.33 (2.85,6.46)
Norway	2.1 (1.9-2.4)	1.7 (1.5-1.9)	2.0 (1.8-2.3)	-0.25 (-0.30,-0.22)	0.23 (0.20,0.27)	-0.02 (-0.02,-0.02)
Oman	1.8 (1.2-2.7)	5.1 (3.4-7.6)	4.6 (3.1-6.9)	3.34 (2.21,5.05)	-0.53 (-0.80,-0.36)	2.81 (1.85,4.25)

Supplementary Data 2. Sugar-sweetened beverage intakes (8 oz servings/week) in 1990, 2005, and 2018 and absolute change (8 oz servings/week) from 1990-2005, 2005-2018, and 1990-2018 in adults (20+years) globally, regionally, and nationally (continued).

	Mean intake (95% UI) (8 oz servings/week)			Absolute change (95% UI) (8 oz servings/week)		
	1990	2005	2018	1990-2005	2005-2018	1990-2018
Pakistan	4.2 (2.6-6.9)	5.2 (3.3-8.6)	4.2 (2.6-7.0)	-0.03 (-0.05,-0.02)	-0.38 (-0.62,-0.24)	-0.42 (-0.67,-0.26)
Palestine	3.4 (2.4-4.9)	4.5 (3.1-6.5)	4.6 (3.1-6.6)	1.14 (0.78,1.66)	0.07 (0.04,0.09)	1.21 (0.82,1.75)
Panama	11.5 (8.6-15.3)	8.7 (6.4-11.9)	13.7 (10.5-17.8)	-2.64 (-4.64,-0.81)	5.27 (3.23,7.66)	2.64 (0.55,4.83)
Papua New Guinea	1.0 (0.7-1.5)	3.1 (2.2-4.6)	0.0 (0.0-0.0)	1.98 (1.23,3.19)	-2.92 (-4.36,-2.05)	-0.93 (-1.42,-0.66)
Paraguay	5.6 (4.1-7.9)	8.3 (6.1-11.4)	9.6 (7.1-13.0)	2.57 (1.19,4.38)	1.40 (-0.44,3.30)	3.97 (2.34,6.09)
Peru	6.9 (4.7-10.2)	5.7 (3.8-8.4)	6.2 (4.2-9.2)	-1.18 (-1.77,-0.80)	0.79 (0.53,1.20)	-0.39 (-0.58,-0.26)
Philippines	3.8 (3.3-4.4)	3.4 (3.0-4.0)	3.2 (2.8-3.7)	-0.23 (-0.28,-0.18)	-0.01 (-0.02,-0.01)	-0.24 (-0.29,-0.20)
Poland	1.4 (1.2-1.5)	1.3 (1.1-1.4)	1.2 (1.1-1.3)	-0.02 (-0.04,0.00)	-0.01 (-0.02,0.00)	-0.03 (-0.04,-0.02)
Portugal	2.7 (2.4-3.0)	3.3 (2.9-3.6)	2.7 (2.4-2.9)	0.60 (0.53,0.68)	-0.36 (-0.40,-0.32)	0.24 (0.21,0.27)
Qatar	5.4 (3.6-8.1)	7.1 (4.7-10.5)	6.2 (4.1-9.2)	2.00 (1.32,3.00)	-1.15 (-1.72,-0.75)	0.85 (0.56,1.28)
Romania	3.7 (3.0-4.4)	3.7 (3.1-4.4)	3.4 (2.8-4.0)	0.11 (0.06,0.17)	-0.05 (-0.08,-0.04)	0.06 (0.02,0.10)
Russian Federation	1.1 (0.9-1.3)	2.4 (2.0-2.9)	2.3 (1.9-2.8)	1.35 (1.11,1.64)	-0.06 (-0.07,-0.04)	1.29 (1.06,1.58)
Rwanda	1.2 (0.7-2.1)	2.2 (1.3-3.7)	34.2 (30.1-36.0)	0.62 (0.14,1.40)	32.12 (28.70,33.80)	32.82 (29.18,34.45)
Samoa	2.3 (1.5-3.5)	6.2 (4.1-9.3)	6.8 (4.5-10.3)	3.74 (2.46,5.66)	0.95 (0.62,1.43)	4.69 (3.08,7.09)
Sao Tome and Principe	2.7 (1.8-4.0)	7.1 (4.8-10.4)	4.7 (3.1-6.9)	3.78 (2.19,6.31)	-2.17 (-4.44,-0.70)	1.57 (0.54,3.09)
Saudi Arabia	4.3 (3.0-6.3)	6.5 (4.5-9.4)	6.1 (4.3-8.9)	2.26 (0.98,4.04)	-0.09 (-1.72,1.48)	2.19 (0.91,3.85)
Senegal	10.1 (6.8-14.5)	4.4 (2.9-6.7)	18.8 (13.4-24.5)	-5.69 (-8.65,-3.36)	13.97 (9.77,18.40)	8.16 (4.65,11.80)
Serbia	1.5 (0.8-3.0)	1.9 (1.0-3.8)	0.9 (0.5-1.8)	0.47 (0.24,0.95)	-0.95 (-1.90,-0.48)	-0.47 (-0.95,-0.24)
Seychelles	8.2 (6.5-10.2)	8.9 (7.3-11.0)	8.6 (6.9-10.8)	0.05 (-0.03,0.15)	0.50 (0.28,0.75)	0.55 (0.34,0.81)
Sierra Leone	2.4 (1.6-3.8)	3.4 (2.2-5.3)	8.4 (5.5-12.5)	0.72 (-0.04,1.74)	5.08 (3.04,7.94)	5.82 (3.65,8.96)
Singapore	3.1 (2.5-3.8)	2.8 (2.3-3.5)	2.6 (2.1-3.2)	0.09 (0.03,0.15)	-0.03 (-0.05,-0.01)	0.06 (0.02,0.11)
Slovak Republic	2.6 (2.2-3.1)	2.9 (2.5-3.4)	3.4 (2.8-4.0)	0.39 (0.25,0.53)	0.54 (0.29,0.83)	0.93 (0.54,1.36)
Slovenia	2.2 (1.7-2.7)	2.7 (2.2-3.4)	2.7 (2.1-3.3)	0.66 (0.48,0.88)	0.11 (0.06,0.17)	0.78 (0.55,1.04)
Solomon Islands	4.4 (2.9-6.7)	4.6 (3.0-7.0)	3.5 (2.3-5.3)	0.05 (0.02,0.09)	-0.89 (-1.50,-0.51)	-0.85 (-1.41,-0.48)
South Africa	11.5 (9.1-15.0)	7.8 (6.1-10.1)	9.4 (7.4-12.2)	-4.94 (-6.46,-3.86)	2.20 (1.71,2.89)	-2.74 (-3.58,-2.15)
South Sudan	4.5 (3.1-6.7)	5.2 (3.5-7.5)	7.5 (5.1-10.9)	0.11 (-1.09,1.40)	2.65 (1.16,4.75)	2.75 (1.20,4.97)
Spain	2.6 (2.2-3.1)	2.9 (2.4-3.4)	3.1 (2.6-3.7)	0.50 (0.41,0.59)	0.25 (0.21,0.30)	0.75 (0.62,0.90)

Supplementary Data 2. Sugar-sweetened beverage intakes (8 oz servings/week) in 1990, 2005, and 2018 and absolute change (8 oz servings/week) from 1990-2005, 2005-2018, and 1990-2018 in adults (20+years) globally, regionally, and nationally (continued).

	Mean intake (95% UI) (8 oz servings/week)			Absolute change (95% UI) (8 oz servings/week)		
	1990	2005	2018	1990-2005	2005-2018	1990-2018
Sri Lanka	2.8 (2.1-3.8)	7.5 (5.7-9.7)	6.1 (4.6-8.1)	4.14 (3.11,5.33)	-0.64 (-0.79,-0.49)	3.50 (2.61,4.58)
St. Lucia	9.5 (7.1-12.9)	9.1 (6.7-12.4)	8.7 (6.5-12.0)	-0.42 (-2.39,1.53)	0.12 (-1.77,2.04)	-0.27 (-2.27,1.59)
St. Vincent and the Grenadines	9.2 (6.8-12.4)	10.1 (7.6-13.4)	28.2 (24.2-31.8)	1.02 (-0.57,2.80)	18.39 (15.65,20.81)	19.44 (16.62,21.82)
Sudan	6.4 (4.2-10.0)	7.0 (4.6-10.8)	6.6 (4.3-10.3)	-0.01 (-0.03,0.01)	0.10 (0.04,0.19)	0.09 (0.04,0.17)
Suriname	6.7 (4.5-10.0)	16.3 (11.0-24.3)	9.3 (6.3-14.0)	9.43 (6.31,14.02)	-6.46 (-9.58,-4.33)	2.97 (1.98,4.42)
Swaziland	8.6 (5.9-12.5)	3.1 (2.1-4.5)	10.4 (7.1-15.2)	-5.32 (-7.75,-3.64)	6.98 (4.77,10.20)	1.66 (1.12,2.45)
Sweden	2.4 (2.2-2.6)	2.2 (2.0-2.4)	2.2 (2.0-2.5)	-0.06 (-0.07,-0.05)	-0.02 (-0.03,-0.01)	-0.08 (-0.10,-0.06)
Switzerland	2.9 (2.5-3.5)	3.2 (2.7-3.8)	3.0 (2.6-3.6)	0.47 (0.38,0.59)	-0.17 (-0.21,-0.14)	0.30 (0.24,0.38)
Syrian Arab Republic	2.7 (2.0-3.6)	3.8 (2.8-5.0)	3.5 (2.6-4.7)	1.08 (0.80,1.44)	-0.10 (-0.13,-0.07)	0.98 (0.72,1.31)
Taiwan	6.2 (5.1-7.6)	4.7 (3.8-5.7)	4.4 (3.6-5.4)	-0.98 (-1.20,-0.79)	0.22 (0.18,0.27)	-0.76 (-0.93,-0.61)
Tajikistan	1.0 (0.7-1.5)	2.0 (1.3-3.1)	4.2 (2.8-6.1)	0.98 (0.50,1.75)	2.15 (1.21,3.59)	3.15 (2.06,4.82)
Tanzania	0.5 (0.3-0.8)	4.9 (3.3-7.3)	7.9 (5.4-11.5)	4.13 (2.78,6.17)	3.21 (1.61,5.36)	7.38 (5.04,10.71)
Thailand	0.9 (0.4-1.8)	2.9 (1.4-6.0)	4.6 (2.2-9.3)	1.78 (0.87,3.65)	2.09 (1.03,4.32)	3.87 (1.89,7.94)
Timor-Leste	0.5 (0.4-0.9)	1.4 (1.0-2.2)	15.8 (11.9-20.2)	0.88 (0.56,1.44)	14.34 (10.78,18.25)	15.22 (11.48,19.47)
Togo	1.8 (1.2-2.8)	4.1 (2.7-6.2)	29.0 (24.1-32.7)	1.96 (1.14,3.21)	24.94 (20.99,27.93)	27.00 (22.58,30.21)
Tonga	1.8 (1.2-2.7)	5.5 (3.7-8.1)	5.6 (3.8-8.3)	3.57 (2.40,5.34)	0.35 (0.23,0.52)	3.92 (2.64,5.87)
Trinidad and Tobago	9.4 (6.4-13.9)	13.5 (9.2-20.1)	11.1 (7.6-16.5)	4.22 (2.85,6.27)	-1.83 (-2.72,-1.24)	2.38 (1.60,3.56)
Tunisia	4.2 (3.0-6.3)	6.5 (4.6-9.4)	7.0 (4.9-10.0)	2.39 (1.20,4.05)	0.64 (-0.72,2.19)	3.03 (1.72,4.82)
Turkey	3.2 (2.5-4.2)	2.2 (1.7-2.8)	2.8 (2.1-3.6)	-0.88 (-1.15,-0.68)	0.67 (0.52,0.89)	-0.21 (-0.27,-0.16)
Turkmenistan	18.0 (13.4-22.7)	1.6 (1.0-2.4)	0.5 (0.4-0.9)	-15.57 (-19.66,-11.68)	-0.97 (-1.57,-0.62)	-16.55 (-21.04,-12.40)
Uganda	0.2 (0.1-0.3)	4.2 (2.8-6.3)	11.0 (7.5-15.6)	3.96 (2.64,5.91)	6.80 (4.30,10.20)	10.81 (7.40,15.29)
Ukraine	2.0 (1.6-2.6)	1.8 (1.4-2.3)	1.3 (1.1-1.7)	-0.15 (-0.21,-0.12)	-0.44 (-0.57,-0.35)	-0.60 (-0.77,-0.46)
United Arab Emirates	5.0 (3.3-7.6)	7.0 (4.7-10.7)	3.5 (2.4-5.4)	1.94 (1.29,2.97)	-3.28 (-5.02,-2.19)	-1.34 (-2.05,-0.89)
United Kingdom	4.7 (4.3-5.2)	4.0 (3.6-4.4)	4.4 (4.0-4.8)	-0.25 (-0.32,-0.18)	0.10 (0.06,0.14)	-0.15 (-0.18,-0.12)
United States	5.8 (5.5-6.1)	7.7 (7.3-8.2)	4.9 (4.6-5.1)	2.27 (2.11,2.44)	-2.79 (-3.00,-2.60)	-0.52 (-0.55,-0.48)

Supplementary Data 2. Sugar-sweetened beverage intakes (8 oz servings/week) in 1990, 2005, and 2018 and absolute change (8 oz servings/week) from 1990-2005, 2005-2018, and 1990-2018 in adults (20+years) globally, regionally, and nationally (continued).

	Mean intake (95% UI) (8 oz servings/week)			Absolute change (95% UI) (8 oz servings/week)		
	1990	2005	2018	1990-2005	2005-2018	1990-2018
Uruguay	9.0 (6.2-13.2)	10.4 (7.1-15.3)	10.9 (7.4-15.9)	1.34 (0.79,2.14)	0.65 (0.37,1.06)	1.99 (1.15,3.20)
Uzbekistan	5.2 (3.6-7.7)	1.5 (1.0-2.3)	3.1 (2.1-4.7)	-3.45 (-5.21,-2.28)	1.63 (0.97,2.66)	-1.79 (-3.26,-0.78)
Vanuatu	1.3 (0.9-2.0)	2.3 (1.5-3.5)	2.3 (1.5-3.5)	0.90 (0.59,1.38)	0.10 (0.06,0.15)	1.00 (0.65,1.53)
Venezuela	10.0 (7.3-13.9)	10.2 (7.5-13.9)	5.3 (3.9-7.5)	0.15 (-2.12,2.50)	-4.30 (-6.78,-2.27)	-4.14 (-6.62,-2.20)
Vietnam	1.3 (1.0-1.7)	1.8 (1.4-2.3)	1.6 (1.2-2.0)	0.49 (0.31,0.70)	-0.11 (-0.15,-0.08)	0.38 (0.23,0.55)
Yemen, Rep.	11.4 (6.3-20.8)	15.3 (8.4-26.6)	15.2 (8.4-26.6)	4.04 (2.23,6.32)	-0.23 (-0.35,-0.12)	3.81 (2.10,6.01)
Zambia	15.5 (11.0-20.5)	5.8 (3.9-8.6)	4.7 (3.2-6.9)	-10.37 (-13.88,-7.20)	-0.60 (-1.82,0.45)	-11.03 (-14.58,-7.72)
Zimbabwe	3.9 (2.7-5.7)	8.6 (5.7-12.5)	11.2 (7.8-15.6)	3.91 (2.32,6.02)	3.28 (1.37,5.58)	7.23 (4.77,10.29)

[*] Data are mean intakes (95% UI) or mean absolute change in intakes (95% UI) in 8 oz servings per day. All intakes are reported adjusted to 2,000 kcal/d for ages 20 to 74 years, and 1,700 kcal/d for ages 75+ years. Data are based on a Bayesian model that incorporated up to 451 individual-level dietary surveys, and additional survey-level and country-level covariates, to estimate dietary consumption levels. Total SSBs intake was defined as any beverage with added sugars having ≥50 kcal per 8 oz serving, including commercial or homemade beverages, soft drinks, energy drinks, fruit drinks, punch, lemonade, and aguas frescas. This definition excludes 100% fruit and vegetable juices and non-caloric artificially sweetened drinks. Standardized serving size used for this analysis: 8 oz serving = 248 grams. Source data are provided as Source Data files 1, 2, 6, and 7.

[†] In prior GDD reports, the region Central/ Eastern Europe and Central Asia was referred as Former Soviet Union, and Southeast and East Asia was referred as Asia.

GDD, Global Dietary Database; oz, ounces; SSB, sugar-sweetened beverage; UI, uncertainty interval
