# Supplementary material for: Sugar-sweetened beverage intakes among adults between 1990 and 2018 in 185 countries
Source: Nat Commun. 2023 Oct 3;14:5957. doi: 10.1038/s41467-023-41269-8 (PMC10614169; doi:10.1038/s41467-023-41269-8)
Supplement: Supplementary file 7 — Reporting Summary [file 41467_2023_41269_MOESM7_ESM.pdf]

nature portfolio

Corresponding author(s): Laura Lara-Castor

Last updated by author(s): 2023/08/21

Reporting Summary

Nature Portfolio wishes to improve the reproducibility of the work that we publish. This form provides structure for consistency and transparency in reporting. For further information on Nature Portfolio policies, see our Editorial Policies and the Editorial Policy Checklist.

Statistics

For all statistical analyses, confirm that the following items are present in the figure legend, table legend, main text, or Methods section.

n/a	Confirmed	
☐	☒	The exact sample size (*n*) for each experimental group/condition, given as a discrete number and unit of measurement
☒	☐	A statement on whether measurements were taken from distinct samples or whether the same sample was measured repeatedly
☐	☒	The statistical test(s) used AND whether they are one- or two-sided<br>*Only common tests should be described solely by name; describe more complex techniques in the Methods section.*
☒	☐	A description of all covariates tested
☐	☒	A description of any assumptions or corrections, such as tests of normality and adjustment for multiple comparisons
☐	☒	A full description of the statistical parameters including central tendency (e.g. means) or other basic estimates (e.g. regression coefficient) AND variation (e.g. standard deviation) or associated estimates of uncertainty (e.g. confidence intervals)
☐	☒	For null hypothesis testing, the test statistic (e.g. *F*, *t*, *r*) with confidence intervals, effect sizes, degrees of freedom and *P* value noted<br>*Give P values as exact values whenever suitable.*
☐	☒	For Bayesian analysis, information on the choice of priors and Markov chain Monte Carlo settings
☒	☐	For hierarchical and complex designs, identification of the appropriate level for tests and full reporting of outcomes
☒	☐	Estimates of effect sizes (e.g. Cohen's *d*, Pearson's *r*), indicating how they were calculated

Our web collection on statistics for biologists contains articles on many of the points above.

Software and code

Policy information about availability of computer code

Data collection

No software was used for data collection for this analysis.

Data analysis

Custom code was developed using R (Version 4.0.0) for analyzing the data including aggregation of the 4,000 simulations to the strata categories, calculation of absolute and relative differences, summary of mean intakes globally, regionally, and nationally jointly stratified by sociodemographic group, and data visualizations including tables and figures. Given the computational size, the data aggregation, calculation of absolute and relative differences, and summary of mean intakes were run on the Tufts University High Performance Computing Cluster (https://it.tufts.edu/high-performance-computing), supported by the National Science Foundation (grant:2018149) under active development by Research Technology, Tufts Technology Services.

The statistical code can be made available to researchers upon request. Eligibility criteria for such requests include: utilization for nonprofit purposes only, for appropriate scientific use based on a robust research plan, and by investigators from an academic institution. GDD will nominate co-authors to be included on any papers generated using GDD-generated statistical code. If you are interested in requesting access to the statistical code, please submit the following documents:

(1) Proposed Research Plan: Please download and complete the Proposed Research plan form: https://www.globaldietarydatabase.org/sites/default/files/manual_upload/research-proposal-template.pdf
(2) Data Sharing Agreement: Please download this form https://www.globaldietarydatabase.org/sites/default/files/manual_upload/tufts-gdd-data-sharing-agreement.docx and complete the highlighted fields, have someone who is authorized to enter your institution into a binding legal agreement with outside institutions sign the document. Note that this agreement does not apply when Protected Health Information or Personally Identifiable Information are shared.
(3) Email items (1) and (2) to info@globaldietarydatabase.org. Please use the subject line "GDD Code Access Request".

Once all documents have been received, the GDD team will be in contact with you within 2-4 weeks regarding subsequent steps. Data will be shared as .csv or .xlsx files, using a compressed format when appropriate.

For manuscripts utilizing custom algorithms or software that are central to the research but not yet described in published literature, software must be made available to editors and reviewers. We strongly encourage code deposition in a community repository (e.g. GitHub). See the Nature Portfolio guidelines for submitting code & software for further information.

Data

Policy information about availability of data

All manuscripts must include a data availability statement. This statement should provide the following information, where applicable:

- Accession codes, unique identifiers, or web links for publicly available datasets
- A description of any restrictions on data availability
- For clinical datasets or third party data, please ensure that the statement adheres to our policy

The individual SSB intake estimate distribution data used in this analysis as means and uncertainty (SE) for each strata in the analysis are available freely online at the (GDD, Download 2018 Final Estimates: https://www.globaldietarydatabase.org/data-download). GDD data collapsed for 85+ and by age categories 20-39, 40-59, and 60+, as well as the absolute and relative differences by strata and by year presented in this analysis, were calculated using the 4,000 simulations corresponding to the stratum level intake data derived from the Bayesian model. The derived source data are provided with this paper. The 4,000 simulations files can be made available to researchers upon request. Eligibility criteria for such requests include utilization for nonprofit purposes only, for appropriate scientific use based on a robust research plan, and by investigators from an academic institution. If you are interested in requesting access to the statistical code, please submit the following documents:

(1) Proposed Research Plan: Please download and complete the Proposed Research plan form: https://www.globaldietarydatabase.org/sites/default/files/manual_upload/research-proposal-template.pdf
(2) Data Sharing Agreement: Please download this form https://www.globaldietarydatabase.org/sites/default/files/manual_upload/tufts-gdd-data-sharing-agreement.docx and complete the highlighted fields, have someone who is authorized to enter your institution into a binding legal agreement with outside institutions sign the document. Note that this agreement does not apply when Protected Health Information or Personally Identifiable Information are shared.
(3) Email items (1) and (2) to info@globaldietarydatabase.org. Please use the subject line "GDD Code Access Request".

Once all documents have been received, the GDD team will be in contact with you within 2-4 weeks regarding subsequent steps. Data will be shared as .csv or .xlsx files, using a compressed format when appropriate.

Population weights for each strata and year were derived from the United Nations Population Division (https://population.un.org/wpp/), supplemented with data for education and urban or rural status from Barro (DOI: 0.3386/w15902) and Lee and the United Nations (https://population.un.org/wup/Download/).

Human research participants

Policy information about studies involving human research participants and Sex and Gender in Research.

Reporting on sex and gender	N/A
Population characteristics	N/A
Recruitment	N/A
Ethics oversight	N/A

Note that full information on the approval of the study protocol must also be provided in the manuscript.

Field-specific reporting

Please select the one below that is the best fit for your research. If you are not sure, read the appropriate sections before making your selection.

☐ Life sciences ☒ Behavioural & social sciences ☐ Ecological, evolutionary & environmental sciences

For a reference copy of the document with all sections, see nature.com/documents/nr-reporting-summary-flat.pdf

Behavioural & social sciences study design

All studies must disclose on these points even when the disclosure is negative.

Study description	This investigation is based on serial cross-sectional analysis of SSB intakes using new quantitative data from the Global Dietary Database. We investigated global SSB intakes and trends among adults (20+ years) in 1990, 2005, and 2018 in 185 countries, stratified subnationally by age, sex, education, and rural/urban residence.

Research sample	Global adult population (20+ years), stratified by sex (female and male), education level (low 0-6, medium >6-12, high >12 years of education) and area of residence (urban and rural) corresponding to 31,080 population strata across 185 countries in each year (1990, 2005, and 2018). Population weights for each strata and year were derived from the United Nations Population Division, supplemented with data for education and urban or rural status from Barro and Lee and the United Nations. The model that derived intakes for each of these strata incorporated 1,225 surveys, from which 451 surveys included data on sugar-sweetened beverage intakes. The 451 surveys with sugar-sweetened beverage data came from 118 countries, with a global sample size of 2,941,704 participants: 44.3% female and 55.7% male; 70% urban areas and 30% from rural areas; 16% low, 37.6% medium, and 46.4% high education; and 53% adults and 47% children/adolescent. Characteristics by world region are available in Supplementary Table 4.<br><br>This investigation was based on published de-identified nationally representative data, without personally identifiable information. Individual surveys underwent ethical review board approval required for the applicable local context.
Sampling strategy	NA
Data collection	The individual SSB intake estimate distribution data used in this analysis is publicly available as mean and 95% UIs from the Global Dietary Database (GDD)- Download 2018 Final Estimates: https://www.globaldietarydatabase.org/data-download.
Timing	The estimates reported in this study correspond to years 1990, 2005, and 2018. The surveys informing the GDD model range from 1980-2018.
Data exclusions	3 countries excluded from GDD prediction models due to lack of FAO food availability covariate data (Andorra, North Korea, and Somalia)
Non-participation	NA
Randomization	NA

Reporting for specific materials, systems and methods

We require information from authors about some types of materials, experimental systems and methods used in many studies. Here, indicate whether each material, system or method listed is relevant to your study. If you are not sure if a list item applies to your research, read the appropriate section before selecting a response.

Materials & experimental systems

n/a	Involved in the study
☒	☐ Antibodies
☒	☐ Eukaryotic cell lines
☒	☐ Palaeontology and archaeology
☒	☐ Animals and other organisms
☒	☐ Clinical data
☒	☐ Dual use research of concern

Methods

n/a	Involved in the study
☒	☐ ChIP-seq
☒	☐ Flow cytometry
☒	☐ MRI-based neuroimaging

March 2021
